# Facilitators and challenges to exclusive breastfeeding in Belagavi District, Karnataka, India

**DOI:** 10.1371/journal.pone.0231755

**Published:** 2020-05-04

**Authors:** Umesh Charantimath, Roopa Bellad, Niranjana Majantashetti, Yukiko Washio, Richard Derman, Patricia J. Kelly, Vanessa Short, Esther Chung, Shivaprasad Goudar

**Affiliations:** 1 Jawaharlal Nehru Medical College, Belagavi, Karnataka, India; 2 RTI International, Research Triangle Park, Durham, North Carolina, United States of America; 3 Thomas Jefferson University, Philadelphia, Pennsylvania, United States of America; 4 University of Missouri-Kansas City, Kansas City, Missouri, United States of America; Kwame Nkrumah University of Science and Technology, GHANA

## Abstract

**Objective:**

A primary objective of this study was to identify specific facilitators and challenges around exclusive breastfeeding (EBF) in our community in India, from the perspective of breastfeeding mothers and their support networks.

**Methods:**

We conducted eight focus groups incorporating 75 women and their support networks in the Belagavi District, Karnataka State, India. We used a directed content analysis to guide the analysis.

**Results:**

The specific facilitator that emerged as a theme, *broad support for and knowledge about breastfeeding on the individual*, *family and community levels*, was a seeming contraction to the identified specific challenge, *the paradox of the common practice of supplemental feeds*.

**Conclusions:**

Despite voicing strong support for and knowledge about EBF, participants were familiar with a variety of supplemental feeding practices in their communities. In place of universal condemnation of all supplemental feeding, policy makers might consider strategies to address the most potentially dangerous of these practices.

## Background

Exclusive breastfeeding (EBF) is an important public health intervention for child health and survival. The World Health Organization (WHO) recommends that mothers initiate breastfeeding within the first hour of life; practice exclusive breastfeeding–that is the infant only receives breast milk without any additional food or drink, not even water, for six months; provide breastfeeding on demand–that is as often as the child wants, day and night, use no bottles, teats or pacifiers [1]. EBF compared to the use of formula saves money and benefits mothers and infants by protecting against infant mortality for the first 6 months, diarrhea, obesity type 1 and 2 diabetes mellitus, gastrointestinal illness, otitis media, respiratory infections, and hospitalization [2,3,4].

EBF is a global child health survival strategy to provide healthy and optimal nutrition, which is essential for normal growth and development during infancy and early childhood. With an EBF rate of 90%, an estimated 13–15% of deaths in children under five could be averted in low and middle income countries [5]. Achieving an EBF rate of >50% could save 520,000 under five children and generate an increased revenue of US $300 billion over 10 years as a result of reduced health costs [5].

Despite the significant results of this natural intervention, and extensive efforts by WHO since 1990, EBF is not practiced by millions of women around the world. The 2019 Global Breastfeeding Score card, which evaluated 194 nations, found that the average EBF rates were around 40%, with only 23 countries have EBF rates above 60% [6]. In India, the EBF rate was 56.3% at four months; while a definite increase from the 36.8% in the year 2000, the lack of this practice among almost half of Indian babies is troubling [6]. Even these rates are subject to some skepticism; social desirability factors, anecdotal provider reports, and the reality that most of these statistics are collected through maternal self-report can easily result in inflated numbers [7].

EBF is impacted by multiple personal, family, cultural and structural factors. Knowledge, health conditions, the attitudes of immediate and extended family, traditional practices, the availability of peer or community health worker support, and workplace policies such as the time and privacy to feed or pump breastmilk, all influence a woman’s ability to initiate and maintain EBF [8]. In preparation for a future intervention and to identify specific challenges and facilitators around EBF in our larger community in India, we conducted a series of focus groups with breastfeeding and non-breastfeeding mothers, mothers’ support networks, and healthcare providers working with young families. Data from our qualitative study will assure that our intervention is culturally appropriate and targets the unique needs of our target population. Our approach builds on the identified facilitators and target identified challenges in intervention delivery. Such information from the target area, to our knowledge, has not been previously collected.

## Methodology

Eight focus groups were conducted in the Belagavi District, Karnataka State, India among four different populations: women who had exclusively breastfed for at least six months, women who did not practice exclusive breastfeeding, members of breastfeeding mothers support networks, and health care providers. The study was approved by KAHERs J N Medical College Institutional Ethics Committee (Reference letter no. KLEU/EC/2017-18/D 1077). Ethical approval from Karnataka State Government and Health Minister Screening Committee were obtained. The trial was registered under Clinical Trial Registry of India with registration number CTRI/2017/08/009453. The approval from the Institutional Review Board was obtained from Health Ministry’s Screening Committee, Indian Council of Medical research.

### Sample/setting

Twenty-seven mothers who had successfully breastfed at least one child in the past three years and 19 members of the breastfeeding mother’s support network were recruited as participants in five focus groups. Nine women who were not able to successfully breastfeed due to some problem were specifically recruited for an eighth group. Twenty health care providers participated in two additional groups. Recruitment occurred through snowball technique and posters in five urban and rural primary health centers (PHC) in the Belagavi District associated with the Women and Children’s Research Unit at the medical school at which the Indian authors worked. The groups were conducted between January and August 2018, lasted 60–90 minutes and were held in a private room in the PHCs or in Women’s and Children’s Research Unit, Belagavi.

### Discussion guide

The focus group discussion guide centered on breastfeeding support, actual feeding practices, including prelactal (pre-breastfeeding) feeding, and psychosocial and behavioral factors that potentially facilitate or challenge EBF.

### Data collection

Trained investigators explained the purpose of the study, answered questions, and obtained signed consent from interested participants on forms written in Kannada, the most common language in the Belagavi District. The voluntary nature of participation in the research was stressed. All discussions were conducted in Kannada and lasted for 60 to 90 minutes. Participants received lunch and compensation for their travel expenses.

### Data analysis

Researchers transcribed audio-recordings and translated them verbatim from Kannada to English. All translated transcriptions were analyzed using NVivo software version 10. We used a directed content analysis to develop an initial list of codes [9]. Participant responses were compared across groups to determine the extent of convergence or discrepancies on major themes. Common themes were identified and initially organized into constructs by one author (PK) and confirmed by a second (YW). The remaining authors reviewed the analysis and their comments integrated into the results [10].

## Results

Of the 75 participants, 27 were breastfeeding women, 9 were mothers who had had breastfeeding problems, 17 were members of mothers’ support networks, and 20 were health care providers. Among the breastfeeding women and their support networks, the majority had completed secondary (30/54.5%), 13 (23.6%) had completed primary school, and 8 (14.5) were illiterate (Table 1). The two focus groups of 20 health care providers included three physicians, three nurses, six health assistants, eight community workers.

**Table 1:** Characteristics of mothers and support network focus group participants.

Type of Focus Group	Place	Education Status N = 55			
		Illiterate N = 8 (14.5%)	Primary School N = 13 (23.6%)	Secondary School N = 30 (54.5%)	University N = 3 (5.4%)
Breastfeeding mothers (n = 8)	PHC Belawadi	0	1	7	0
Breastfeeding mothers (n = 10)	PHC Kanagala	1	3	5	1
Breastfeeding mothers (n = 9)	PHC Ventamuri	0	1	6	2
Mothers with problems breastfeeding (n = 9)	JNMC Belagavi	1	0	8	0
Breastfeeding mothers’ support network (n = 10)	PHC Kanagala	3	4	3	0
Breastfeeding mother’s support network (n = 9)	PHC Belawadi	4	4	1	0

The objective of the study was to identify specific facilitators and challenges around EBF in our larger community in India. Two somewhat contradictory themes about breastfeeding were clear from the analysis. A specific facilitator was the *broad knowledge about and support for breastfeeding from mothers and support network participants*. Health care providers, were particularly supportive of EBF. A paradoxical challenge mentioned in each focus group was the *common practice of supplemental feeds*, as well as a variety of psychosocial and physical issues.

### Facilitators of EBF

#### Responses from mothers with breastfeeding experiences

Participants in the each of the focus groups had overwhelming positive beliefs about and attitudes toward breastfeeding. Common statements among women who had breastfeed were:

“All mothers should breastfeed” (all participants in chorus)

#### Responses from mothers’ support network

Members of mothers’ support network stated that perceived norms about the value of breastfeeding were equally high and reinforced with concrete support from a variety of personal and community sources:

“After delivery, the woman’s mother-in-law or cousins will be there to help her to breastfeed. During her antenatal period, also they approve and motivate her to breastfeed. At the time of delivery, doctors and nursing staff will motivate mothers to breastfeed.”

“Elders [grandmother and mother] give us suggestions, identify the mistakes and correct them.”

“Villagers and community people prepare energy-rich foods like edible gum laddu and coconut sweets to breastfeeding mother which help her in production of good amount of milk. This is a common practice in our culture.”

“Villagers/ community people give semolina, coconut, sugar, ghee to breastfeeding mother so that these calorie rich food help mother in production of good quality breast milk.”

“Yes, community people give ghee to the mother. They also give garden cress seeds.”

“The health worker is the first person to tell her the importance of breastfeeding.”

Enthusiasm was supported by high levels of knowledge about the benefits of breastfeeding that were apparent in each of the groups.

“The baby will have the nutritious milk as the mother takes all the nutritious food and the baby becomes healthy.”

“If we breastfeed the baby immediately after delivery, the baby will not get any diseases and the baby will have all the nutritious factors.”

“After ten minutes of my delivery the baby was cleaned and given to me and I started breastfeeding. By breastfeeding postpartum bleeding will be controlled. By breastfeeding the emotional bondage between the mother and the child increases.”

“Baby will be healthy and it will not have any diseases. Even if there is fever in baby we should not stop breastfeeding; because the breast milk itself is a medicine for baby. Whatever calcium we eat in our food it goes to baby and it becomes healthy and it will not have any illnesses.”

#### Responses from healthcare providers

Providers were another facilitator. All participants from the two provider groups were clear about advising no feeds other than breast milk, at the same time acknowledging the persistence of alternative practices.

“In spite of our advice of not to feed anything else than breast milk, people feed gripe water to babies thinking that it improves the digestive power of the baby. People also feed water to the babies.”

“Even though we tell the mothers to feed breast milk only, and not to feed anything else, they feed gripe water one month after delivery, thinking that the baby sleeps well and will have good health.”

### Specific challenges for EBF

Challenges in the form of the common practice of “top feeding”, that is feeding in addition to breastmilk, countered much of the knowledge and positive attitudes. At least half of the participants in each group reported this practice. Frequently mentioned by each group, including providers, were cow’s milk, gutti (mixture of dates, almonds, herbs, honey, gripe water (a homemade or store brought mixture of herbs and bicarbonate of soda), powdered milk, sugar water, timtim (herbal drops), and finger millet porridge. All participants agreed with the following statement:

“During summer they give little water with spoon.” Quotations from respective focus groups are as follows:

#### Responses from mothers with breastfeeding experiences

Taking colostrum for the first days after birth was reported as a problem in some communities.

“Some people give it [colostrum] and some don’t give it. They give top milk for the first 3 days.”

“Some illiterate people in spite of telling them to feed, they don’t feed colostrum. They say, ‘the lips of the baby will become black, that’s why we don’t feed it.”

“They also say the baby’s stomach gets upset [from colostrtum]. That’s why they say no.”

#### Responses from mothers’ support network

“Initially, the mother won’t be able to express milk and baby may cry in hunger; so, they put two drops of honey and sugar water to baby’s mouth. When they go home, along with breast milk they feed the baby with gutti.”

“The gutti should be fed after one and half months of delivery. Gutti is prepared by [soaking] dry dates, almond in hot water, then its coating is removed; after that they are rubbed on water washed and clean stone. Then it is mixed in the breast milk and fed to the baby. By feeding this, the babies will have good body weight and they will be fine.”

“In our area also we grind dry dates, almond and add a little Bajji powder (a mediational herb) and turmeric powder; we heat this mixture in a spoon and feed it.”

“Some people feed honey; even if we tell them not to feed.”

“People think that after delivery there will be no milk for three days. During those days, they feed honey to the baby.”

“The doctor prescribed timtim bottle to feed the children drop by drop.”

#### Responses from healthcare providers

“In the community where I work, they rub gold of Mangalasutra [wedding chain] in water and feed it to the baby. They believe that by doing so the baby will be calm and will become intelligent. I told them not to do so. But they argue, ‘We have fed this to all children in our family and for this child also we are giving it.”

“In the hospital, the nurses tell them not to feed anything else. But the attenders feed honey without the notice of the hospital staff.”

### Psychosocial difficulties

Psychosocial challenges to breastfeeding such as depression as well as substance, alcohol, and tobacco use was one of the realities frequently mentioned in focus groups. Alcohol and chewing tobacco use seemed to be common among mothers who recently delivered. Specific quotations from each focus group are as follows:

#### Responses from mothers’ support network

“If the woman delivers a male or female baby and if it is not what she was expecting; then also she may undergo depression. This is more common if she delivers female child.”

“If the woman has female children already and if again she delivers a female baby, then the mother and the family members become depressed.”

“If woman is having some personal conflicts with husband and she start feeling disgusting for her life then also she may undergo depression. This will make her to lose interest in breastfeeding.”

“Yes, it [alcohol use] is very common.”

“I have seen some mothers taking alcohol. Whether the mother delivers normally or by caesarean they take alcohol to get relief from the pain of delivery. The elders in the family like the grandmothers gave her cashew alcohol to drink when she told them that she was having pain after delivery… It does have effect on breastfeeding. After taking alcohol the mother becomes intoxicated and she will have deep and long sleep and even though the child is crying she cannot give attention towards breastfeeding her baby. She cannot wakeup for at least 24 hours from the sleep and feed her baby.”

“I have seen giving one glass of alcohol to the breastfeeding mother when she was having cough and they give one capful alcohol to the baby also when it is crying too much. Then both the mother and the baby go into sound sleep. Even though she wakes up she cannot get up and take her baby to breastfeed and as the baby is in deep sleep it cannot cry for breastfeeding.”

“They give it for one and half month or two and half month…They give it daily.”

“Even they take alcohol they don’t tell it in public.”

“Those who chew tobacco consume less food. Hence, the baby will also become weak.”

#### Responses from mothers with breastfeeding difficulties

Physical pain from C-section and breast abscesses were mentioned in the focus group of women who were not able to successfully breastfeed as challenges to breastfeeding.

“When the mother has delivered from caesarean section she is unable to sit up, at that time it is difficult for her to breastfeed.”

“If woman delivers by caesarean section, she may not be able to breastfeed the baby for one or two days. In that case, they feed the baby with cow’s milk. Some people feed goat’s milk.”

“There was a mother in our community who had a wound on her breast. Because of the wound she could not breastfeed properly. The wound was infected and she had abscess on both of her breasts which were operated in the hospital. During that time, she was unable to breastfeed the baby”

"I got infection in the breast and the breast was swollen. I was advised to get operated. I went to a nearby private hospital where I was operated. A lot of pus and blood was taken out from my breast. I got a big wound where the operation was done; for which skin grafting was done. Therefore, I could not breastfeed my baby and I am giving top feed. I gave powdered milk to my baby when I was admitted in the hospital [for abscess] and after coming to home, I am giving cow’s milk and goat’s milk. I give the top milk with spoon during the daytime and I give the milk with a bottle during the night time because the child cries if the bottle is not given for feeding.

## Discussion

Results of this study found that a facilitator of breast feeding was the knowledge about and support among all focus group participants, suggesting the success of national and global policies stressing the importance of this practice [1]. Participants spoke about the specific benefits of breastfeeding and the support offered to new mothers by family and community women. These findings mirror those of found by researchers in United Arab Emirates, Ethiopia and India [11,12,13].

In this study, a challenge to EBF was the mixed practices described about the use of colostrum which reflected older, traditional views and were contrary to providers’ recommendations [14]. More troubling was the apparent widespread use of a variety of supplemental feeding practices which seemed to exist for many concurrently with the idea of breastfeeding as best for babies. This use of additional feedings has been described by researchers in rural Zimbabwe (cooking oil, water, porridge), United Arab Emirates (anis seed drink, grippe water, tea), and Brazil (crackers, yogurt), and reflects a global challenge to the practice of EBF [15,16,17,18].

Psychosocial and physical challenges included depression over having a girl baby, alcohol use, postpartum depression, pain after delivery or Caesarean section, and breast abscesses. Most women are unable to address these very real difficulties on their own. Currently, there is no community infrastructure in India to provide specific support to breastfeeding mothers. Community-based education and support around how to deal with physical pain and breast infection issues while breastfeeding might be necessary. The establishment of a network of community health workers or breastfeeding peer counselors who can educate, support and make necessary referrals may provide a strategy to both increase EBF and also address dangerous practices such as the use of alcohol and chewing tobacco [14,19].

Two challenges to EBF identified in this study were especially troubling. The first, giving honey to newborns, is dangerous since honey is known to increase the risk of infant botulism [20]. The second is giving water from sources that may or may not be clean because infants are especially vulnerable to diarrheal diseases [21]. In place of universal condemnation of all supplemental feeding, policy makers and provider and community educators might consider strategies to address these potentially dangerous practices.

Limitations of the present study was the regional selection of participants and the self-reporting of all data. A strength was the heterogenous sample of mothers, community support members and providers.

## Conclusion

Findings from this study confirmed broad support by the target population and community members as a facilitator of breastfeeding; the challenges of the continuation of the traditional practices of supplemental feedings and very real psychosocial and physical issues limit EBF rates in rural India. Education on supplemental feeding to breastfeeding mothers and community members, as well as education on and support for behavioral and mental health factors can increase EBF rates in rural India.

## Supporting information

S1 Data (DOC)
