# Supplementary material for: Facilitators and challenges to exclusive breastfeeding in Belagavi District, Karnataka, India
Source: PLoS One. 2020 May 4;15(5):e0231755. doi: 10.1371/journal.pone.0231755 (PMC7197767; doi:10.1371/journal.pone.0231755)
Supplement: S1 Data — (DOC) [file pone.0231755.s001.doc]

**Focus group interview guide**

**Materials Needed:**

Food and Beverages

Newsprint & Markers

Masking tape

Nametags

Audiotape recorders and tape

Participant incentives

Questionnaires

Pencils

Attendance sheet

**A. Introductions and Focus Group Process (10 minutes)**

*1. Team members will have nametags on prior to participants entering the focus group venue*

*2. Give participants nametags as they arrive.*

*3. Instruct participants to select celebrity name*

*4. Have participants sign-in on attendance sheet.*

*5. Explain the purpose of the focus group session by saying:*

**“Welcome to today’s focus group. We are planning to develop mobile technology to support breastfeeding peer counselors and breastfeeding mothers. We asked you to come to today’s session because we would like to hear from you about your opinions and ideas on the technology content. You are the experts, and we can learn from you. We need your honest opinion – good and bad – about what we are trying to do. We would like you to share what you think – and what you think other members in your community might think about our project. Everything you are thinking is important to us. There are no right or wrong answers. We value your opinion. We would be very happy if you would help us to make the best technology possible.**

**Please remember to use your celebrity name and refer to others with their celebrity names. Also, try not to use any friends’ names or specific locations. But if you do, we will delete them from the audio recording.**

*As a reminder we are going to turn on the tape recorder now.*

*Is everyone ok with that? TURN TAPE ON: and announce*

“This is focus group # 01 on 07/03/2018 for the “BEST4Baby”

*1. Ask the participants to introduce themselves by saying their celebrity name and what is your favorite color.*

*2. All members of investigative team introduce themselves.*

**“Now, we would like to develop some group rules so we can learn as much as possible from each other.”**

**B. Group Rules (10 minutes)**

1. *Develop group rules to protect participants’ confidentiality. Offer examples of group rules if participants are stuck:*

Be respectful

Be honest

Maintain confidentiality

One person speaks at a time

Listen to others

No put downs or insults

Turn off all cell phones

**“Even though we will be talking about breastfeeding and technology development today, we do not require you to talk about your personal experiences with breastfeeding if you do not feel comfortable. You can instead share what you think or others’ experiences. However, please do not use any specific names or identifiable information of others for protecting their privacy. Is that clear to everyone?”**

**BREAST FEEDING EDUCATION SUPPORT TOOL for BABIES**

**Focus Group Discussion guide for Members of breastfeeding mother’s support network (e.g., mother, mothers-in law, etc.)**

**Discussion (40 Minutes)**

**“We are working to develop a mobile technology to support breastfeeding peer counselors and breastfeeding mothers in India. We want to know what you think and what you know about breastfeeding practices. Your inputs will certainly help develop feasible and effective technology to support breastfeeding. We have questions we prepared but we will let our group guide our own discussion. Let’s start with some initial questions about breastfeeding experiences.”**

1. ***Breastfeeding practice and support mechanism (20 minutes)***
2. Tell us about breast feeding experiences around you.

**Moderator:** What do you and the community at your respective places say about breast feeding? Whether to breastfeed or not?

**All Participants (In chorus):** All mothers should breastfeed..

1. Do you think breast feeding is important for the baby?

**And**

**Breastfeeding attitude**

1. Why? What are the benefits?

**Moderator:** What are the benefits of breastfeeding?

**Participant 2:** Babies will improve properly if breast fed and the milk secretion also improves.

**Moderator:** What do you mean “properly”?

**Participant 2:** The baby will have the nutritious milk as the mother takes all the nutritious food and the baby becomes healthy.

**Participant 1:** Baby gets nutritious milk.

**Moderator:** What happens if baby gets nutritious milk?

**Participant 1:** Baby gets immunity.

**Participant 2:** Baby doesn’t get any disease. Baby will not become mentally retarded.

**Participant 10:** Mother’s milk is better than other types of milk. My grandchildren all had breast milk for 3 years. 2 years, 2 and half years and 3 years. Just recently they have stopped breastfeeding.

**(Other participants also agreed to this)**

1. If you think breastfeeding is not good, what would be the reasons?

**Moderator:** Are there any women who do not breastfeed?

**Participant 2:** Yes, there are.

**Participant 1:** Yes, there are.

**Moderator:** What would be the reason?

**Participant 1:** If there is no milk at all in the breast.

**Participant 2:** Even if the breast is pressed, there is no milk. Therefore rice porridge and top milk is fed to the baby.

**Participant 4:** It has happened to my daughter in law herself. Caesarean was done. After caesarean there was no breast milk. Even then, we made the baby to suck and now there is sufficient breast milk. Even if caesarean is done, there will be no shortage of breast milk.

**Participant 2:** Will not become less. It will be there. If there is mental tension, if only female children are born.

**Participant 5:** If only male babies are born…..

**Participant 2:** Some mothers do not eat proper nutritious food**.**

1. How long does the mother need to breast feed exclusively and the total duration?

**Moderator:** Now, we are discussing about two types of breastfeeding; one is exclusively breastfeeding; and the other is breastfeeding with initiation of top feeding. For how long should the exclusive breastfeeding is to be done?

**All Participants:** 6 months.

**Moderator:** For how long should be the total duration of breastfeeding?

**Participant 1, 4 and 5:** One and half years.

**Participant 2:** 3 years

**Participant 10:** 3 years

**Participant 7:** Why only for 6 months; it should be fed till 9 months. I have breast fed for 3 years.

**Participant 8:** I have breast fed for 3 years.

**Participant 6:** At least for the duration of one and half years.

**Majority participants:** The total duration of breastfeeding should be at least one and half years.

**Breastfeeding social norm**

1. Do all of the mothers in the community breastfeed?

**AND**

1. Who approves breastfeeding around you?

**Moderator:** What is the opinion of community members about breastfeeding? Do they say that mothers should breastfeed or not to breast feed?

**All Participants (In chorus):** All the mothers should breast feed their children.

**Moderator:** Does the mother have to take permission from the family members to breast feed?

**All Participants:** No, no. No need to take permission. As a mother she breast feeds her baby.

**Moderator:** Suppose there are 4 to 5 members in the family, and a young mother delivers, does anybody in the family tell her to breastfeed the baby? Or she herself does it?

**(**Some participants said that mother herself breastfeeds the child on her own; and some participants said that mother or mother in law tell and help her in breastfeeding the baby.)

**Participant 10:** One has to take out the breast, hold it and show her how to feed the baby.

**Participant 7:** The breast has to be massaged if there is swelling of the breast. And the baby should be breast fed.

**Moderator:** Who does all these things?

**All Participants:** We only do it.

**Participant 7:** The one who conducts the delivery.

**Participant 1:** The elders in the family, the mother or mother in law; they have to do it.

**Participant 3:** After the delivery, within half an hour the mother has to breast feed her baby.

**Moderator:** Who does this? Who takes the care about the breast feeding?

**Participant 5:** We only have to do it. We only have to identify the mistakes and correct them.

**Participant 6:** Before we tell her, she also has to take care. We are also supposed to tell her.

**Participant 7:** If she doesn’t understand, we have to try to make her understand, and see that she breastfeeds her baby.

**Participant 8:** Yes we need to do that.

**Participant 9:** We need to explain her; we need to wash her breast and make her to feed the baby.

**Participant 10:** The baby should be breast fed. At the first time, after the first delivery she will not be knowing how to breastfeed the baby; and we only have to tell her and make her understand……

**Moderator:** Who will tell her? Only the family members or anybody else?

**All Participants:** No, no no…. Only we do it.

**Participant 3:** My younger sister was admitted for delivery. I only helped her in breastfeeding.

**Participant 6:** I tell this in my house; and also to the women in our village.

**Participant 7:** I also tell all the women in my village. I have done about 500 deliveries in my village. If there is swelling of the breast I used to massage and make the baby to breast feed within half an hour.

1. Who disapproves breastfeeding around you?

AND

1. What problems did you encounter in breastfeeding?

**Moderator:** Are you aware of any problems in breastfeeding? What are all the difficulties in breastfeeding?

**Participant 6:** If there is swelling in the breast, we need to massage it and clean with warm water and feed the baby.

**Participant 7:** The baby may have soreness of the mouth. We express the breast milk and throw it away. After everything becomes okay, she will start breastfeeding.

**Participant 2:** If the mother doesn’t breast feed the baby, she will have swelling in her breast.

**Participant 3:** The mother should clean the breasts before breastfeeding.

**Participant 2:** The breast may not be having nipple. The nipple may not be clean. We need to clean it and give it into the mouth of the baby.

**Participant 5:** Sometimes the baby may not have burp after feeding. In that case we take the baby on the solder and rub its back. Then the baby will be fine after feeding.

**Participant 6:** I am of the same opinion. The mother should take her baby to her chest. She should wash and massage her breasts. The baby cannot suck the breast if there is swelling of the breast.

**Participant 7:** If the milk is blocked in the breast the baby cannot suck it. In such cases if the breast is massaged and the milk is taken out the swollen nipple will reduce in size and it can be inserted in the baby’s mouth and it will be fine.

**Participant 8:** There may be difficulty in breast feeding because there may not be milk secretion in the breast. The baby may not suck the breast, if the nipples are swollen. In that case we need to massage the breast and feed the baby.

**Participant 9:** I have done many deliveries. The breast milk started coming from third day after delivery and started breastfeeding from then on.

**Participant 10:** If there is more milk in the breast it is difficult for the baby to swallow it. The mother has to sit and hold the baby properly and breastfeed. Otherwise it will be difficult for the babies to breathe.

**Participant 1:** If the breasts are too big, there will be difficulty in breast feeding. Then the milk has to be expressed and fed to the baby.

**Participant 2:** Sometimes for the first delivery there may be tingling sensation while breastfeeding and it is difficult for the mother to breast feed the baby.

**Moderator:** Any other problem you have seen? And not only you, have you heard of anybody else seen any problem about breastfeeding?

**Participant 10:** When the mother is feeding her baby for the first time, the baby may not be knowing how to suck the nipple properly, then there will be cracks on the nipples.

**Participant 2:** The baby may not be having enough strength to suck the breast.

**Participant 10:** When the baby holds the nipple by mouth and pulls it repeatedly it becomes sore.

**Participant 1:** When there is wound on the nipple; we need to wash it with soap and then we need to express the milk and feed it to the baby.

1. What do you and people around you think about prelacteal feeds?

**Moderator:** After delivery, apart from breast milk do the people feed anything else to the newborn baby? Tell me from number 1.

**Participant 1:** No, no nothing like that.

**Participant 2:** Some people feed. But it should not be done. They feed honey and sugar water.

**Participant 3:** We have not fed anything.

**Participant 4:** Glucose water is given in the hospitals.

**Participant 5:** We have not fed anything.

**Participant 6:** We have fed only breast milk.

**Participant 7:** We have not fed anything.

**Participant 8:** We have fed only breast milk within half an hour.

**Participant 9:** We have fed only breast milk.

**Participant 10:** We have fed only breast milk.

**Moderator:** According to you whether the prelacteal feeds should be given or not?

**Participant 1:** Only breast milk should be fed.

**Participant 2:** Only breast milk should be fed. The honey should not be fed.

**Participant 3:** Only breast milk should be fed.

**Participant 4:** Nothing else but only breast milk should be fed.

**Participant 5:** Only breast milk should be fed.

**Participant 6:** Only breast milk should be fed.

**Participant 7:** Only breast milk should be fed.

**Participant 8:** Only breast milk should be fed.

**Participant 9:** Only breast milk should be fed.

**Participant 10:** Only breast milk should be fed.

**Moderator:** According to you what happens if top feed is fed to the baby? We start from number 1.

**Participant 1:** Children do not improve.

**Participant 2:** Baby’s stomach gets upset.

**Participant 3:** We do not feed anything else to the baby.

**Participant 1:** The people in the community feed.

**Participant 2:** They don’t feed now. Earlier they used to feed but now they have stopped.

**Participant 3:** Some people feed; some people feed honey; even if we tell them not to feed**.**

**Participant 4:** People think that after delivery there will be no milk for three days. During those days they feed honey to the baby.

**Participant 5:** Yes, honey is fed.

**Participant 6:** People feed honey. But we don’t.

**Participant 7:** In the hospital, the nurses tell them not to feed anything else. But the attenders feed honey without the notice of the hospital staff. And the people in community also feed.

**Participant 8:** Yes, they feed. But if we see it, we tell them not to feed.

**Participant 9:** Yes, they feed.

**Participant 10:** Yes, they feed.

**Moderator:** So, all ten of you say that the people around you give prelacteal feeds to the baby’s?

**All Participants (In chorus):** Yes they give.

1. What do you and people around you think about colostrum?

**Participant 1:** The colostrum should be fed. It has to be fed for three days. And the people in our community feed it.

**Participant 2:** At our place also they feed it. There are vitamin A and B in it. They are available to the baby. Whatever the nutrition the mother has had during the 9 months of pregnancy, it is all there in the colostrum.

**Moderator:** Is it the opinion of people in your village?

**Participant 2:** Yes.

**Participant 3:** Some people give it and some don’t give it. They give top milk for the first 3 days.

**Participant 4:** Within half an hour after birth the colostrum should be given. When we go to do a deliveries we tell them to breastfeed within half an hour after delivery

**Participant 5:** All people feed the colostrum at our village.

**Participant 6:** All people feed the colostrum at our village.

**Participant 6:** If the colostrum is fed the children become healthy.

**Participant 7:** It should be fed so the baby’s become healthy. The baby’s health improves. Whenever we go to conduct delivery we tell them to feed it and we make the mother to feed the colostrum and then only we come back.

**Participant 8:** It should be fed. And we also tell the people around us to feed the colostrum.

**Moderator:** You tell them to feed and you come back. Whether they feed it or not?

**Participant 8:** Yes they feed.

**Participant 9:** They don’t have any objection for it and they say that it should be fed. It improves the health of the baby

**Participant 10:** They feed in our village. There is more immune power in the colostrum. The babies will not have any illness.

**Moderator:** Opinion of the majority people is to feed the colostrum and in the community also…….

**Participant 2, 7:** They also say that it should be fed.

**Moderator:** Whether majority of people say to feed it or some people say to feed it and some do not say?

**Participant 2, 7:** They say to feed it.

**Participant 3:** Majority of people feed it. We have seen in the government hospital, some illiterate people in spite of telling them to feed, they don’t feed colostrum.

**Moderator:** Why they don’t feed?

**Participant 3:** They say “The lips of the baby will become black, that’s why we don’t feed it”. And they also say the breast becomes painful; therefore they don’t feed. They take the child to other breastfeeding mothers and breast feed it. But they don’t feed colostrum of its mother.

**Participant 2:** They say that the lips of the baby become black and they also say the baby’s stomach gets upset. That’s why they say no.

**Participant 2:** They say that they will lose their figure. They say the chest loses its shape.

**All Participants: (Laugh)**

1. What do you and people around you think about exclusive breastfeeding?

**Participant 1:** People in the community tell the same and ask the mother to breast feed for 6 months.

**Participant 2:** They say that it is good to breast feed for 6 months.

**Participant 3:** There are working women who feed their babies with top milk.

**Participant 4:** In our village nobody gives top milk and they give breast milk only. And people say it is good if breastfeed is given.

**Participant 5:** Breastfeeding should be done. The mothers need to go for work in the field therefore people say to give goat milk and cow milk.

**Participant 6:** All mothers in our village feed breast milk only. But some mothers go to field for work. It is difficult for them to breastfeed.

**Participant 7:** In our village, mothers come back from work to house and breastfeed oththe babies or they give top milk.

**Participant 8:** They feed breast milk as much as possible otherwise some working mothers feed top milk.

**Participant 9:** In our area also only breast milk is fed. People say only breast milk should be fed.

**Participant 10:** For working women there is leave for 3 months and they stay in the house. Only if there is any problem it is difficult for them to feed the baby.

1. What do you and people around you think of gripewater /gutti /any medicines during the first six months?

**Participant 7:** Yes. They feed. If the mother’s breast milk is insufficient they feed top milk.

**Participant 2:** If there is engorgement of breast they give top feed.

**Moderator:** Why the breast engorgement occurs?

**Participant 2:** If the breast milk is not fed, there will be swelling.

**Participant 7:** If the breast milk is not fed, there will be swelling.

**Participant 2:** If the mother doesn’t breastfeed early she gets swelling of the breast. It becomes painful. Later, even though she wants to breastfeed it is difficult.

**Participant 1:** If it is difficult for the mother we need to massage…….

**Participant 2:** If the mother is not well.

**Participant 7:** If the mother is not well and weak she stops breast feeding.

**All Participants:** After 6 months they give top feed.

**Participant 10:** Yes. They give top feed.

**Participant 9:** After 6 months they give little water.

**Participant 9:** During summer they give little water with spoon. **(All Participants expressed agreement with participant 9)**

**Moderator:** Then what about gripe water and gutti, do they feed?

**All Participants:** Yes. They feed gutti and gripe water.

**Participant 10:** Yes. They feed gutti and gripe water..

**Moderator:** What do they feed? Gripe water or gutti?

**Participant 10:** They feed gripe water

**Participant 9:** Yes. They feed the same at our place also.

**Moderator:** Do they feed these things to all children within 6 months?

**Participant 9:** Yes.

**Participant 8:** Till 6 moths nothing else fed. In the area, surrounding my house nobody feeds anything else.

**Participant 7:** People feed such things.

**Participant 6:** The same thing, gripe water is fed. They grind dry dates and almonds and feed them.

**Participant 7:** In our area also we grind dry dates, almond and add a little Bajji powder (a kind of mediational herb) and turmeric powder; we heat this mixture in a spoon and feed it.

**Participant 5:** They feed those things at our place also.

**Participant 4:** At our place also we grind and heat the gutti and feed it.

**Participant 3: (Nods yes)**

**Participant 2:** At our place also dates, almond and valshingi (A kind of herb) gutti articles are available. They mix all these things, grind them and feed them. We don’t feed grip water at our place.

**Participant 1:** We too feed the same.

**Moderator:** At how of your villages grip water is fed. Can please raise your hands.

**6 Participants:** Raised their hands.

**Moderator:** One, two, three, four, five and six. At six of your villages gripe water is fed. In the rest of the villages do they feed gutti? And in all of the ten villages they feed gutti?

**All Participants (In chorus) :** Yes.

**Participant 1:** The present generation do not feed gutti. They don’t like it.

**Moderator:** What the present generation feed?

**Participant 1:** They feed readymade feeds.

**Participant 2:** They feed what children doctors prescribe.

**Participant 7:** Doctor prescribe tim, tim bottle to feed the children drop by drop. (Number 2 laughs agrees that in her area also they use the tim tim bottle)

**Participant 1:** While preparing gutti some people don’t wash the stone cleanly on which it is grinded (She mean to say that it may cause infection to the baby).

**Participant 7:** Young neonates are given medicines.

**Moderator:** As others told prescribing like tonic, gripe water or gutti. Now you all told that people feed gripe water, gutti, Tim Tim bottle these things to the babies. Why do they feed these things? Let us start it from number 1.

**Participant 1:** For the baby to become cute.

**Participant 2:** For the baby to sleep well. It also reduces pain abdomen of the baby. If there is any problem to overcome it.

**Participant 3:** For the improvement in the growth of the baby.

**Participant 4:** Growth of the children.

**Participant 7:** To improve the growth of the baby. The baby will be calm and will not cry too much.

**Moderator:** Are there any other reasons?

**Participant 2:** To enhance the apatite of the baby.

**Participant 10:** If mother eats something else the baby may have pain in the abdomen. If the mother drinks more water the baby will be in trouble.

**Participant 4:** If the mother drinks more water, she will have more milk. We should not give anything else to the baby accept breast milk.

1. Anything else you would like to add that you think is important?

-

**Breastfeeding perceived control**

1. What makes breastfeeding easier?

**Participant 10:** The mother should initiate breastfeeding early.

**Participant 9:** The mother need to apply coconut oil and massage her breast and then feed the baby.

**Moderator:** By doing so, does the breastfeeding becomes easier?

**Participant 9:** Yes.

**Participant 8:** From the sixth month during pregnancy, while taking bath, the breast should be massaged. The nipple should be massaged and made correct. By doing so she will have good breast milk after delivery.

**Participant 7:** Some Mothers don’t know how to breastfeed. For them we need to explain about cleaning the breast and how to put it into the baby’s mouth for feeding. If we make all this the baby continues the breastfeed on its own.

**Moderator:** To do that one of you have to be there.

**Participant 7:** Yes.

**Participant 6:** If the babies are covered by saree while breastfeeding they will be fine. **Moderator:** Why?

**Participant 6:** If not covered the baby will have evils eye on it.

**Participant 5:** if the mother takes vegetables and nutritious food in her diet, she will have good breast milk.

**Participant 4:** Sometimes the breasts become engorged. Even though there is milk in the breast it may not come out.

**Moderator:** How do you make it easy?

**Participant 4:** If we express the breast milk and feed it to baby, the milk starts coming.

**Moderator:** With what you take out the breast milk?

**Participant 4:** In the hospital they had given us a pump to do this. The breasts were filled with milk and had become large in size. But the milk was not coming at all. After using the pump the milk started coming. The people in the community should breastfeed compulsorily. Even though the breast milk doesn’t come for short time, we need to breast feed the baby continuously. Then the breast milk will start coming.

**Participant 3:** If the mother undergoes caesarean then it will be difficult for her to breastfeed. Someone should help her in breastfeeding.

**Participant 2:** From the third month of pregnancy the mother should clean and massage the breasts and the nipple. Then it will become easy for her to breastfeed after delivery.

**Participant 1:** If the nipple is made correct, the breast milk will come automatically for the baby. If the mother’s breast milk is to be good, she has to take vegetables and cow milk.

1. What makes breastfeeding harder?

**Participant 1:** For the first delivery it is difficult for mother to get up and breast feed.

**Moderator:** For which mother it is difficult to breast feed?

**Participant 1:** For the mother who undergone caesarean.

**Participant 2:** If the BP is high at the time of delivery.

**Participant 1:** If the mother is having raised BP we send her to the hospital immediately.

**Participant 7:** If the mother is suffering from diabetes and BP…etc. And if she doesn’t take nutritious food she will have insufficient milk. That makes difficult for her to breastfeed.

**Participant 3:** Even if the breast milk is adequate baby may feed less, then the mother will have difficulty.

**Participant 2:** Because of mental tension the mother may not have breast milk.

**Participant 7:** Due to tension the mother will not have breast milk.

**All Participants: (Laughs)**

**Moderator:** Why all of laughing, when she said tension?

**Participant 1:** It is common for the mother to have some or the other tension in the family.

**Participant 4:** If there is breast engorgement due to less feeding by baby.

**Participant 1:** The breast milk gets spoiled if it is not fed to the baby. Therefore while bathing the mother we pore water and massage the breast and express the milk.

1. What does your community think of roles played by maternal depression on breastfeeding?

**Participant 1:** The milk doesn’t become less.

**Participant 2:** The milk will become less.

**Moderator:** If there is any mental tension, what we call as depression, there may not be interest in breast feeding and if she doesn’t have enough food.

**Participant 10:** There may be some problems in the husband’s family. Because of tension she may not have proper food.

**Moderator:** What happens? What is its effect on breast feeding?

**Participant 10:** If there is less milk it will be a problem for the baby.

**Moderator:** Has anybody seen mothers having mental depression?

**Participant 7:** No, no. I don’t know at all.

**Moderator:** Have you not seen in the community around you? Can you not understand what its effect on breastfeeding?

**Participant 7:** We are unable to recognise it whether it does effect on breastfeeding or not.

**(All the participants laugh)** We cannot make out whether the breastfeeding has become less due to that.

1. What does your community think of roles played by maternal smoking on breastfeeding?

**Moderator:** In your community do mothers use betel nut, leaves and tobacco?

**All Participants (In chorus):** Yes.

**Moderator:** Do breast feeding mothers use tobacco?

**All Participants (In chorus):** Yes.

**Participant 6:** To get the relief of toothache they hold tobacco to the tooth.

**Participant 2:** Some eat Star (Mixture of betel nuts, lime and tobacco).

**Participant 1:** I have not seen anybody.

1. What does your community think of roles played by maternal drinking on breastfeeding?

**Participant 10:** Yes. They take.

**Participant 2:** Yes. They take.

**Participant 7:** In some villages they take.

**Moderator:** Do even breast feeding mothers take alcohol?

**Participant 7:** Yes, they also take.

**Participant 10:** Rarely some one or two women take.

**Participant 2:** Yes, they take Brandy.

**Moderator:** Some people take brandy thinking that it is good if taken after delivery. Like that do they take in your village?

**Participant 1, 2:** Yes, they take.

**Participant 3, 4:** No.

**Participant 5:** Yes, they take.

**Participant 6:** No.

**Participant 7:** They take but nobody tells that they take.

**Participant 2:** Nobody accepts that she takes alcohol.

**Participant 1:** Yes, yes. No woman says that she drinks alcohol. We have noticed it. In that way that’s why we are telling you about it. Nobody in our family takes it. Few women in the village take it.

**Participant 2:** Even they take alcohol they don’t tell it in public.

**Moderator:** Due to drinking alcohol is there any effect on the breast feeding?

**All Participants:** Yes. It does have effect on breast feeding.

**Participant 7:** There will be problem to the baby. If they drink alcohol and breastfeed the baby, the alcohol will pass on to the baby through the breast milk.

**Participant 1:** The baby may have vomiting and diarrhoea.

1. What does your community think of roles played by maternal drug use on breastfeeding?

**Moderator:** Apart from alcohol, do they take narcotics like Ganja (Drug)?

(None of the participants were willing to answer)

1. What does your community think of roles played by maternal anaemia on breastfeeding?

**Participant 1, 2 :** Yes. It has effect.

**Participant 3:** Yes, it will have effect.

**Moderator:** Does everybody say that it will have effect on breastfeeding.

**All Participants:** Yes, it will have effect.

**Moderator:** What effect it will have on the baby?

**Participant 1:** The baby will not improve. If there is not sufficient milk the baby will not improve. Baby will not be calm, it will cry and it will be impatient

**Participant 2:** Baby doesn’t get sufficient milk.

1. What does your community think of roles played by maternal overweight or obesity on breastfeeding?

**Majority Participants:** Yes it will have effect.

**Participant 10:** Some women, even though they are obese, they will have less breast milk.

**Participant 9:** They do not breastfeed properly; they feed more top milk to the baby. They don’t have interest in breastfeeding. We village people breastfeed

**Participant 8:** Those women who are obese will be anaemic. Therefore there will be effect on breastfeeding.

**Participant 7: (**Starts laughing) We have seen obese women breastfeeding. But those women will have less milk. Because they will be having menstrual problem. And also they eat less food because they think that if they eat more they will become more obese. Because of that they will have less breast milk.

**Participant 6:** They avoid eating rice, because they think that, if they eat rice their weight will increase. And that will lead to less breast milk in them.

1. What does your community think of roles played by intimate partner violence on breastfeeding?

**Moderator:** Some husband and wife may have quarrel with each other or mother in law; are there any such couples in your village?

**All Participants:** Yes, there are.

**Moderator:** Has this any effect on breastfeeding of the mother? Have you seen any such breastfeeding mother?

**Participant 4:** Regarding this, you only have to tell us. So that we can convey it to others.

**Participant 1:** Yes, it does have effect on breastfeeding. Due to violence by the husband the wife may not have interest in taking adequate food, which leads to less breast milk secretion.

**Participant 7:** Don’t ask me this question. (She was hesitant to answer)

**Moderator:** Why?

**Participant 7:** I have lot of experience in this matter.

**Moderator:** According to your opinion, does this have any effect on breastfeeding?

**Participant 7:** Yes, it does have effect.

**Participant 3:** It does have bad effect on breastfeeding.

***B.*** ***Acceptability of breastfeeding peer counselors (10 minutes)***

1. Where do you get information about breast feeding?

**Question not asked.**

1. If you have any breast feeding problem whom do you approach?

**Participant 2:** Doctors.

**Participant 1:** They go to a doctor or hospital.

**Participant 3:** To a doctor or hospital.

**Participant 6:** To a doctor.

**Participant 4:** To a doctor.

1. Are you aware of any programmes by the government to promote Breast feeding practices in the country?

**Majority Participants:** No.

**Participant 4:** Government people come to us to tell about this.

**Moderator:** What do they tell when they come to you?

**Participant 4:** They come to us to tell to breastfeed. To keep the breast clean etc.

**Moderator:** Who comes?

**Participant 4:** The staff from government hospital come, ASHAs also tell us about this.

**Participant 2:** There are posters displayed in hospitals. We have read them when we go for delivery

**Moderator:** Which are the programmes can you tell me?

**Participant 2:** There are no programmes yet. Doctors from medical college come here and conduct meetings. They tell us in the meeting.

1. Is there any support from the women from the village/community?

**All Participants (In chorus):** Yes.

1. Is she trained in giving breast feeding support and help?

**Participant 2:** There is nobody who has taken training.

**Participant 1:** If there is any such problems we only go.

**Participant 7:** If there is any problem they call us. If the baby doesn’t breastfeed.

**Participant 1:** I have been trained.

**Moderator:** Trained in what?

**Participant 1:** I have been trained in conduction delivery

**Moderator:** Are you trained in breastfeeding? About how to hold the baby, about how many days to breastfeed the baby and about how to give support to the mother….

**Participant 1:** I have been told about these.

**Moderator:** There is training about breastfeeding. Is there anybody trained in this aspect?

**All Participant (In chorus) :** No, no.

**Breastfeeding peer attitude**

1. Do you think that a person (Peer Counsellor) from your community/village, if trained will be helpful to solve the breast feeding problems?

**All Participants:** It will be useful.

**Moderator:** Such a person should be there or not?

**All Participants:** Should be there.

1. Do you accept receiving Breast feeding counselling and advice from such a person?

**Moderator:** Is it useful if a trained person about all this is appointed and made available in your village?

**All Participants (In chorus):** Yes, it will be useful.

**Moderator:** Do people in your village accept such persons?

**All Participants:** Yes

**Moderator:** Do they allow such person to work?

**All Participants (In chorus):** Yes, they will allow her.

**Moderator:** She will be from your village only. Do they object saying, “Why she has come?” **All Participants (In chorus):** No, no. They don’t object.

**Participant 4:** Earlier, there were no ASHAs in the villages. Now they are there and people have accepted them. Likewise people will accept her also.

**Moderator:** Is there anybody who do not accept?

**All Participants (In chorus):** No, no.

1. What should be the qualifications of Peer Counsellor?

**Participant 2:** She should be knowing about the problem being faced by the breastfeeding mother and she should be able to explain to the breastfeeding women about the problems. She should also have the feelings like the breastfeeding mother has.

**Moderator:** What qualities the mother should have.

**Participant 10:** She should understand the problems of the breastfeeding mothers and should be able to solve the problems like she should treat her as her daughter. First, she herself should be having good qualities.

**Moderator:** What do you mean by good qualities?

**Participant 4:** She should not eat tobacco and she should not have any bad habits.

**Moderator:** Whether she should have experience or not?

**All Participants (In chorus):** Yes, she should have experience.

**Moderator:** What type of experience she should have?

**Participant 4:** The people like you should guide her about what she should do. We don’t know anything about it, we are doing what is there in the villages. If you give us training then, we will do.

**Moderator:** I am asking you, what experience she should have first.

**Participant 2:** She should have a daughter or a daughter in law delivered in her family. She should have experience of telling to them about breastfeeding.

**Breastfeeding peer perceived control**

1. What would be the benefit to have Peer Counsellor?

**Question not asked.**

1. What would be the barriers/problems to have Peer Counsellor?

**Moderator:** If the peer counsellor comes into your village do you help her?

**All Participants (In chorus):** Yes we do.

**Moderator:** If they face any problem what should they do? Whom they should approach? Do the people in the village help them?

**Majority Participants:** People will help them.

**Breastfeeding peer social norm**

1. Who approves the presence of Peer Counsellor?

**Not asked the question.**

1. Who disapproves the presence of Peer Counsellor?

Already answered as nobody in the community objects the presence of peer counsellor.

1. **Program to train breastfeeding peer counselors (10 minutes)**
2. How does the health worker help you in breast feeding activities?

**Moderator:** In your village ASHA workers and ANMs come to your houses. Do they council about breastfeeding?

**All Participants (In Chorus):** Yes.

1. Does the health worker visit and advise you about Breast feeding before delivery?

**Moderator:** Do ASHA worker and ANMs tell about breastfeeding when they visit a woman during antenatal period?

**Participant 1 and 2:** Yes.

**Participant 3:** Yes, they tell.

**Participant 4:** They have not told much about breastfeeding.

**Participant 5:** Yes, they tell.

**Participant 6:** Yes, they tell.

**Participant 7:** They tell about breastfeeding after the delivery.

**Moderator:** Not after delivery. I am asking you whether they tell about breastfeeding before delivery.

**Participant 7:** No.

**Participant 8:** Yes, they tell.

**Participant 9 and 10:** Yes, they tell.

1. What does the health worker talk about breastfeeding antenatally?

**Not asked the question.**

1. What happens at birth? Is she present at birth and help you with early starting of BREAST FEEDING?

**Moderator:** After delivery how early the Health staff tell the mothers to breastfeed her baby?

**All Participants:** They tell the mother to breastfeed her baby immediately after delivery.

**Participant 10:** ASHA will be there at the time of delivery and she tells the mother about breastfeeding.

**Moderator:** Does she make the mother to breast feed or only she tells her to breastfeed?

**Participant 10:** She makes the mother to breast feed her baby.

**Participant 8:** She holds the baby and helps her to breastfeed it.

1. How frequently does she visit you after birth of the baby?

**Participant 1:** Three to four times.

**Participant 2:** Once

**Participant 3:** Three times

**Participant 4:** Three times.

**Participant 5:** Three times.

**Participant 6:** Four to five times.

**Participant 7:** Four to five times.

**Participant 8:** Minimum two to three times.

**Participant 9:** Minimum three times.

**Participant 10:** They do come.

1. What is the purpose of visit?

**Moderator:** What they do when they come to visit the mother after delivery?

**Participant 1:** They give information. They tell about breast feeding.

**Moderator:** If there is any problem do they help?

**Participant 1:** Yes, they do help.

**Participant 7:** If there is any problem they hold the baby and make it hold the nipple by its mouth.

1. How frequently does she assess your breast feeding practices?

**Not asked the question.**

1. How many mothers does the health worker visit per day/per month?

**Not asked the question.**

1. What are the common breast feeding problems you observe in your community?

**Already answered in previous questions.**

1. Do you think that peer counsellor who is from the same community would help and support mothers?

**Already answered in previous questions.**

1. What do you think would be the barriers for Peer Counsellors in the community?

**Already answered in previous questions.**

1. Will there be future opportunities for these Peer Counsellors to continue in the community?

**Not asked the question.**

1. What should be criteria for acceptance of such counsellors by you and the community?

**Already answered in previous questions.**

1. How do you identify and recruit such counsellors?

**Moderator:** Are there such women in your village?

**Participant 2:** They should have experience; she should take care like a mother to the breastfeeding woman.

**Moderator:** What other qualities she should have?

**Participant 2:** She should tell everything without any hesitation.

**Moderator:** Are such woman available in your village?

**Participant 1 and 2:** Yes, they are available.

**Moderator:** Can you identify such women?

**Except Participant number 10, all participants:** Yes

**Participant 4:** ASHA workers know everything about such women, we don’t know much about it. They go into the villages for their work and they know who is what, they know who is available, rather than asking us, you better ask ASHA worker.

**Moderator:** I am asking about community people?

**Participant 4:** Okay we also tell them about it. But to find out such women only you need to ask ASHA workers.

**Moderator:** You are also prominent elderly persons in your village. You also go into the village. Can you not find such persons?

**All Participants (In chorus):** Yes, we can.

**F. Wrap up**

1. *Ask participants*

**“How did you feel about participating in this session? What was easy? What was hard?”**

1. *Summarize and thank participants by saying:*

**“We appreciate all of your great ideas. You have been a big help, and we want to thank you very much for all the information you have shared with us today. We know that your ideas will help to make this a successful program and technology. Thank you once again for your participation – we really appreciate you!”**

**Thank you all.**

**Focus group interview guide**

**Materials Needed:**

Food and Beverages

Newsprint & Markers

Masking tape

Nametags

Audiotape recorders and tape

Participant incentives

Questionnaires

Pencils

Attendance sheet

**A. Introductions and Focus Group Process (10 minutes)**

*1. Team members will have nametags on prior to participants entering the focus group venue*

*2. Give participants nametags as they arrive.*

*3. Instruct participants to select celebrity name*

*4. Have participants sign-in on attendance sheet.*

*5. Explain the purpose of the focus group session by saying:*

**“Welcome to today’s focus group. We are planning to develop mobile technology to support breastfeeding peer counselors and breastfeeding mothers. We asked you to come to today’s session because we would like to hear from you about your opinions and ideas on the technology content. You are the experts, and we can learn from you. We need your honest opinion – good and bad – about what we are trying to do. We would like you to share what you think – and what you think other members in your community might think about our project. Everything you are thinking is important to us. There are no right or wrong answers. We value your opinion. We would be very happy if you would help us to make the best technology possible.**

**Please remember to use your celebrity name and refer to others with their celebrity names. Also, try not to use any friends’ names or specific locations. But if you do, we will delete them from the audio recording.**

*As a reminder we are going to turn on the tape recorder now.*

*Is everyone ok with that? TURN TAPE ON: and announce*

“This is focus group # 2 on 08 / 03 / 2018 for the “BEST4Baby”

*1. Ask the participants to introduce themselves by saying their celebrity name and what is your favorite color.*

*2. All members of investigative team introduce themselves.*

**“Now, we would like to develop some group rules so we can learn as much as possible from each other.”**

**B. Group Rules (10 minutes)**

1. *Develop group rules to protect participants’ confidentiality. Offer examples of group rules if participants are stuck:*

Be respectful

Be honest

Maintain confidentiality

One person speaks at a time

Listen to others

No put downs or insults

Turn off all cell phones

**“Even though we will be talking about breastfeeding and technology development today, we do not require you to talk about your personal experiences with breastfeeding if you do not feel comfortable. You can instead share what you think or others’ experiences. However, please do not use any specific names or identifiable information of others for protecting their privacy. Is that clear to everyone?”**

**BREAST FEEDING EDUCATION SUPPORT TOOL for BABIES**

**Focus Group Discussion guide for Facility- and community-based Health Care providers**

**Discussion (40 Minutes)**

**“We are working to develop a mobile technology to support breastfeeding peer counselors and breastfeeding mothers in India. We want to know what you think and what you know about breastfeeding practices. Your inputs will certainly help develop feasible and effective technology to support breastfeeding. We have questions we prepared but we will let our group guide our own discussion. Let’s start with some initial questions about breastfeeding experiences.”**

**A. *Breastfeeding practice and support mechanism (20 minutes)***

1. Do all of the mothers in your community breastfeed?

**All Participants (In chorus):** Yes, all the mothers in our community breastfeed their children.  **(All nodded their head while saying “yes”)**

**Breastfeeding attitude**

1. Are there any circumstances that might arise that you can think of where it might be okay for you to encourage a mother not to breast?

**All participants (In chorus):** Yes, there are some circumstances where women do not breastfeed their children.

**Participant 4:** If mother has AIDS (Acquired Immune Deficiency Syndrome) she should not feed her child.

**Participant 6:** If she has breast abscess.

**Participant 5:** Nothing like that, they can feed their children in any circumstance.

**Participant 1:** If the woman is unconscious, at that time, she cannot feed her child.

**(Moderator again probed participants to respond, but there were no more responses.)**

1. How long does the mother need to breast feed exclusively and the total duration?

**Participant 1:** Compulsorily for 2 years.

**Participant 2:** Exclusive breastfeeding should be given for 6 months and thereafter, top feeds should be started. The breast milk should be given till the baby is able to feed on its own.

**(Moderator repeated the question; for how long exclusive breast milk should be given then all said in chorus…… six months)**

**Participant 3:** 6 months.

**Participant 4:** Exclusive breastfeeding should be given for 6 months; but many mothers do not feed so long. The breastfeeding can be continued for 3 years.

**Participant 5:** 6 months.

**(Moderator asked again and everybody in chorus told; the exclusive breastfeeding should be given for 6 months)**

**Moderator:** How long the breastfeeding can be continued?

**Participant 2:** Up to 3 years.

**Participant 3:** 2 years.

**Participant:** 2 to 2 and half years, and may be given up to 3 years

**Participant 5:** 2 years.

**Participant 6:** 2 years.

**Participant 7:** 2 years.

**Participant 8:** 2 years.

**Participant 9:** 2 years.

**Participant 10:** 3 years.

**Breastfeeding social norm**

1. Who approves breastfeeding around you?

**Participant 1:** Immediately after delivery, nursing staff will be there with delivered woman. They are the ones who motivate the woman to breastfeed within one hour.

**Participant 2:** After delivery the woman’s mother in law or cousins will be there to help her to breastfeed. During her antenatal period also they approve and motivate her to breastfeed. At the time of delivery doctors and nursing staff will motivate mothers to breastfeed.

**Participant 3:** Pregnant woman’s mother in law, her husband and her mother approve breastfeeding.

**Participant 4:** Her mother and mother in law approves her breastfeeding. Even we (She means Anganwadi workers) should approve and motivate mothers to breastfeed.

**Participant 5:** At the time of delivery nursing staff and doctors tell her to breastfeed. After she goes home her mother, mother in law will tell her about breastfeeding. The most important person to tell her to breastfeed is her mother.

**Participant 6:** The Health worker is the first person to tell her the importance of breastfeeding. Even the family members should support it and approve it.

**Participant 7:** Pregnant woman’s mother is the main person to approve breastfeeding.

**Participant 8:** If pregnant woman’s mother approves then she will listen to her.

**Participant 9:** Her mother is the first person to approve breastfeeding.

**Participant 10:** Woman’s mother.

**Moderator:** “Do you feel husband can approve breastfeeding”?

**All Participants (In chorus): “**No…no….her mother should approve breastfeeding”.

**All supported woman’s mother to approve breastfeeding (All laughed). Only participant 3 agreed that her husband is also an important person to approve breastfeeding but rest all said no.)**

1. Who disapproves breastfeeding around you?

**Participant 1:** Sometimes the breastfeeding mother’ mother or her mother in law disapproves breastfeeding during first few days after birth (She means to say not to feed colostrum).

**Participant 2:** Now a day’s neither we nor their family members disapprove breastfeeding.

**Participant 3:** No one disapproves breastfeeding.

**Participant 4:** No one disapproves breastfeeding.

**(Participant 5, 6 and 7 also had the same opinion)**

**Participant 8:** Now, the days have changed and nobody disapproves breastfeeding. In olden days old ladies, grandmothers used to tell to express the colostrum and throw it away. But now, things have changed; during her pregnancy itself we tell her to breastfeed the colostrum immediately after delivery; and for this family also agrees fully.

1. What do you and people around you think about prelacteal feeds?

**Participant 1:** Honey and sugar water. Initially mother won’t be able to express milk and baby may cry in hunger; so they put two drops of honey and sugar water to baby’s mouth. When they go home, along with breast milk they feed the baby with gutti. (It is a paste made by breast milk with some herbs)

**Moderator:** How many of you agree to this?

**(Participant 2, 3, 5, 7, 9 and 10: Agree for this by nodding.)**

**Participant 2:** If breast milk is not adequate, the doctors prescribe baby with artificial protein milk powder.

**Participant 3:** For the first three days, they don’t feed the baby with breast milk as they believe that the colostrum is not good for the baby because it is yellow in colour. Therefore at that time, they feed the baby with cow milk, honey or sugar water.

**All Participants (In chorus):** Cow milk is given as prelacteal feeds by many people.

**Participant 4:** If woman delivers by caesarean section, she may not be able to breastfeed the baby for one or two days. In that case, they feed the baby with cow’s milk. Some people feed goats milk.

(Participant 8 agrees for this)

**Participant 5:** Honey, sugar water and a mixture of almond, cashew and date are given as prelacteal feeds. If the mother is not having breast milk, people give cow’s milk to the baby.

**Participant 6:** The mothers try to breastfeed their babies. If they do not get breast milk, then they give cow’s milk.

**Participant 8:** People give honey as a prelacteal feed to the baby.

**Participant 9:** I have not seen anyone using pre-lacteal feeds. Presently, they don’t give any pre-lacteal feeds in the hospital or in the community.

**Participant 10:** Sometimes they give some pre-lacteal feeds which we are not aware of.

**Participant 1:** One lady told me that in the community where I work, they rub gold of Mangalasutra (Wedding chain) in water and feed it to the baby as a prelacteal feed. They believe that by doing so the baby will be calm and will become intelligent. I told them not to do so. But they argued that, “we have fed this to all children in our family and for this child also we are giving it”.

**Participant 3:** Some people feed theertha as prelacteal feed. (Water which is used to wash the statue of god, which is believed to be holy by people)

1. What do you and people around you think about colostrum?

**Participant 10:** We tell the people about the importance of colostrum; that it is good for the baby and that it gives immunity power to protect the child from diseases. People think that there is no breast milk for 3 days after delivery; so they do not breastfeed at that time. When we tell them about importance of colostrum they feed it.

**Participant 9:** Mother should feed colostrum to her new born child. People need to start breastfeeding within half an hour after delivery. All breastfeeding mothers in my community feed colostrum to the babies.

(She was very confident in telling this.)

**Participant 8:** Some people tell the mother not to feed colostrum and some people say it should be fed to the baby. People say that “Colostrum should be expressed and thrown away”. People believe that if colostrum is fed to the baby, it may not digest it and may have diarrhoea. When elder people say like this, the mother will be in dilemma to feed the colostrum or not. When we, the health workers say that, the colostrum has immunity power to protect the child from disease, they believe us and feed it.

The people also say that, colostrum is thick milk and the baby may not be able to suck it properly. Sometimes the baby sucks only one breast and leaves the other one. It may cause breast congestion. So they ask the mother to express the thick colostrum and discard it. We should tell them to breastfeed from both sides so that she will not have breast engorgement.

After baby is fed with colostrum, the elders say to mother to express the remaining milk and discard it, otherwise it will cause breast engorgement. People also say not to breast feed when breasts are engorged because it may cause diarrhoea to baby. But we, health care providers tell them to breastfeed the baby even though there is a delay in breastfeeding or if there is breast engorgement.

**Participant 7:** After introduction of ASHA workers and ANMs, there is a lot of improvement in breastfeeding practices.

**Moderator:** What was the situation before they are introduced in health system (ASHA programme was started by government in 2008)?

**Participant 7:** From the time of introduction of ASHA workers, I am seeing the women feeding colostrum to their babies thinking that there is lot of protein in it. Because of the efforts of health care system, there is a lot of improvement in breastfeeding practices. Some people think that, if they feed the baby with colostrum, it may cause jaundice and the baby may have to be kept in baby warmer (He means to say NICU care for neonatal jaundice) and also it may cause dehydration. So they are hesitant to breastfeed the babies with colostrum.

**Participant 6:** Now a day everyone feeds their baby with colostrum. Earlier people used to think that “Colostrum is 9 months old stock milk, so it is better to express it and then start breastfeeding with new, fresh milk.” But presently people are breastfeeding the babies with colostrum.

**Participant 5:** As the participant 6 told, people do have a feeling not to feed the colostrum but to feed the fresh milk which comes after colostrum is expressed and thrown away. Earlier 50% of the people had this opinion, but now, only 10% of people think like that and rest 90% of people breastfeed the baby with colostrum.

**Participant 4:** It is good to breastfeed colostrum as it has immunity power, good for baby’s eyes.

**Participant 1:** If the mother breastfeeds her baby within one hour after delivery, the uterine bleeding will stop. Colostrum has vitamin A and provides immunity to child. If the mother breastfeeds, the enlarged uterus will contract faster.

**Participant 2:** People in the village say that “For the first 3 days after delivery mother won’t get breast milk, so why to breastfeed dry breast without milk?; if the baby sucks that breast it won’t get milk and it may cause pain to the nipples and breasts.”

**Moderator:** How many of you agree for this?

**Note:** Participant 1, 10, 6, 4, 8, 6 agreed that some people do think like that.

**Participant 2:** Now we have removed that misbelief among the people and they breastfeed their babies with colostrum.

1. What do you and people around you think about exclusive breastfeeding?

**AND**

1. What do you and people around you think of gripewater /gutti /any medicines during the first six months?

**Participant 10:** If the baby cries too much, the mother will think that her breast milk is not sufficient for the baby; so they start giving top feeds like cow’s milk before 6 months.

**Participant 1:** Sometimes they give porridge (Rava ganji).

**Participant 9:** For the first six months, they give breast milk only to the baby and nothing else. If the mother takes good food, she will get good breast milk and can breastfeed her baby properly till 6 months. After six months she may give other feeds.

**Participant 3:** Some mothers follow other mothers’ suggestions who say “We have given the milk powder and protein powder to our child and it has grown well”. And without consulting a doctor they start giving milk powder or protein powder formula preparations.

**Participant 8:** We have not seen anyone giving anything to the babies apart from breast milk in the first six months. Very rarely, some people may give cow’s milk.

**Participant 1:** Some people give Lactogen powder (Artificial milk powder).

**Moderator:** Anything else do the people give to their children in the first six months? Gutti?

**All Participants (In chorus):** Yes, they give gutti.

**Participant 10:** They give gutti daily. Even though we tell them not to feed to the baby, they refuse our suggestions by saying “It is good for the baby’s growth and development.”

Gutti is a mixture of almond and dry dates and they give it to the baby daily.

**Participant 9:** In our area people don’t give gutti; but they give gripe water.

**Participant 1, 2, 3, 4, 5, 6, 7 and 10**: Agree with the statement of participant 9 for the usage of gripe water by the community people except participant 8.

**Moderator:** Why they give gripe water?

**Participant 10:** Baby will be calm.

**Participant 9:** Baby will not pass stools repeatedly?

**Participant 7:** It prevents dehydration of the baby.

**Participant 6:** It makes baby sleep well and grow healthy.

**Participant 5:** It makes baby sleep well.

**Participant 4:** Grip water prevents stomach-ache in the babies. If the mothers feel that the baby is crying because of stomach upset, they themselves purchase gripe water from the medical store and feed it to the babies. They give one spoon of it to the baby.

**Participant 3:** They get the gripe water from medical store without doctor’s prescription and give it to babies.

**Participant 2:** No….., all mothers do not give gripe water to their babies on their own; first they consult the doctors. And based on their suggestions only they give gripe water. Sometimes, if the baby is crying or getting irritated too much, the mothers themselves ask the doctor to prescribe gripe water. Gripe water makes the baby calm and the baby sleeps well. Then the mother also becomes relaxed.

**Participant 4:** Earlier, many people used to give gripe water, but now the number of people using it has come down.

**Participants 4, 5, 8 and 9 (In chorus):** In gutti they also add Nutmeg (jajikai).

**Moderator:** Why they add Nutmeg.

**Participant 6:** It makes the baby calm down and sleep well.

**Moderator:** Do people put any medicines to baby in less than 6 months of age?

**Participant 6:** If the baby has fever, loose motion or any other health problem, then only they give medicines; but only after consulting the doctor.

**Participant 7:** Whatever the doctors prescribe, that only they give.

**Participant 8:** For babies, even medical shop people will not give medicine unless it is prescribed by the doctors.

**Participant 9:** If the baby has any problem like fever, then they go to doctor and whatever they prescribe they give.

**Participant 5:** If baby’s body weight is less, then the doctors advise to continue breastfeeding and may prescribe some protein powder.

**Participant 4:** The doctors give bottled medicine with drops. It contains some medicine. If baby is crying too much or restless then only they give it or else no.

**Participant 3:** I haven’t seen any one prescribing any medicine to the baby less than 6 months old.

**Participant 2:** No comment.

**Participant 1:** No comment.

**Participant 10:** If the baby is admitted for any problem, then only the paediatrician will prescribe some medicines if necessary.

**Participant 6:** I have seen paediatrician prescribing calcium, iron and multivitamin medicines to baby for normal growth and development.

**Participant 10:** Even I too agree for this.

**(Participant 6, 5 and 10 agree for additional medicines being prescribed to normal babies whereas Participant 4, 8 and 9 do not agree for this and said “If baby had any problem then only they prescribe otherwise no”. )**

**Participant 7:** “I usually do not prescribe anything to baby for growth and development but I may prescribe multivitamin and calcium to mothers.

1. Why do the mothers think they do not have enough milk?

**Already answered in previous section.**

1. What do you think will help woman to breastfeed longer, do exclusive breastfeeding?

**Participant 10:** By educating the mother she will breastfeed exclusively and longer.

**Participant 9:** We must tell them the importance of breastfeeding. If they breastfeed for longer time, they will not become pregnant early.

**Participant 10:** We must educate and tell them “If you breastfeed, the baby’s growth will be good”. If they do not breastfeed, they may get breast cancer. So we must tell them to breastfeed.

**Participant 8:** If the mother wants her child to grow healthy, she should compulsorily breastfeed.

**Participant 7:** In India all mothers breastfeed their children, it’s in our culture. We need not tell the mother anything extra to breastfeed her child.

**Moderator:** Now we see some people breastfeeding only for two months and then start top feeding. What do you want to say about this?

**Participant 7:** Now a days it has become a fashion in working class women only.

**Moderator:** Is this a fashion among woman who works in the field?

**Participant 7:** No…no….they breastfeed their children.

**Participant 6:** All mothers breastfeed their babies. The mothers know that the breast milk has the power to control diseases in babies. If we tell them, that “Your baby will become wise if you breastfeed” then they definitely breastfeed their babies.

**Participant 5:** In rural area all the mothers breastfeed their children. But it is not so in urban areas because the mothers in urban areas breastfeed less frequently as the women go for work in the morning and come back late in the evening. During that period they leave their children in baby care sitting and it is not possible for them to breastfeed the baby 8 to 10 times as required. If the baby is at home, she can breastfeed only once in the morning and sometimes in the afternoon. It is not possible for them to breastfeed the baby frequently.

**Participant 8:** Now the government has made a rule for working women, to get leave for 6 months so that the mother can take care of child for 6 months and breastfeed exclusively. In private sector also the mothers have the permission for 6 months so that they can take care of their babies.

**Participant 7:** The government rule of 6 month leave to delivered woman is done recently but it was not so before. As our health system is improving in India, such beneficial rules are made.

**Participant 3:** If the mother wants to breastfeed for longer, there should be emotional bonding between the mother and the baby. If the baby is always with mother and if she takes healthy food, she will breastfeed exclusively and for a longer period. The working class mothers can express their milk before going to work and preserve it properly, someone in the family can feed it to the baby. Those women who work in the fields carry their babies along with them to the work area and feed the baby at regular intervals; I have seen mothers taking their babies of less than 3 months.

**Participant 4:** All the mothers breastfeed their babies in my area.

**Moderator probed:** Is there any woman who gives water to her child during summer days? Anyone seen that?

**(Participant 2, 3, 6, 5 and 7 agrees for feeding water before the age of 6 months to baby and the others deny it.)**

1. Anything else you would like to add that you think is important?

**Participant 7:** We must teach all pregnant women about her position while breastfeeding. We must train them how to sit and hold the baby.

**Participant 8:** In villages women wear saree, so it is easy for them to breastfeed the babies even in public places. In urban areas women wear modern dresses which are uncomfortable to breastfeed the baby in public places and it is embarrassing for them to breastfeed the child thinking that someone may see her while breastfeeding. Village women do not care about being seen by others while breastfeeding their babies. As and when the baby cries for breastfeed the mother should be able to breastfeed without any hesitation. While travelling in a bus, the breastfeeding mother should be given a seat. But many mothers are hesitant to breastfeed their child in the public.

**Moderator:** Do you all agree that, if there is no privacy for the women, they may feel hesitant to breastfeed their baby in the public?

**All participants (In chorus):** Yes.

**Moderator:** Some said that women may not breastfeed the baby because of fear of losing their beauty. How many of you agree for this?

**Participant 1, 4, 5 and 6** agree.

**Moderator:** Why do you think so?

**Participant 1:** They do breastfeed, but for a very short period.

**Participant 5:** Women feel that if they breastfeed for longer duration, the breasts become lax; so they do not breastfeed their babies for longer time.

**Participant 4:** They feel that if they breastfeed the breasts become lax, loose the texture which may affect their beauty; so they do not breastfeed for longer time,

**Participant 6:** Yes, there are many such women.

**Participant 8:** If the women do not breastfeed, they will have some problems like engorged and painful breast.

**Participant 6:** If the mother has only female children, they neglect breastfeeding the female child. We need to educate such woman about the complications to her and to her baby if she does not breastfeed; like she may get breast cancer or breast abscess or increase uterine bleeding.

**Participant 5:** We need to conduct mothers meeting regularly during their antenatal period and educate them about the importance of breastfeeding the babies.

**Breastfeeding perceived control**

1. What makes breastfeeding easier?

**Participant 7:** Health education about breastfeeding technique. According to my feeling, female students should be taught about breastfeeding during their High school life. Government had already initiated sex education programme for adolescent girls in schools; likewise breastfeeding should also be taught to them during that period. I feel that it is a better way of education of breastfeeding.

**Participant 5:** In Anganwadi, we conduct mothers meeting for pregnant and delivered women. The delivered women should demonstrate the breastfeeding method to the pregnant women present in the meeting. The primigravidas may not be knowing about how to hold and breastfeed the babies.

**(All Participants strongly agreed for this)**

**Participant 3:** Our primary target to educate about breastfeeding is primigravidas. They are pregnant for the first time and they may not have the knowledge about the position of baby while breastfeeding and how to breastfeed.

Our second target is to educate about breastfeeding are multigravidas (4^th^ or 5^th^ pregnancies). The problem among them is they do not much attention towards breastfeeding because they get tired easily. One more attitude among these groups is, if they did not have any breastfeeding problems in the past postnatal period, even though they had not breastfed properly; then they may not concentrate towards proper breastfeeding in current pregnancy. This problem can be solved by ASHA workers. As per the schedule, the ASHAs have to visit the delivered mothers on 1^st^, 3^rd^, 7^th^ day. The purpose of these visits should be to educate the mother about breastfeeding. ASHA worker should visit the woman wherever she delivers and in her house also. This can bring a change in a bigger way.

**Participant 10:** During ANC check-ups, breast examination should be done and the woman should be told about the correction of retracted nipple if it is found.

**Participant 2:** When we, the ASHA workers visit delivered woman in her house, we need to observe her breastfeeding technique and if she is doing something wrong in the technique of breastfeeding, we need to rectify it as we have been trained in breastfeeding as part of our ASHA basic training.

**Participant 1:** There should be awareness programmes about the importance of breastfeeding through television, radio, street plays and dramas. It will be more effective.

**Participant 8:** I also agree with participant 1. We must educate mothers and mother in laws of pregnant women about breastfeeding and nipple care during ANC period. We may not be able to visit them again and again, so if pregnant woman with relatives like mother and mother in law tells them about it, they won’t feel hesitant and may follow their advice. When the pregnant woman goes to her mother’s place for delivery, the ASHA should educate her about nipple care, breast management like hot water pouring on the breasts to make the breastfeeding easy after delivery. And she may not feel embarrassed if her own mother tells her about these things. If we the ASHAs tell the same things, they may feel shy. It would be more effective, if such things are told by her own mother or relative instead of health care providers.

In mothers meetings we need to call the pregnant women and their relatives like her mother or mother in law. In those meetings, we should explain them about the breastfeeding. If her mother and mother in law is present in the meeting with the pregnant woman, they will follow our instruction seriously. Their mother’s presence creates a lot of impact in accepting and following the breastfeeding education. Sometimes, we may not be present at the time of the delivery; but her mother will be there always and she can help her to initiate breastfeeding properly.

**Participant 7:** Media advertisement through TV and Radio about importance of breastfeeding is very beneficial.

**Participant 3:** The major aspect where we are lacking is lack of involvement of men in breastfeeding practices. We are involving only the pregnant woman, her mother and mother in law in breastfeeding education, but not her husband, father and father in law. We are not involving men in breastfeeding practices. Sometimes, the mother feels scared of breastfeeding her child when she is outside her house because other men will look at her breastfeeding. So we need to educate all the men that breastfeeding is a normal physiological phenomenon. In her home, if her husband himself tells her to breastfeed, it will have a more impact on breastfeeding practices. All the men need to be educated to support breastfeeding.

**Moderator:** How many of you agree for his statement?

**Participant 4:** I agree. The mother may feel shy if any men are there around her while breastfeeding; so all the men need to be told that they are also born to a mother and they were also breastfed. If all the men get involved and were educated about breastfeeding then no woman will feel shy about breastfeeding in public.

**Moderator:** Do all agree for this.

**All Participants (In chorus):** We agree.

**Moderator comment:** So all of you agree for involvement of men in breastfeeding practices. It facilitates breastfeeding practices among woman and she will not feel shy in feeding her child.

1. What makes breastfeeding harder?

**Moderator:** You may have already told that presence of men around, will make breastfeeding harder for woman in public. Similarly is there anything else which makes breastfeeding harder?

**Participant 1:** It would be easy to breastfeed if woman is in traditional dresses like saree but if the woman wears pant and shirt or modern dresses it would be hard to breastfeed.

**Participant 3 and 9:** For working woman it is hard to breastfeed.

**Participant 6:** While the mothers is travelling.

**Participant 10:** If mother is suffering from any disease. If mother herself is not well then she does not feel like feeding the child.

**Participant 3:** Whether the mother health is good or bad, she should breastfeed her child. If she is not feeling well, she may not feel like breastfeeding the child. But she should breastfeed.

1. What does your community think of roles played by maternal depression on breastfeeding?

**Participant 10:** If the woman has female children already and if again she delivers a female baby, then the mother and the family members become depressed.

**Participant 3:** Sometimes in postpartum period, the woman feels that she had done something wrong and become depressed. At that time she neglects breastfeeding.

**Participant 6:** If the woman delivers a male or female baby and if it is not what she was expecting; then also she may undergo depression. This is more common if she delivers female child.

**Participant 5:** If woman is having some personal conflicts with husband and she start feeling disgusting for her life then also she may undergo depression. This will make her to lose interest in breastfeeding.

**Moderator:** How many of you believe that the depression makes woman to lose interest in breastfeeding.

**All Participants (In chorus):** Agree for this.

**Moderator:** Does depression cause reduction in breast milk secretion?

**Participant 3, 8, 9, 1, 6 and 10:** Yes.

**Participant 10:** If woman breastfeeds the child she will have adequate milk production. But if she stops breastfeeding because of depression then her breast milk secretion becomes less.

**Participant 5:** If woman is depressed she may stop eating properly. This causes reduction in breast milk secretion and she breastfeeds less frequently to the child.

**All Participants (In chorus):** Agreed for this.

**Participant 8 and 10:** Delivered women is given bland diet and if she is depressed she doesn’t feel like eating that type of food. This will cause reduction in breast milk.

**All Participants (In chorus):** Agree for this statement except participant 3.

**Participant 3:** If the woman eats properly or not, she will have adequate breast milk secretion.

1. What does your community think of roles played by maternal smoking on breastfeeding?

**Moderator comment:** Except participant 4 and 5 remaining all have seen pregnant or lactating woman chewing tobacco.

**Moderator:** What will be the effect of chewing tobacco on breastfeeding?

**Participant 10:** Nothing will happen on breastfeeding.

**Participant 9:** It causes some discomfort to mother.

**Participant 1:** Baby will lose weight.

**Participant 2:** Those who chew tobacco consume less food. Hence, the baby will also become weak.

**Participant 3:** Baby will lose weight and will hamper baby’s growth and development.

**Participant 5:** Mother may get oral cancer, but nothing will happen to baby.

**Participant 7:** Baby may develop cardiac problem but nothing will happen to breastfeeding.

**Participant 1:** Baby may become mentally retarded. Breast milk secretion becomes less.

1. What does your community think of roles played by maternal drinking on breastfeeding?

**Participant 7 and 1 are the only people who have seen or heard breastfeeding mother consuming alcohol.**

**Participant 1:** I do not know what will be the impact on breastfeeding.

**Participant 7:** Many breastfeeding women in our village drink alcohol. They look very unhygienic. This will cause repeated infection to the mother and the baby.

1. What does your community think of roles played by maternal drug use on breastfeeding?

**No comments from any participants. They have not heard or seen anyone using drugs and its effect on breastfeeding.**

1. What does your community think of roles played by maternal anaemia on breastfeeding?

**Participant 1:** If the woman is anaemic then there will not be any production of breast milk. If at all she has breast milk, it will be very less. The baby will lose weight and get repeated infections.

**Participant 2:** Even though the mother is anaemic there will be adequate breast milk.

**Participant 3:** There will be adequate breast milk; but the mother will become weak and tired. Due to this, the mother may not be able to feed adequately and the baby will be less fed.

**Participant 4:** If the mother is anaemic, it will not have any impact on breastfeeding. But she may feel very weak. But the baby will not get affected.

**Participant 5:** It will not have any impact on breast milk.

1. What does your community think of roles played by maternal overweight or obesity on breastfeeding?

**Participant 1:** If mother is overweight, it causes discomfort for her to breastfed the baby. One of my friend is 120kg, she can’t breastfeed the baby properly and also she has less breast milk.

**Participant 10:** They neither can sit nor sleep properly and feed the baby. They will always be tired.

**Participant 3:** There will not be any problem for breastfeeding in women who are obese. Unlike normal weighing women they may have some difficulty in sitting in proper posture and feeding. But with little support from us it can be overcome. They may not sit for a longer time and breastfeed the baby. If she is comfortable in lying down position and feed, it is okay.

**Participant 7:** The difficulty for such woman is, only having the proper posture and feed the baby.

1. What does your community think of roles played by intimate partner violence on breastfeeding?

**Participant 5:** Yes, it will have impact on breastfeeding. IPV may cause depression among woman and negligence towards baby. The woman may become frustrated of life. She will neglect the child care and also the breastfeeding.

**Participant 10:** Normally woman have to breastfeed at least 8 to 10 times and if there is IPV she may feed the baby less frequently.

**Participant 1:** If there is IPV, woman will be always thinking about it and neglect breastfeeding the child. There will be less production of breast milk if she keeps on thinking the same. If she doesn’t worry much about the violence, then there will be a good production of milk and she can breastfeed the child adequately.

**Participant 3:** IPV will not have any impact on breastfeeding.

**Participant 2:** They will not take adequate care of the child and there will be less production of breast milk.

**Participant 7:** IPV will not have any effect on breastfeeding.

**Participant 6:** Personally the woman may suffer from emotional pain and she may neglect her child; but there will not be any effect on her breast milk production and breastfeeding.

**Participant 8:** Women will be depressed and they may not feed the child. There will not be any decrease in breast milk production.

**Participant 9:** The woman will forget about such IPV and breastfeeds the child normally.

**B. Acceptability of breastfeeding peer counselors (10 minutes)**

1. Where do you get information about breast feeding?

**Participant 8:** From Anganwadi centre.

**Participant 1:** We conduct mothers meetings, village Health and Nutrition day programmes; in those meetings, we tell the pregnant woman and delivered women about the importance of the breastfeeding.

**Participant 3:** Her mother is a first source of information about breastfeeding.

**Participant 4:** Her relatives like mother in law, mother and sister will give information to woman about breastfeeding. The woman gets information about breastfeeding from home first, then from other health care providers like ASHA, ANM and Anganwadi workers.

**Participant 7:** From her partner too (Husband he means).

**Participant 4:** From neighbours those who have delivered or breastfed the child.

**Participant 5:** Health care providers are the first people to share information to the woman about breastfeeding practices. Even though we share first-hand information, the most effective will be, when they receive breastfeeding information from her family members.

**Participant 4:** I too agree for this.

**Participant 10:** The woman may have information about breastfeeding well in advance, but at the time of delivery we (Staff nurse) motivate and help her in breastfeeding. They may not be knowing anything unless until we motivate them to initiate breastfeeding.

**Participant 9:** From her mother.

**Participant 8:** Form mothers meeting and from her mother.

1. If you have any breast feeding problem whom do you approach?

**Participant 10:** For first 48 hours after delivery, the woman will be with us (She is a staff nurse) in the hospital and if they have any problem in breastfeeding they will consult us.

**Participant 9:** They consult family members. If they cannot solve it then the mother will consult doctors. If the mother has fever and breast engorgement they will consult doctors.

**Participant 8:** If woman has breast engorgement (called as mole todaku in Kannada language) they tell their mother, that they have pain in the breast. Afterwards they will consult doctors.

**Participant 7:** The home remedy is commonly done for any problem associated with breastfeeding by pouring hot water on breast, breast massage and expressing breast milk when they are engorged. If treatment in home by family members doesn’t solve the problem, then they will go to doctors.

**Moderator:** Do all agree for this?

**Except participant 3 all the other participants agree that mother will consult family members first, then they go to the health care providers.**

**Participant 3:** If breastfeeding mother has any problem she may consult doctors or family members or ASHA workers, ANM or any other health care providers, whom she trusts more and believes that her breastfeeding problem may be solved by consulting with them. Some mothers may directly call the doctors or ANM or ASHAs on phone and tell their problems.

Mother will tell her problems to those who shows more concern and care towards her and help her in solving the problem.

**Moderator:** Do they share their breastfeeding problems with their husband?

**Participant 4, 10, 1, 8 and 6:** Agree for this.

**Moderator:** Do they share their breastfeeding problem first with her mother.

**All Participants (In chorus):** Agree.

1. Are you aware of any programmes by the government to promote breast feeding practices in the country?

**Participant 1:** Breastfeeding week.

**Participant 3:** Infant and young child feeding programme (IYCF)

**Participant 4:** Breastfeeding week.

1. Is there any support from the women from the village/community?

**Participant 4:** Yes, there is a support from the community about breastfeeding.

**Participant 8:** People motivate her to breastfeed if baby starts crying.

**Participant 1:** People provide privacy to breastfeeding mother which help them to feed the baby comfortably.

**Participant 4:** People provide all kinds of support to pregnant woman.

**Participant 5:** If breastfeeding mother goes to any place, community members provide privacy to mother, so that she can breastfeed the baby easily.

**Moderator:** Is there any support from the community or villagers if women has any breastfeeding problems?

**Participant 1:** Villagers help woman if she has any breastfeeding problems. They tell to and even sometimes do breast massage, pouring hot water on breast when woman has breast engorgement.

**Participant 6:** People who have experience in dealing with breastfeeding problems give suggestions to breastfeeding mother to overcome it. There are many misbeliefs in the village like waving lock chain in front of chest (In kannada it is called chilaka ilisodu) Which will help woman to get relieved from breast pain. The villagers do these things which may support the woman emotionally.

**Participant 1:** Waving measuring cup. (In Kannada it is called as **“**Sher alagadisodu”)

**Participant 9:** Casting spell or witch treatment. (In Kannada it is called as **“**Mantra hakodu”)

**Participant 3:** If mother has any problem in breastfeeding community people arrange transport for her for referral. If they know any good doctor who can treat such problems, they will recommend her to them.

**Participant 8:** Villagers and community people prepare energy rich food like edible gum laddu (Antina undi), coconut sweets (Khobbari Khara) to breastfeeding mother which help her in production of good amount of milk. This is a common practice in our culture.

**[Moderator comment:** All participant agrees for giving energy rich food to breastfeeding mothers which facilitates for good breast milk production]

**Participant 2:** Villagers/ community people give Semolina (Rava), coconut, sugar, ghee to breastfeeding mother so that these calorie rich food help mother in production of good quality breast milk.

**Participant 4:** Yes, community people give ghee to the mother.

**Participant 5:** They also give garden cress seeds (Alavi)

**Moderator:** Apart from giving food supplement to breastfeeding mother, is there any other support villagers or community people provide?

**Participant 2:** Villagers or community may give cloths to new born and the mother, like infant cap, infant bed sheets ect. (kunchige, dubati)

**Participant 4:** If the mother does not have adequate breast milk and if there is a neighbour is also breastfeeding, then she can feed both the children.

1. Is she trained in giving breast feeding support and help?

**Participant 3:** If she is a mother and has breastfed 2 or 3 children, she will be having the experience of breastfeeding.

**[Participant 10, 1, 6, 4 and 9 agree for this statement]**

**Moderator:** Does any health care providers are trained especially in breastfeeding?

**Participant 3:** Yes, I am trained in breastfeeding. (He is a doctor)

**Participant 7:** I am not trained in breastfeeding but have read about it in the books. (He is a private practitioner doctor)

**Participant 1 and 6:** We are trained.

**Participant 4:** We are not trained but experienced in it by repeated hearing from other health care providers.

**Breastfeeding peer attitude**

1. Do you think that a person (Peer Counsellor) from your community/village if trained will be helpful to solve the breast feeding problems?

**All Participants (In chorus):** Agree that such peer counsellor can solve the breastfeeding problems of the community if they are well trained.

**Participant 3:** It is better if such peer counsellor trains someone in the home of breastfeeding mother. If the couples also (means husband and wife) are trained then it would be still better.

**Participant 7:** Breastfeeding is nature’s gift; so if the breastfeeding mother is trained in home, then it is good.

**Participant 3:** Breastfeeding woman may not share all the problems with peer counsellor, because she is scared of breach of confidentiality. It is better if both husband and breastfeeding mother are trained by peer counsellor, so she can express her problem freely.

**Participant 5:** Peer counsellors alone may not be able to train and monitor regularly about the breastfeeding, breast hygiene and posture of breastfeeding. So I feel it is better to train one family member about breastfeeding by peer counsellor.

1. Do you accept receiving Breast feeding counselling and advice from such a person?

**All Participants (In chorus):** Agree for breastfeeding counselling and advice from peer counsellor.

**Participant 3:** I too agree but peer counsellor should be from the same village.

1. What should be the qualifications of Peer Counsellor?

**Participant 3:** She should be from the same village. She need not have any educational qualification. She should be a mother and must have breastfed. If we involve outsider as a peer counsellor then the impact would be very less.

**Participant 8:** She should be from same village and the community. There is no need of any school education.

**Participant 10:** It would be better if she has some schooling. If she is educated well, the breastfeeding mother will have more trust for whatever training or counselling the peer counsellor does.

**Participant 5:** She should be influential and community people should agree for whatever peer counsellor tell or train.

**Participant 1:** She should be trustworthy in the community. She should be well educated and should be able to tell and solve the problems efficiently if breastfeeding woman has any problem. If she knows well about the breastfeeding she can effectively train the mother about breastfeeding; otherwise it will be difficult.

**Participant 2:** She should talk sweetly and politely, then only villagers will accept her. If she is short temper people will not accept her.

**Participant 1:** She herself should have breastfed exclusively her baby for 6 months and should have done continued breastfeeding for 2 years along with weaning food.

**Breastfeeding peer perceived control**

1. What would be the benefit to have Peer Counsellor?

**Participant 3:** The breastfeeding mother will have hands on training about breastfeeding.

**Participant 1:** The breastfeeding women in the community will have extra benefit of peer counsellor in relation to solving the problems of breastfeeding. Breastfeeding mother will have more training about breastfeeding the child.

**Participant 10:** Training the mother in breastfeeding itself is not enough and it is necessary to give demonstration about breastfeeding technique. Training orally is less effect than practical demonstration. Peer counsellor should tell about breastfeeding technique and self-examination of breast for retracted nipple. I have seen many women with retracted nipple. All health care providers tell orally. But these retracted nipple problems can be solved by proper training of the woman and her family members through demonstration by the peer counsellor.

**All participants (In chorus):** Agree that there is a benefit for having peer counsellor.

1. What would be the barriers/problems to have Peer Counsellor?

**Participant 3:** The caste and the religion of the peer counsellor would be a barrier. If the peer counsellor is of the same caste and religion as of breastfeeding mother then they will accept her happily if otherwise people may oppose her.

**Breastfeeding peer social norm**

1. Who approves the presence of Peer Counsellor?

**Participant 10:** Community people or villagers.

**Participant 1:** Villagers; local government representative like elected panchayat members.

**Participant 6:** Community people should accept peer counsellor. Peer counsellor should have harmonious relation with all the people in the community.

1. Who disapproves the presence of Peer Counsellor?

**Moderator:** As you have already told, on the basis of caste and religion she can be disapproved. Is there anything else on which she can be disapproved?

**Participant 7:** If she is ugly looking then also she can be disapproved by the community.

**Participant 3:** Her pervious history of whether she is mentally sound or not, will also decide to approve or disapprove her as peer counsellor.

**C. Program to train breastfeeding peer counselors (10 minutes)**

1. Tell us about your practice on breastfeeding with women before they deliver.

And

1. What do you talk about breastfeeding antenatally?

And

1. What happens at birth?

**Participant 1:** We meet pregnant woman during antenatal period and before she goes to mothers place for delivery, we

- Look for whether there is quickening felt by mother?
- Educate about personal hygiene.
- Tell her to avoid wearing tight bra.
- Educate her about exclusive breastfeeding for 6 months.
- At delivery we promote her to start breastfeeding immediately.

**Participant 6:** We educate her about exclusive breastfeeding, to decide the place for delivery, about the nutrition to prevent malnutrition and anaemia during pregnancy.

**Participant 2:** We educate her about regular ANC check-up and consuming nutritional food.

**Participant 7:** We give moral support and encourage her saying that “Nothing adverse will happen during delivery”.

**Participant 1:** We educate them antenatal about arranging cloths for new born baby.

**Participant 5:** We give health education about various topics like USG scanning, expected date of delivery and to get admitted in case of postdatism?

**Participant 4:** For primigravidas, we take more care as they will not be knowing much about breastfeeding. We tell them not to wear tight bra, self-examine breast and massage breast with oil while taking bath and care of nipple to avoid retraction. There was one primigravida mother in my area and I had told her the same advice; but she did not listen to it, finally she had breast engorgement due to retracted nipple after delivery, which caused problem to her and the baby. So I feel primi mothers should be educated more about breastfeeding.

**Participant 1:** We demonstrate the position of breastfeeding to delivered woman, that is how to hold the baby. About the position of the baby during breastfeeding.

**Moderator:** How is the breastfeeding practice soon after delivery?

**Participant 1:** Then woman will start breastfeeding 10 to 15 minutes after the delivery.

**Participant 2:** There may be a delay of more than 30 minutes after delivery to initiate breastfeeding.

**Participant 3:** They breastfeed immediately.

**Participant 4:** They breastfeed immediately.

**Participant 5, 6, 7, 8, 9 and 10:** All agree that mother will start breastfeeding soon after delivery.

1. How frequently do you visit the mother after birth?

And

1. What is the purpose of visit?

**Participant 4:** We visit once to her house as soon she comes from the hospital after the delivery. We enquire whether woman has started breastfeeding the baby and even we observe her breastfeeding. We tell mother that baby will urinate more if she breastfeeds more frequently, and that is good sign of healthy baby.

**Participant 8:** We also ask her about bleeding per vagina. We ask her about breastfeeding and tell that vaginal bleeding will decrease if she breastfeeds the baby. We enquire about when she started breastfeeding. Educate her about personal hygiene. We visit woman’s house once a week after her delivery. We visit woman’s house as soon as she comes from hospital after delivery, next we visit her after 5 days. Thereafter, we visit her house on weekly basis. We go to her house at 1 and half months postpartum to call her for baby’s immunization, and there after regularly as per schedule of baby’s immunization.

**Participant 4:** Immediately after delivery we visit woman’s house on weekly basis. After 1 month we go to call her for immunization of the baby. Thereafter, at 1 and half, 2 and half and 3 and half months postpartum period.

**Participant 1:** We visit 7 times after woman delivers that is on first day and then as per scheduled visit. It is not possible for us to visit 7 times in 42 days but we do visit 2 to 3 times during that period.

**Participant 6:** We visit once in a month, at the most we can visit 2 to 3 times during one month postpartum period.

**Participant 2:** We visit 4 to 5 times in the 1^st^ month of postpartum period. We visit on the 1^st^ day postpartum in the hospital; then on 3^rd^ day, 5^th^ day, 7^th^ day, 14^th^ day, 21^st^ day and at 42^nd^ day.

**Participant 9:** I too visit in the same way.

**Participant 5:** We also visit woman in postnatal period once in fortnight. There is no fixed schedule for us to visit post-partum women’s house.

**Participant 3 and 7:** We are doctors and we do not visit mother’s house.

1. How frequently you assess breast feeding practices of the mother?

**Participant 1, 2, 4, 6, 8 and 9:** Agree for whenever we visit the delivered woman’s house, we assess breastfeeding practice of the mother.

**Participant 2:** We make woman to breastfeed the baby in front of us and if she has any problem we help to solve it.

**Participant 1:** As soon as we visit woman house, we enquire whether the baby is breastfeeding or not. I ask her to breastfeed the baby in front of me. If there is any problem or fault in breastfeeding technique, I advise her accordingly.

**Participant 7:** We advise her to breastfeed the baby properly. I tell her that baby’s mouth should be covering the areola completely while breastfeeding. I ask her to breastfeed the baby in front of me and advise her if there is any problem of breastfeeding.

**Participant 2 and 9:** We also ask mother to breastfeed the baby in front of us and assess her breastfeeding technique. If she does any mistake in breastfeeding we rectify it.

**Participant 8:** I assess breastfeeding process of the mother. I advise her to breastfeed the baby regularly and after feeding take the baby on the shoulder and rub the back till the baby gets burp. I also advise them not to breastfeed the baby in lying down position. Even though it is night hours when she is sleeping, I instruct them strictly to sit-up and breastfeed.

**Participant 4:** I observe the woman while breastfeeding the baby. We check the weight of baby monthly and if the weight is increasing, then we consider it as the baby is breastfeeding adequately.

**Moderator:** When you visit breastfeeding woman’s house [to **Participant 1, 2, 4, 6, 8, 9]** you advises them about breastfeeding and correct them if they are breastfeeding improperly. Do you follow them up to see whether they are following your advice and assess that they are breastfeeding the baby in proper posture and technique?

**Participant 1, 2, 4, 6, 8 and 9:** Yes, we do follow them up.

1. How many mothers you have to visit per day/per month?

**Participant 1:** I do 5 visits per day and meet 10 to 12 women per month.

**Participant 2:** I visit 4 to 5 women per month.

**Participant 4:** I visit 10 mothers per month. Under the governments nutritional programme called Matrutva programme (mid-day meal to delivered women) we call them daily to anganwadi to have lunch, at that time we observe and enquire about the breastfeeding also.

**Participant 7:** During my medical practice I see at least one breastfeeding mother daily in my clinic.

**Participant 8:** Daily 3 to 4 breastfeeding mothers come to my anganwadi and I observe their breastfeeding method.

**Participant 3, 5, 7 and 10:** We do not visit breastfeeding mothers in their house, but we see them in the hospital.

1. What are the common breast feeding problems you observe in your community?

**It is already answered in earlier questions.**

1. Do you think that peer counsellor who is from the same community would help and support mothers?

**All Participants (In chorus):** Yes, the peer counsellor who is from the same community would help and support the mothers in breastfeeding.

1. What do you think would be the barriers for Peer Counsellors in the community?

**Moderator:** As you have already said that there would be barriers like caste and religion of the peer counsellor, would there be any other barrier for the peer counsellor to work in the community?

**Participant 1:** There would be politics arising in her work.

**Participant 8:** I too agree for this. If you select an individual as peer counsellor, the other eligible candidates who is not selected may feel jealous about her and may create some problems in her work.

**Participant 7 & 5:** We also agree with the opinion of participant 8.

**Participant 4:** People feel that "already so many health care providers like ASHA, ANM and Anganwadi workers are advising the mothers about breastfeeding; and what extra things these peer counsellors are going to tell about breastfeeding?" the mothers may become reluctant to accept the advice by the peer counsellor. People may get annoyed by having the same advice about breastfeeding by another person like peer counsellor.

**Participant 3:** There is a possibility of eversion from the community towards breastfeeding knowledge given by peer counsellor. People may start doubting "Why they are telling and training repeatedly?".

**Participant 7:** I also agree with what participant 3 has said. I feel breastfeeding education should be given in schools.

**Participant 1:** Publicity through various media involving peer counsellors would be good.

**Participant 4:** Teenagers like students 10^th^, 11^th^ and 12^th^ classes in the school should be educated about breastfeeding rather than involving peer counsellor.

**Participant 8:** Mothers meeting conducted by us is a better place to educate about breastfeeding than by peer counsellor. (She is a Anganwadi worker)

1. Will there be future opportunities for these Peer Counsellors to continue in the community?

**Participant 8 and 4:** Yes, there will be opportunities for them in future.

**Participant 3:** No sir, they will not have any future opportunity. To do this work, ASHA workers are already there in the community. So one more cadre of peer counsellor only for breastfeeding will not have any future perspective.

**Participant 4:** We anganwadi workers, ASHAs, ANMs and even doctors tell pregnant women about breastfeeding; people get irritated with the new type of worker as peer counsellor telling the same thing about breastfeeding.

**Participant 1:** Nothing like that. When, already ANMs, AWW, and ASHA workers are telling about breastfeeding and if the peer counsellors also tell about breastfeeding the community people will feel the importance of breastfeeding. When peer counsellors are specially appointed for the specific task of breastfeeding counselling and training, they take breastfeeding more seriously and try to concentrate and accept the education and training given by them.

**Participant 7:** When other health care providers are telling about breastfeeding, the community people may question the ability of peer counsellor in advising anything new concept of breastfeeding. They may question "Is she telling something which we already don’t know?".

**Participant 6:** I am an ANM, we have to serve a large population and it is already a burden for us. So we may not be much effective in breastfeeding counselling and training. We have got so much of administrative and reporting work to do. Therefore, we concentrate less about breastfeeding programme. The same thing happens with other health workers also, when they have got more writing and reporting work. It is my worry that whether the peer counsellor also work in the same way or concentrate more on practical issues of breastfeeding.

**Moderator:** Suppose if we ensure to train the peer counsellor properly and if she concentrates more practically in counselling, training and solving breastfeeding problems, do you think they will have a better future?

**All Participants (In chorus):** Yes, they will have future.

**Participant 7:** The peer counsellor should be more practical in her activity than being theoretical. If she works as we do then it is of no use, they should be more practical.

**Participant 8:** The peer counsellor should conduct mothers meeting of all pregnant and delivered women; she should tell them what she will be going to do. If those women understand and accept her activity, then she will have a future.

**Participant 2 & 9:** Yes, they will have future.

1. What should be criteria for acceptance of such counsellors by you and the community?

**It is already discussed in question 8 of section B.**

1. How do you identify and recruit such counsellors?

**It is already answered in previous question.**

1. Do you think training of peer counsellors is necessary?

**All Participants (In chorus):** Yes, it is necessary.

1. What should be the content of the training?

AND

1. What knowledge and skills should be taught to them?

**Participant 1:** There should be a practical demonstration of breastfeeding technique during training.

**Participant 7:** The peer counsellor should not depend on payment. She should work as if it is a social service. She should feel proud that she has been selected for a noble cause of teaching the mothers about how to breastfeed.

**Participant 9:** She should be trained about how to breastfeed, about the problems of breastfeeding and how to solve them.

**Participant 2:** The peer counsellor should be trained in breastfeeding by practical demonstration.

**Participant 1:** The training should include real life breastfeeding mother and baby. The training should be by practical demonstration about the proper technique in breastfeeding.

**Participant 8:** There is no point in training the peer counsellor theoretically. She should be trained by practical demonstration, case based problem solving, role playing of problems and solutions. By this way, she will be well trained and will manage the problems of breastfeeding easily.

**Participant 4:** Peer counsellor should be trained in detail about breastfeeding techniques, problems of breastfeeding, advantage of breastfeeding, problems encountered by mother and how to solve them. If she has through knowledge of breastfeeding, then only she can train or counsel others.

**Participant 1:** She should be trained about advantages of breastfeeding and disadvantages if baby is not breastfed.

**Participant 7:** Training should include breast exercise, posture, position of breastfeeding and nipple care. She should be trained in all the aspects of breastfeeding.

**Participant 3:** Peer counsellor is not required. The existing health care providers like ASHA, AWW are receiving breastfeeding training or refresher training once in 6 months. During breastfeeding week celebration also the medical officer trains them about breastfeeding. Everyone is trained only theoretically. That training includes mouth of the baby should be covering complete areola, proper latching should be there and so on. These trainings are done orally. If at all they need improvement, the audio visual aids should be used. Practical demonstration of breastfeeding should be done through audio visual aids on the screen or virtually. There should be hands on training.

**Participant 1:** There should be separate baby breastfeeding room where peer counsellor should be trained in presence of breastfeeding mothers.

1. Can we use mobile device to help Peer Counsellor counsel the mothers regarding optimal Breast feeding practice?

**All Participants except Participant 6 (In chorus)**: Yes, mobile device can be used by peer counsellor to counsel the mothers regarding optimal breastfeeding practice.

**Participant 6:** I don’t think mobile application would be more helpful than telling them personally. Already there are many pictures and videos and I don’t think mobile application can additionally improve the breastfeeding technique.

1. How do you think using Mobile app would help Peer Counsellor?

**Participant 1:** Training would be more effective for breastfeeding mother by using mobile health app than simply telling. She will learn more quickly by watching than listening. There should be the detailed technique of breastfeeding. The method of breastfeeding the baby, the posture, the technique, whether the baby taking the feed properly or not etc.

**Participant 2:** I too agree with participant 1. Watching the video is more effective than listening to what we tell.

**Participant 10:** There should be information about personal hygiene, hand washing technique, the method of holding the baby, position of the baby while breastfeeding and burping technique.

**Participant 9:** There should be pictures along with sub titles. There should be a video of breastfeeding technique, the problems encountered while breastfeeding and how to solve them.

**Participant 8:** I feel that the problems of breastfeeding should not be shared with mother. Only positive aspects of breastfeeding should be shown to mothers through mobile app.

**Participant 5:** Mobile should include positive as well as negative aspects of breastfeeding. There should be video to show these.

**Participant 4:** The mobile app must include everything the pregnant woman is supposed to do from the time of her antenatal period till 6 months postpartum about breastfeeding.

1. What will be the barriers using m-Health by the Peer Counsellor?

**Participant 10:** The m-Health app should be used to train only the breastfeeding mother and her family members but not to others. If she shares the video with other people it may itself cause a barrier.

**Participant 9:** There are no barriers for using m-Health app by peer counsellors.

**Participant 8:** The peer counsellor should ensure the proper circumstance to train the mothers with m-Health app. If the peer counsellor insists the mother to watch the video when the woman is not in a good situation to watch the video, it will be a barrier.

**Participant 5:** When the woman is busy in household work it would be a barrier to watch the video. The peer counsellor should visit the mother’s house when she is free to spend time for training with m-Health app.

**Participant 3 and 7:** There are no barriers.

**Participant 2:** All women in the house should be trained simultaneously along with the breastfeeding mother. Otherwise they may make some comments which may cause barriers for the peer counsellor in future to train and counsel the mother in breastfeeding.

**Participant 4, 5, 7, 3 &1:** There are no barriers for using m-Health app in training about breastfeeding.

**F. Wrap up**

1. *Ask participants*

**“How did you feel about participating in this session? What was easy? What was hard?”**

1. *Summarize and thank participants by saying:*

**“We appreciate all of your great ideas. You have been a big help, and we want to thank you very much for all the information you have shared with us today. We know that your ideas will help to make this a successful program and technology. Thank you once again for your participation – we really appreciate you!”**

**Thank you.**

**Focus group interview guide**

**Materials Needed:**

Food and Beverages

Newsprint & Markers

Masking tape

Nametags

Audiotape recorders and tape

Participant incentives

Questionnaires

Pencils

Attendance sheet

**Introductions and Focus Group Process (10 minutes)**

1. *Team members will have nametags on prior to participants entering the focus group venue*
2. *Give participants nametags as they arrive.*
3. *Instruct participants to select celebrity name*
4. *Have participants sign-in on attendance sheet.*
5. *Explain the purpose of the focus group session by saying:*

**“Welcome to today’s focus group. We are planning to develop mobile technology to support breastfeeding peer counselors and breastfeeding mothers. We asked you to come to today’s session because we would like to hear from you about your opinions and ideas on the technology content. You are the experts, and we can learn from you. We need your honest opinion – good and bad – about what we are trying to do. We would like you to share what you think – and what you think other members in your community might think about our project. Everything you are thinking is important to us. There are no right or wrong answers. We value your opinion. We would be very happy if you would help us to make the best technology possible.**

**Please remember to use your celebrity name and refer to others with their celebrity names. Also, try not to use any friends’ names or specific locations. But if you do, we will delete them from the audio recording.**

*As a reminder we are going to turn on the tape recorder now.*

*Is everyone ok with that? TURN TAPE ON: and announce*

“This is focus group # 3 on 9^th^ March 2018 at PHC Belawadi the “BEST4Baby”

*1. Ask the participants to introduce themselves by saying their celebrity name and what is your favorite color.*

*2. All members of investigative team introduce themselves.*

**“Now, we would like to develop some group rules so we can learn as much as possible from each other.”**

**Group Rules (10 minutes)**

1. *Develop group rules to protect participants’ confidentiality. Offer examples of group rules if participants are stuck:*

Be respectful

Be honest

Maintain confidentiality

One person speaks at a time

Listen to others

No put downs or insults

Turn off all cell phones

**“Even though we will be talking about breastfeeding and technology development today, we do not require you to talk about your personal experiences with breastfeeding if you do not feel comfortable. You can instead share what you think or others’ experiences. However, please do not use any specific names or identifiable information of others for protecting their privacy. Is that clear to everyone?”**

**BREAST FEEDING EDUCATION SUPPORT TOOL for BABIES**

**Focus Group Discussion guide for Mothers who**

**Have successfully breastfed at least one child in the past three years**

**Discussion (40 Minutes)**

**“We are working to develop a mobile technology to support breastfeeding peer counselors and breastfeeding mothers in India. We want to know what you think and what you know about breastfeeding practices. Your inputs will certainly help develop feasible and effective technology to support breastfeeding. We have questions we prepared but we will let our group guide our own discussion. Let’s start with some initial questions about breastfeeding experiences.”**

1. ***Breastfeeding practice and support mechanism (20 minutes)***
2. Tell us about your breast feeding experience

**Participant 1:** I had difficulty in breastfeeding during the first two months. I had cracked nipples and it was painful. I squeezed the milk from my breast and fed the milk to the baby with spoon during that time. I took injection for that and after three months, I started breastfeeding the baby.

**Participant 2:** After my first delivery, I was told to massage the nipples while taking bath. I had some problem with my nipple and due to that, I had difficulty in breastfeeding initially; but there after I had no difficulty.

**Moderator:** For how many days you breastfed your baby?

**Participant 2:** For the first child I breast fed for two years and for the second child one and half years.

**Participant 3:** My babies were low birth weight. They were kept in NICU for 15 days at government district hospital. During that time, I used to express milk from my breast by pressing breast and used to feed both the babies with spoon.

**Moderator:** That was when the babies were inside. What about when they were discharged from NICU?

**Participant 3:** When they were discharged from NICU the babies started breastfeeding themselves.

**Moderator:** After how many days they were discharged from NICU?

**Participant 3:** The babies were discharged after 15 days.

**Moderator:** Then you started breastfeeding both the babies?

**Participant 3:** Yes, for both babies (Twin babies).

**Participant 4:** My first delivery occurred in Hubli Medical College Hospital. That baby’s weight was less. It was one kg and five grams. Doctors told me that the baby had difficulty in breathing and told that the baby has to be kept in NICU for two days. I had retracted nipple. I was told to express the breast milk with the help of a syringe (she meant vacuum pump) and feed the baby. I took out the milk and fed it to the baby with the help of spoon. For the second child also I did the same thing.

**Moderator:** For how long you fed the baby?

**Participant 4:** For the first child I breast-fed for 2 years. And for the second child I breast fed it for six months. Now the baby is one year and two months old and now it is not breastfeeding.

**Moderator:** How old is your second child?

**Participant 4:** Now it is one year and two months old.

**Moderator:** How long you breastfed your second child?

**Participant 4:** Two years.

**Moderator:** That was for the first child. What about the second child?

**Participant 4:** I have breastfed my second child for six months. Now also it is breastfeeding.

**Moderator:** But you told six months.

**Participant 4:** Top milk.

**Moderator:** You started giving top milk along with breast milk.

**Participant 4:** Yes.

**Participant 5:** Immediately after birth, breast milk is very important for the babies.

**Moderator:** Tell me about your experience.

**Participant 5:** We need to breastfeed the baby. My first delivery was caesarean and second delivery was also caesarean. I have two children and I have breast fed both of them.

**Moderator:** For how long you have breast fed them.

**Participant 5:** I have breast fed my first child for two years. The second baby was not well; that’s why I am still breastfeeding her.

**Moderator:** How old is she now?

**Participant 5:** She is two and half years old.

**Participant 6:** Mine was a normal delivery in government hospital. There was no problem for me.

**Moderator:** How long did you breastfeed?

**Participant 6:** I have only one girl child. She is two and half years old and still I am breastfeeding her.

**Participant 7:** My delivery was a caesarean in government hospital. I got my breast milk secretion after three days. I got watery milk and that was also insufficient. My child is eleven months old now.

**Moderator:** Is this the only child for you?

**Participant 7:** Yes.

**Moderator:** And are you breastfeeding the baby?

**Participant:** Yes, I am breastfeeding her. But the breast milk is insufficient for the child.

**Participant 8:** Mine was a normal delivery in tertiary care private Hospital. To breastfeed the child, I had no nipple. My breasts were full of milk, but for one month, the baby could not breastfeed. Now, from the second month the baby is breastfeeding.

**Moderator:** How old is your baby?

**Participant 8:** It is two months completed and is in third month.

**Breastfeeding attitude**

1. Do you think breastfeeding is important for the baby?

**Moderator:** According to your opinion breastfeeding is good or not?

**All Participants:** It is good to breastfeed.

**Participant 1:** By breastfeeding the babies will improve; the baby’s weight also increases. By feeding the top milk, the baby will have sputum. Due to sputum, the baby will have problem. Therefore, the babies have to be breastfed.

**Moderator:** What do you say?

**Participant 8:** I did not have nipple.

**Moderator:** Do you agree that breastfeeding should be done or not?

**Participant 8:** Yes, I agree for breastfeeding. I tried very much to breastfeed; I was given a syringe to takeout the milk and feed to the baby. (She meant breast pump)

**Moderator:** You, number 7. Do you agree for breastfeeding or not?

**Participant 7:** Yes, we should breastfeed.

**Moderator:** You. (To number 8)

**Participant 8:** Yes, I too agree to breastfeeding.

**Moderator:** Not only you, but other mothers also should breastfeed or not? Do you all agree?

**All Participants:** Yes.

**Participant 2:** Initially I was not ready to breastfeed; but the doctors advised me to breastfeed the first milk to baby even though it is thick and difficult to feed.

**Moderator:** Do you agree for breastfeeding?

**Participant 2:** Yes.

1. Why? What are the benefits?

**Moderator:** What are the benefits of breastfeeding? You tell number 3.

**Participant 1:** My baby fed milk, which was like blood and pus. There was no breast milk at all. We tried to give cow milk; the baby did not take it.

**Moderator:** Yes, you have tried to breastfeed. But what are the benefits of breastfeeding?

**Participant 1:** We breastfed our baby thinking that it will be fine.

**Moderator:** What will be fine?

**Participant 1:** The baby grows well; weight of baby will increase.

**Moderator:** What do you say? (To number 2)

**Participant 2:** There is protein in the breast milk. Therefore, it should be fed. The mothers are having iron in their body and it is good for the baby.

**Moderator:** What do you say number 3? Why the baby should be breast fed?

**Participant 3:** For the first 6 months after delivery, the baby should be breastfed. Thereafter, it is okay if we feed anything else.

**Moderator:** Why? Why do you give importance to it?

**Participant 3:** We should breastfeed the baby so that it will remain healthy.

**Moderator:** Anything else you want to say.

**Participant 4:** Apart from our baby, we can breastfeed other babies also whose mother don’t have breast milk. My own experience is that; my elder sister had delivered three babies at a time (Triplets). One of those babies died due to breathing difficulty. For remaining two babies she did not have breast milk. Doctor advised me to feed those babies because she did not have breast milk.

**Moderator:** Do you mean to say that, you breastfed your own baby and those two babies as well.

**Participant 4:** Yes. I fed my baby and my sister’s babies also. If we eat vegetables, we will have more milk. Earlier also, I used to have more breast milk. Even when I was pregnant, I had more breast milk. I used to keep handkerchief for both of my breasts and I had told this to the doctor. The doctor said it would be all right.

**Participant 5:** Breast milk is very important and necessary for the child to grow and become healthy.

**Moderator:** Is it? Are there any other benefits? What do you say number 6?

**Participant 6:** I am also of the opinion that the breastfeeding is good for the baby.

**Participant 7:** I am also of the same opinion.

**Participant 8:** The baby will not get any disease.

**Participant 2:** The first milk of the mother has immune power against diseases.

**Moderator:** Therefore, breastfeeding is important.

**Participant 2:** Yes.

**Participant 6:** Breastfeeding is very important for the baby.

**Moderator:** It is important for the health only or are there any other benefits?

**All Participants:** Health only.

**Participant 6:** If breastfed, the baby will be fine.

**Participant 2:** If we give top milk, it is of no use at all.

**Participant 4:** If we stop breastfeeding and start top milk, the babies will have problem in their growth.

1. If you think breastfeeding is not good, what would be the reasons?

**Moderator:** Sometimes a mother cannot breastfeed her baby. Can you tell any reasons for that?

**Participant 4:** Nothing like that, there is no reason for that. We need to make time for breastfeeding the baby. We need to breastfeed the babies every 5 or 10 minutes so that the baby will not have dryness of mouth.

**Moderator:** Listen to me, there may be some reasons for not breastfeeding. There are some circumstances where a mother cannot breastfeed her baby. Have you noticed any such circumstances?

**Participant 8:** I had no breast milk after delivery.

**Moderator:** Now, are you having breast milk?

**Participant 8:** Now, I am having some breast milk but is not sufficient for the baby.

**Moderator:** She told that she had no breast milk. Any such reasons?

**Participant 2:** Soon after birth my second child had diarrhoea. It was thought that there was something wrong with the breast milk. Then the child was taken to the children’s hospital for treatment. After the diarrhoea stopped, I started breastfeeding.

**Moderator:** At any time, have you thought that the breast milk was not good for the baby?

**All Participants:** No, nothing like that.

**Moderator:** All of you are of the opinion that the breast milk is important?

**All Participants:** Yes.

1. How long does the mother need to breast feed exclusively and the total duration?

**Moderator:** How long the mother should breastfeed exclusively without feeding anything else?

**Participant 1:** Nine months.

**Moderator:** What do you say? (To number 2)

**Participant 2:** Six months.

**Moderator:** Number 3.

**Participant 3:** Six months.

**Moderator:** You, number 4.

**Participant 4:** Six months.

**Moderator:** Not only about you, tell me your opinion about others also. For how many months, other children also should be breast fed exclusively?

**Participant 4:** Other children also should be breast fed for six months or nine months.

**Moderator:** You. (To number 5)

**Participant 5:** Till the children are able to take top feed.

**Moderator:** For how many months the baby should be breast fed exclusively?

**Participant 5:** Up to one year.

**Moderator:** You (To number 6)

**Participant 6:** Up to one year.

**Moderator:** Listen my question carefully, for how many months, only the breast milk and nothing else should be fed to the baby?

**Participant 7:** I have breast fed my child up to six months. There after I started top milk.

**Moderator:** For how many months you think you should breast feed?

**Participant 8:** Six months.

**Moderator:** Along with top feed, do you also breast feed after six months? For how many years you breastfeed them along with top feed?

**Participant 8:** One year.

**Participant 7:** One year.

**Participant 6:** My daughter is about to complete two years and she is still continues breast feeding.

**Moderator:** For how many years you think you should breastfeed her?

**Participant 6:** I am going to discontinue.

**Moderator:** According to your opinion, for how long you are going to breastfeed?

**Participant 6:** I am going to discontinue.

**Moderator:** No, it is not about good or bad. For how many years you think the breast feeding should be done?

**Participant 6:** One year.

**Participant 5:** I have breast fed up to two years.

**Participant 4:** Even if the breast feeding is done up to three years it is good only.

**Participant 3:** I have two children of twenty months old and I want to breast feed them till three years.

**Participant 2:** One and half year.

**Participant 1:** One year.

**Breastfeeding social norm**

1. Do all of the mothers in the community breastfeed?

**All Participants agreed for this.**

1. Who approves breastfeeding around you?

**All Participants:** All in the community say that, baby should be breastfed.

**Moderator:** Does anybody want to take permission from somebody in the family?

**All Participants:** No, no not necessary to take permission.

**Moderator:** In the family??

**Participant 3:** Even if there is work to do in the family, we are told to breastfeed the baby first and thereafter to do the work.

**Participant 2:** Even if the child is sleeping, we need to wake it up and breastfeed.

**Moderator:** Is there any different opinion in the family about breastfeeding? Whether to breastfeed or not?

**All Participants:** Nothing like that.

**Moderator:** All of you agree that, you don’t need permission to breastfeed?

**All Participants:** Yes. (Strongly agreed)

Moderator: After birth, when the mother should start breastfeeding?

**Participant** **8:** When the baby is given to us after delivery, we should start breastfeeding.

**Participant 7:** I got breast milk secretion three days after delivery. I have breastfed after three days. Till then I fed the baby with cow milk.

**Participant 6:** After half an hour of delivery, I started breastfeeding.

**Participant 5:** I too breastfed the baby immediately after birth.

**Participant 4:** My child was admitted in the ICU and the baby used to be given to me for breastfeeding now and then and I used to breast feed.

**Participant 3:** I used to express the breast milk manually and feed the baby as the baby was admitted in NICU.

**Participant 2:** After delivery, I was shifted to the ward and there after I started breastfeeding.

**Moderator:** How much time it took?

**Participant 2:** It took 10 to 15 minutes.

**Participant 1:** After my delivery, the baby was not given to me immediately. Therefore, sugar water was fed to the baby as I had no milk in my breast at that time.

1. Who disapproves breastfeeding around you?

**All Participants:** No one disapproves. (All strongly agreed that nobody in the community disagrees breastfeeding)

1. What do you and people around you think about prelacteal feeds?

**Participant 3:** They give honey.

**Participant 4:** I have not given any prelacteal feeds.

**Participant 4:** Powder milk.

**Moderator:** You need to tell about you and about the people around you in the community.

**Participant 4:** Other people in the community feed Ragi porridge and coconut water.

**(**Moderator insisted the participants to tell about prelacteal feeds.)

**Participant 4:** Honey.

**Participant 5:** Sugar water.

**Participant 6:** Honey and sugar water.

**Participant 7:** We fed cow milk. Because it is, thin.

**Moderator:** Did you give thick cow milk or you added water to it?

**Participant 7:** We gave thick cow milk only. (She meant plain milk without adding water to it)

**Moderator:** How did you feed cow milk?

**Participant 7:** With spoon.

**Participant 8:** I fed powder milk.

**Moderator:** Who prescribed powder milk?

**Participant 8:** There in the hospital only. (She meant the doctor in the hospital)

**Moderator:** Do people in your village feed anything else? In your villages, you might have seen them.

**Participant 5:** I don’t know.

**Participant 4:** Gutti…

**Moderator:** Do they feed it before starting breast milk?

**Participant 4:** Before feeding breast milk.

**Participant 5 (Intercepts):** They give it to clean up the stomach.

**Moderator:** Is it fed before starting the breastfeeding or after?

**Participant 4 & 5 (Together):** Yes, after starting breastfeeding.

**Moderator:** But what I am asking is; before starting breastfeeding. Apart from honey, sugar water and cow milk, what else they feed?

**Participant 4 & 5 (Together):** Nothing.

1. What do you and people around you think about colostrum?

**Moderator:** The first milk, which we call it as cheese milk or colostrum should it be given or not?

**All Participants (In chorus):** Yes. It should be given.

**Moderator:** Why, it should be given?

**Participant 8:** It is good for baby.

**Participant 7:** Doctors say it is good and should be fed to baby. We also feel good to feed colostrum to baby.

**Participant 6:** I also feel good.

**Moderator:** Why it is good?

**Participant 6:** It is good for the baby, if fed with colostrum.

**Participant 5:** If the baby is to grow and to be healthy, the first milk should be fed.

**Participant 4:** The baby should be fed with the first milk or the cheese milk (colostrum).

**Moderator:** What are its advantages?

**Participant 4:** It is good for babies; they will not have dryness of mouth.

**Participant 4 & 5 (Together):** Babies will not have any diseases.

**Participant 3:** It contains protein in the first milk. It is good for the baby if it is fed.

**Participant 2:** It contains immune power against disease and protein; it contains all the ingredients necessary for the baby’s growth that is why it should be given.

**Participant 1:** It contains all the ingredients necessary for the baby’s growth, therefore it should be fed.

1. What do you and people around you think about exclusive breastfeeding?

**Participant 3:** People scold us to feed baby with breast milk saying, “Do you buy breast milk? Then why don’t you better feed it to your child”.

**Moderator:** Do they ask you to feed it compulsorily?

**Participant 3:** Yes.

**Participant 1:** The family members say that if that is fed the baby will have faty abdomen and will become fat.

**Moderator:** Do you mean to say that it happens if top milk is fed.

**Participant 1:** Yes.

**Moderator:** Do they say to breastfeed?

**Participant 1:** Yes, they tell us to breast feed and not any top food.

**Participant 2:** We also say to breastfed.

**Moderator:** Tell me about people in your village, in your family, in your relatives what do they say about exclusive breastfeeding?

**Participant 2:** They tell us to breast feed and advise us to give little water to the babies in the afternoon.

**Moderator:** Why do they ask you to feed water?

**Participant 2:** Because the baby will have dry mouth, therefore they tell us to give water.

**Moderator:** How many of you give water to the babies within six months?

**Participants:** (7 participants raised their hands)

**(Moderator verified with the participants about giving water in the afternoon during summer. They felt that it is because of heat in the summer and baby needs to be hydrated**)

**Participant 2:** Yes, there will be heat in the summer.

**Participant 4:** The baby will have good urination. There will no problem to the baby.

**Participant 5:** For urination.

**Participant 6:** Because, the baby’s throat will be dry.

**Moderator:** For what other reason you feed top milk?

**All Participants:** No. (They meant that they did not feed top milk for any other reason)

**Participant 4:** Other people say that breast milk should be fed. The baby will have strong bone.

**Participant 5:** People say, “For the good growth of the babies, breast milk should be fed.”

**Moderator:** All people’s opinion is to breastfeed the baby.

**Participant 5:** Yes. Should be fed.

**Participant 6:** In our house, the family members tell me to breastfeed. People around us tell me…… “The child is grown up now, feed some top food.”

**Moderator:** They may be telling to top feed now, but what about during the first six months?

**Participant 5:** Majority people in the villages encourage us to breastfeed. (She meant during the first six months)

**Participant 7:** I was thinking that I had no breast milk. But they told me that even though there is no breast milk the baby’s jaundice will disappear if you breastfeed the baby.

**Moderator:** So, majority people encourage breastfeeding.

**Participant 5:** Yes, they encourage us. (All participants agree that the community people around them encourage them to breastfeed.

1. What do you and people around you think of gripewater /gutti /any medicines during the first six months?

**Moderator:** Does anybody give anything extra food during the first six months of exclusive breastfeeding?

**All Participants:** Yes.

**Moderator:** What do they give?

**Participant 8:** I didn’t have breast milk, therefore I fed goat milk to my baby.

**Participant 7:** I gave cow milk and rice porridge.

**Moderator:** When did you give it? At which month?

**Participant 7:** At third month.

**Moderator:** What was the reason?

**Participant 7:** I had no breast milk.

**Participant 6:** I had breast milk. I didn’t give anything else.

**Moderator:** Do people around you in your village give anything other than breast milk? **Participant 2:** Some people crush the banana and feed it to baby. And they give rice porridge.

**Participant 6:** My aunty gave sweet Semolina porridge to the baby.

**Moderator:** At what month?

**Participant 6:** They have fed within four months.

**Participant 5:** My sister had no breast milk. Her doctor had prescribed milk powder. She used to mix the powder milk and the cow milk and used to feed it to her baby.

**Participant 4:** The Gutti should be fed after one and half months of delivery. Gutti is prepared by dry dates, almond should be soaked in hot water, then its coting is removed after that they are rubbed on water washed and clean stone. Then it is mixed in the breast milk and fed to the baby. By feeding this, the babies will have good body weight and they will be fine.

**Participant 3:** We soak it in breast milk only and there after they are rubbed on a clean stone and feed it with spoon.

**Participant 2:** I had readymade bottles of Gutti and gripe water and I fed mixing both of them.

**Moderator:** Gutti and gripe water are different; did you feed both of them?

**Participant 2:** Yes.

**Moderator:** Do they give gripe water?

**Participant 2:** Yes.

**Moderator:** How many of you have given grip water? Raise your hands.

**(Three participants raised their hands)**

**Moderator:** Did you feed? (To Number 4)

**Participant 4:** No, I have given gutti.

**Moderator:** How many of you have given gutti?

**(Six out of eight participants raised their hands)**

**Moderator:** What else they feed to increase the body weight of the baby and to make the baby healthy?

**Participant 3:** Finger millets are roasted and made as flour by grinding in with the help of a grinding stone (available at home) and then it is prepared as a thin porridge and fed.

**Moderator:** At which month it is given to baby?

**Participant 3:** At the third month.

**Participant 4:** For the babies of five months Sari is given. This sari is prepared by adding five types of Serials, Rice, Black gram, Finger millet, Jowar and wheat. All these are washed, cleaned and dried. Then their flour is mixed in hot water that is called sari. It is fed to babies. The children will become healthy.

**Moderator:** Apart from these, are there any medicines prescribed to babies?

**Participant 3:** My children were very small and the doctors in tertiary care level hospital had prescribed tonics for them. And we had given those only, now the babies are fine.

**Moderator:** How do you feed the milk powder to the baby?

**Participant 4:** It is mixed in warm water and fed.

**Moderator:** Do you feed it with spoon?

**Participant 4:** Yes.

1. Anything else you would like to add that you think is important?

**Participant 7:** If we don’t feed the baby the breasts get engorged. People ask me, “why your child has become weak? Breastfeed the baby”

**Participant 4:** Sometimes there may be swelling in the breast due to blockage of breast milk. And we may get fever. Therefore, we need to go to hospital and consult. In the hospital they treat us with the injection and tablet and tell us to express the breast milk and to throw it away. It is safer to throw away a couple of spoonful of breast milk and clean the breast by wiping and feed the baby.

**Breastfeeding perceived control**

1. What makes breastfeeding easier?

**Participant 2:** Doctor say to us “You feed the baby whatever quantity it takes and If you still feel uncomfortable, express the remaining breast milk and throw it away”.

**Participant 1:** The babies feed whatever quantity of breastmilk they want and then the remaining breast milk should be expressed and thrown away.

**Participant 4:** Instead of wasting the remaining breast milk after feeding our children, the leftover breast milk can be given to babies of other mothers who do not have breast milk.

**Participant 5:** When we finish feeding our children, the remaining breast milk can be given to other children. Immediately after birth the babies are small and they are unable to suck. In that situation, breastfeeding is a little difficult and the mother feels the breasts a little heavy.

**Moderator:** In that case, what should be done to make breastfeeding easier?

**Moderator 2:** When you feel breastfeeding is easy?

**Participant 5:** When we breastfeed with a gap of half an hour or one hour it becomes easy for us.

**Participant 6:** If we breastfeed the baby frequently, it is easy.

**Moderator:** Is there any other reason that makes breastfeeding easy?

**Participant 8:** It becomes easy if we breastfeed daily.

**Participant 7:** In our family, my sister is there, she feeds her baby whatever it feeds and throws away the remaining breastmilk. She has more breast milk. My child does not feed her milk. Therefore, she throws away remaining milk.

**Participant 2:** What doctor says, “Whether it is single delivery or twin delivery, there will be a sufficient quantity of milk in the mother to feed even two more children.” But mothers refuse to feed their children by other mothers. They have such superstition that it is not good for baby to feed other mother’s breast milk. People say, “Leave it, why we should take our child to other mothers for feeding?”

**(Even Participant 3 agreed for the same)**

**Moderator:** Do you have any other opinion about breastfeeding? Anything else do you want to say?

**Participant 5:** If the baby breastfeeds the baby will be healthy and the mother also will be fine.

**Moderator:** Apart from what you have told already, is there anything else?

**Participant 3:** My sister’s child is not willing to breastfeed. It wants top food and if anybody in our family starts eating food the child comes and sits in front, asking to feed the same food what they are eating. But it gets diarrhoea if eats top food and it gets diarrhoea even if it is breastfed. We have taken it to hospital and treated; but it is not controlled.

**Moderator:** So according to your opinion, it is due to breastfeeding?

**Participant 3:** Yes, it is due to breastfeeding.

**Participant 2:** It also depends on what the mother eats. If we eat spicy food, the baby will not be having the digesting power. As for as possible we need to eat bland diet and milk etc.

**Moderator:** Do you want to say that the food which the mother eats will have effect on her breastfeeding?

**Participant 2:** Yes.

**Participant 3:** If baby is fed on top milk its intestine become delicate. In that case if the baby is fed with breast milk then it will not digest and may have diarrhoea.

**Moderator:** Do you think the food what mother takes has any impact on breastfeeding?

**Participant 4:** The mother should not eat anything which is hard and solid, otherwise later the baby will have colicky pain when breastfed.

**Participant 5:** If the mother takes good food, the baby will get good immunity.

**Moderator:** But what she said, that the mother should abstain from some food items. What is your opinion?

**Participant 4:** The baby may not be having the digestive power. It is still a small and young baby.

**Moderator:** But the mother will be taking food does it have effect on the baby?

**Majority of Participants:** Yes. It will effect on breastfeeding.

**Participant 8:** The mother should take bland diet.

**Participant 7:** Yes, the mother should have bland diet.

**Participant 1:** People say that “If a male baby is born, the mother should not drink more water”.

**Moderator:** What they are giving to you now? Yours is male child or a female child?

**Participant 1:** Male child.

**Moderator:** Do they give you enough water to drink?

**Participant 1:** They give only one glass. (All participants laugh…) They tell me, “If you eat spicy food the child gets diarrhoea and will have colicky pain”. And they also tell me not to drink more water. Male children will have colicky pain, if we drink more water. Therefore, elders give us only one glass of water at a time.

**Participant 3:** They do not allow us to eat Jowar (Sorghum) roti.

**Moderator:** They do not give you roti, spicy food and water. But what they give you?

**Participant 3:** They give us rice, sweet semolina porridge and dry coconut ladus.

**Moderator:** For how many months they give you these things?

**Participant 3:** For five months.

**Participant 4:** They give sweet vermicelli (Noodles).

**Moderator:** What they tell you not to eat?

**Participant 4:** Fresh Roti and green vegetables.

**Participant 7:** They give us raw egg and meat.

**Participant 2:** I am a non-vegetarian. I eat meat. One should take egg early in the morning. If we take Alavi (Garden Cress), it is good for bones.

1. What makes breastfeeding harder?

**Participant 3:** My first child had diarrhoea initially, he was one month old. I could not breastfeed him. My family members told me not to breastfeed because the baby had diarrhoea. They fed the baby with top feeds and I developed breast swelling.

**Moderator:** Can you feed the baby even if there is swelling in the breast.

**Participant 4:** Yes, we can breast feed.

**Participant 2:** But it is difficult to feed breast milk.

**Moderator:** What I am asking is in which situation the breastfeeding is difficult?

**Participant 4:** Immediately after delivery, one cannot sit up. At that time if somebody helps us to sit up, we can breastfeed.

**Participant 1:** immediately after delivery I was unable to sit and feed the baby.

**Moderator:** In which other situation it was difficult for you to breastfeed?

**Participant 1:** When I had wound on my breast.

**Moderator:** What had happened to your breast?

**Participant 1:** There was a crack in my nipple with pus and blood coming from it. They gave me arrack (local liquor) to apply on it.

**Participant 2:** For the first child it was little difficult for me to breastfeed her. They had told me to massage the breast and the nipple while taking bath. But it took a little time for the nipple to open. At that time, it was a little difficult for me to breastfeed.

**Participant 3:** At the time of my first child birth the baby had diarrhoea and I could not breastfeed the baby.

**Moderator:** Due to non-feeding of breast milk you had engorgement of the breast.

**Participant 3:** Yes.

**Participant 5:** My first delivery was caesarean and there was pain in the abdomen, therefore at that time it was difficult for me to breastfeed.

**Participant 6:** Mine was normal delivery and I had no difficulty in breastfeeding.

**Participant 2:** There is a woman in my neighbourhood. She had repeated abortions, now she has delivered a baby recently. When she had abortions she had not thrown away her breast milk. Now she is having swelling in the breast.

**Participant 6:** I don’t have anything to say.

**Participant 5:** My sister had delivered; she didn’t have breast milk secretion. That child had problem.

**Participant 2:** For that woman they had told to takeout the breast milk with the help of a pump. Due to repeated use of the pump her nipple was torn. Then the doctors told her “Don’t you have that much common sense; whether the child survives or not in the previous abortions, it was your duty to express the breast milk and throw it away”

**Participant 7:** My delivery was a caesarean; I had to breastfeed the baby in lying down position only.

**Moderator:** Have you seen anybody else with problem of breastfeeding due to any reason.

**Participant 6:** There is a condition called Maletodaku (Stiffness and pain in the breast) it is relieved by hot water pouring on it.

**Participant 2:** But the doctors insist that we should breastfeed in sitting position only even though we have difficulty. And they stand in front of us until we sit up and feed the baby.

**Participant 7:** I had breast fed my baby in lying down position for one day only. Afterwards I started breastfeeding in sitting position under supervision of doctors.

**Participant 8:** I didn’t breastfeed the baby on the day of delivery, there was no breast milk secretion in my breast.

**Moderator:** What other difficulty you had in breastfeeding?

**Participant 8:** I had no breast milk and also retracted nipple.

**Moderator:** Anything else?

**Participant 5:** The more the baby breastfeeds, the breast become relaxed. Otherwise there will be a problem.

**Moderator:** So, according to your opinion, breastfeeding should be started as early as possible and continue breastfeeding to avoid any problem.

**Participant 2:** If we delay in breastfeeding, the breasts becomes full and tight and it is painful for us.

**Participant 5:** The breast milk starts oozing by itself.

1. What does your community think of roles played by maternal depression on breastfeeding?

**Participant 8:** When there is no milk in the breast the mother may have mental tension to breastfeed the baby.

**Participant 7:** Some mothers when they undergo caesarean, they may not have breast milk, at that time they may have depression.

**Participant 4:** Some people pressurise the mother to breast feed repeatedly so they may have depression.

**Moderator:** Listen to my question. It is entirely different question. Sometimes some incidents which the mother does not like may happen within the family or in surrounding community. If the mother gets mentally depressed in relation to those incidents, does it have any effect on her breastfeeding? How many of you say it does happen?

**Majority of Participants agree for this.**

**Moderator:** What effect does it have?

**Participant 1:** If we worry too much about something and get depressed, it will adversely affect baby’s behaviour and breastfeeding.

**Moderator:** If the mother breastfeeds her child when she is mentally depressed what happens? Breastfeeding is easy or not at that time?

**Participant 1:** It will be ok.

**Moderator:** Can you get breast milk properly?

**Participant 1:** Yes.

**Participant 2:** As far as I am concerned, there will not be any problem.

**Moderator:** When the mother is mentally depressed what happens to her breastfeeding.

**Participant 4:** It does not matter.

**Moderator:** In a family they are common; problems are bound to be there. At that time if she gets mentally depressed people say “Her mind has gone mad”. In such condition does it have any effect on breastfeeding?

**Participant 4:** Mental depression is not related to mother’s milk….

**Moderator 2:** It is not about you in some family a mother may have mental depression like she might have gone very silent, doesn’t speak at all even if somebody speaks to her she doesn’t respond in that situation is she able to give proper attention towards breastfeeding her baby?

**Participant 5:** If mother is healthy and breastfeeds her child it will not have any effect. If she is not well and has mental depression, she may have less breast milk secretion and it will have less immunity to the child.

**Moderator:** So, according to you if the mother is not well, it will have effect on her breastfeeding.

**Participant 5:** Yes.

**Participant 7:** She will have the effect on her breast milk

**Moderator:** Not that, mothers mental….

**Participant 8:** If somebody comments something to us, it effects our mind and we stop taking food. Then we may have less milk.

**Participant 3:** She may not be in her right mind; she may not be knowing when to breastfeed her child. Like this….

**Participant 5:** Due to her excessive mental worry there will be less protein in her body and in turn it will affect breastfeeding.

1. What does your community think of roles played by maternal chewing of tobacco on breastfeeding?

**All Participants:** Yes, we have seen.

**Moderator:** All of you have seen.

**All Participants (in chorus):** Yes.

**Participant 1:** My mother in laws sister eats tobacco.

**Moderator:** I am asking you about breastfeeding mothers.

**Participant 2:** Madam, I told you earlier about a woman with swelling in the breast, she used to chew tobacco and gutaka (Tobacco mixed with lime and areca nut). The doctors told her that her breast swelling was due to tobacco only. It has effect on the occurrence of breast swelling and abortions.

**Moderator:** Do you mean to say that it has effect on breastfeeding?

**Participant 2:** It will have effect on breastfeeding. The baby gets our milk; if we take anything it will go into our body and then to baby through breast milk.

**Participant 3:** Mothers chew tobacco. But I have not seen anything bad happening to them.

**Participant 4:** If she breastfeeds, the breast milk goes into baby’s body and it will be the problem to the baby.

**Moderator:** What problem will happen to the baby?

**Participant 4:** If the baby takes breast milk it will have effect on its brain and it may have effect on its future life.

**Participant 5:** It will have effect on the baby’s growth.

**Participant 6:** Nobody chews tobacco; they eat areca nut and betel leaves.

**Moderator:** Do they add tobacco to the areca nut and betel leaves?

**Participant 6:** No.

**Participant 5:** One woman did not have children, she was eating tobacco with areca nut and leaves. She had one girl child there after even for 7 – 8 years she did not conceive. They consulted doctor and they got children after stopping tobacco chewing.

**Participant 7:** People say that by chewing tobacco breast milk burns out. Therefore, the mother will not secret breast milk.

**Participant 8:** They chew areca nut and betel leaves.

**Moderator:** So, it means majority of the breastfeeding women chew tobacco.

**All Participant:** Yes, they chew.

1. What does your community think of roles played by maternal drinking on breastfeeding?

**Moderator:** Do the breastfeeding mothers drink alcohol in your community?

**All Participants (In chorus):** Yes, it is very common.

**Participant 4:** Alcohol is given to the mother after delivery.

**Moderator:** What type of alcohol?

**Participant 4:** That what they call it as whisky.

**Moderator:** Why they give it?

**Participant 4:** They give it for the relief of pain. There may be sutures applied during delivery. They think that by taking alcohol the sutures get dissolved early.

**Moderator:** For how many days it is given?

**Participant 4:** They give it for one and half month or two and half month.

**Moderator:** Daily?

**Participant 4:** Yes, they give it daily.

**Participant 5:** I have seen it giving till the pain subsides.

**Participant 1:** No, they had given it to me to apply to the wound. Locally prepared alcohol.

**Participant 2:** I have not seen any mother taking alcohol.

**Moderator:** What effect will it have? Is it good or bad?

**Participant 4:** It is good.

**Participant 1:** People say that it is good. But I don’t know anything about it.

**Participant 2:** It is not good. Due to alcoholism, one may get cancer or anything. It is not good.

**Participant 4:** Alcohol is good for smooth delivery.

**Moderator:** Does it have effect on breastfeeding?

**Majority Participants (In chorus):** Yes, it has effect.

**Moderator:** How many of you say it has effect?

**(All participants raised hands)**

1. What does your community think of roles played by maternal drug use on breastfeeding?

**Moderator:** Apart from alcohol if the breastfeeding mother takes drugs like ganja (canabbis) or other drugs, have you seen such mothers taking drugs?

**All Participants in chorus denied the fact**.

**Moderator:** Have you heard about it?

**Participant 4:** Yes, I have heard when I had delivered my first child. In that hospital one mother was giving drugs to her own daughter who had delivered. It was shown in a news channel also.

**Moderator:** When they take these drugs, does it have any effect on breastfeeding?

**Participant 4:** Yes, it does have effect. The child may not survive.

1. What does your community think of roles played by maternal anaemia on breastfeeding?

**Participant 4:** No, there is no relation to it.

**Participant 2:** It will not affect.

**Participant 3:** It will have an effect. I had anaemia and my children also had anaemia. My children got blood transfusion in the government tertiary care hospital. Now they are better.

**Participant 4:** I also delivered in the town. At that time, I had anaemia. But the baby had enough blood. So it is not related. The mother can breastfeed even if she is anaemic.

**Participant 5:** Yes, the mother can breastfeed her baby.

**Moderator:** Even though there is no problem in breastfeeding, is there any other effect on the breast milk?

**Participant 4:** It is depending on the food she takes.

**Participant 5:** It depends upon the health of the baby.

**Participant 2:** The mother can breastfeed.

**Participant 5:** If there is any health problem to the mother, it will occur to the baby also. If the baby is healthy and if there is no problem of blood, there will be no effect on her breastfeeding.

**Moderator (Repeated the question):** I am not asking whether to breastfeed or not, is there any effect on breastfeeding?

**All Participants (in chorus):** No effect.

1. What does your community think of roles played by maternal overweight or obesity on breastfeeding?

**All Participants (In chorus):** It does not have any effect.

1. What does your community think of roles played by intimate partner violence on breastfeeding?

**Moderator:** There will be quarrel in the family; there may be assault on the body of women. Such assaults might have occurred on breastfeeding mothers also. Do such incidents happen? Exploitation, what I mean that might have happened.

**All Participants:** Yes, they occur.

**Participant 4:** I tell the example of my sister. Her husband was a drinker. He used to beat and ask her to work instead of breastfeeding; so that he can have money for his bad habit of drinking.

**Moderator:** Does it have effect on breastfeeding?

**Participant 4:** He has beaten her while breastfeeding and she had mental shock due to it. Now I am taking care of her children.

**Participant 5:** Yes, it does occur.

**Moderator:** Have you seen breastfeeding mother being exploited? Beating them physically. Do such things happen?

**Majority Participants (in chorus):** Yes, we have seen such things happening.

**Moderator:** Whether it has an effect on mother’s breastfeeding?

**Participant 5:** There will be effect on the health of the women and on their breastfeeding. **Participant 2:** Yes, it does have effect.

**Participant 3:** There are drinkers in the society. If such persons are there, there will be its effect.

**Participant 2:** There is a saying “Between the fight of husband and wife, the baby suffers”.

**Participant 6:** Yes, it will have effect.

1. ***Acceptability of breastfeeding peer counselors (10 minutes)***
2. Where do you get information about breastfeeding?

**Moderator:** Who gave you information about breastfeeding? You tell (To number 8)

**Participant 8:** My mother.

**Participant 7:** My mother gives. Grandmother gives. Doctors also give the information.

**Participant 6:** My mother gives. Grandmother gives. ASHAs also tell us.

**Participant 5:** Doctor, family members and grandmother.

**Participant 4:** Grandmother.

**Participant 3:** In my family my mother in law told me. After that ASHAs and staff nurses.

**Participant 2:** ASHAs and my mother.

**Participant 1:** ASHAs, my mother and doctor.

1. If you have any breast feeding problem whom do you approach?

**Participant 1:** Doctor

**Moderator:** When a woman has a problem in breastfeeding, whom does she approach?

**All Participants (In chorus):** Doctor.

1. Are you aware of any programmes by the government to promote breast feeding practices in the country?

**Moderator:** Now, I ask you, do you have any information about the government programmes about breastfeeding? What are there about breastfeeding? Are you having any information regarding that?

**Participant 2:** Here only, once a meeting was conducted at PHC. Before my child was born, there was a meeting done here about the child health.

**Moderator:** You know only about the meeting done here. But do you know about any programmes regarding breastfeeding.

**All Participants (In chorus):** We don’t know.

1. Is there any support of the women from the village/community?

**Moderator:** In your community, if there is any problem in breastfeeding, is there anybody to help you? Apart from ASHA, ANM and Nurses, is there anybody else to help you in breastfeeding in your community?

**Participant 2:** My mother only.

**Participant 1:** My mother.

**Moderator:** Anybody else? Elders in the community, grandmothers?

**Participant 5:** Elders in the community come to our help.

**Moderator:** Are there elders in the community to help you?

**Participant 5:** Yes, they are there. Grandmothers also tell us.

**Moderator:** What do you say? You had small babies. Was there anybody to help you?

**Participant 3:** My mother in law was there to help me.

**Participant 2:** In our village, people use a lot of traditional medicines. If a mother doesn’t have breast milk early, people tell us to have some kind of medicinal herb rubbed and applied to the chest. They say, by doing so, one will have breast milk.

**Participant 4:** My mother.

**Participant 5:** Elders give us suggestions.

**Moderator:** What do you mean by elders?

**Participant 5:** My mother.

**Participant 7:** Grandmother and mother.

**Moderator:** If you have any problem in breastfeeding, who helped you? (To participant 8)

**Participant 8:** My mother.

**Moderator:** Are there anybody else in your community who come and help you?

**Participant 8:** Yes, there are.

**Moderator:** Not only for you; if any other mother has a problem, do they come and help?

**Participant 8:** Yes, they do.

**Moderator:** Do you mean to say that such person should be there in your community? Or not?

**All Participants (In chorus):** Such person should be there.

**Participant 5:** In the villages, there are persons called as sulagitti or dayi (Traditional birth attenders) they also help us.

**Moderator:** So, all of your opinion is that, such a person should be there in the community. Apart from your mothers, one person should be there.

**All Participants (In chorus):** Yes.

**Participant 5:** At the time of delivery, if there is any problem to the babies in breastfeeding they will be there for the help.

**Moderator:** There are dais and elders in the family. Your opinion is that such persons should be there.

**Participant 5:** Yes.

1. **Is she trained in giving breast feeding support and help?**

Question not asked.

**Breastfeeding peer attitude**

1. **Do you think that a person (Peer Counsellor) from your community/village, if trained will be helpful to solve the breast-feeding problems?**

**And**

**9. What would be the benefit to have Peer Counsellor?**

**Moderator:** If such a person is trained, correctly about breastfeeding, demonstrating how to breastfeed is it going to help?

**Participant 1:** Such person should be there and persons like you (she meant doctors) should train them.

**Moderator:** Does training of such persons is going to help?

**All Participants (In chorus):** Yes.

**Participant 5:** In villages, sometimes-such facilities may not be there, bus facility may not there and even the doctors may not be available there. In such situation if there is a peer counsellor to solve routine problems. It would be good.

**Moderator:** Can you identify such a person in your village?

**Participant 2:** If we tell to ASHAs, they will do something about it.

**Moderator:** Forget about ASHAs. I am asking your opinion about peer counsellor?

**Participant 4:** People in the neighbourhood come and help.

**Moderator:** They are not trained. What I am asking is, if such persons are trained and made to work.

**Participant 5:** If such person is there, it will help. It should help.

**Moderator:** How will it help you?

**Participant 5:** They would have got training and would be having all the knowledge. If there is any problem to the baby or us, if they come immediately, they will advise us. If they could not solve the problem, they would refer to a doctor in the city or to any known person and see that the problem does not get aggravated.

**Participant 4:** If we do not know something, they will explain to us and we understand.

**Moderator:** The person about whom I am telling you now, if that person is from your own village and if training is given to them and asked to help the breast feeding mothers, will it be useful?

**All Participants:** Yes, it will be useful.

**Moderator:** You are all saying that it will be useful. Can you identify such person from your respective villages who can be trained?

**All Participants (In chorus):** Yes, training can be given to them.

**Moderator:** Are such persons available?

**All Participants (In chorus):** Yes**.**

**Participant:** If the baby is not well or the mother herself gets some problem, in that situation of stress, what to do…

**Moderator:** Some people will not be able to understand what to do in such situation. If a trained person who can advise and give treatment, it will be good?

**Participant 4:** My baby had jaundice and it was admitted in ICU. Second time also it had. I had come to the hospital due to breastfeeding problem. ASHA had brought me. Doctor said that the child had jaundice and it may not survive and told me to take the child to higher hospital immediately. I took the child to higher hospital immediately. To save the child, I took the child to higher hospital and the doctor appreciated for timely action.

**Moderator:** Now, I told you about identifying peer counsellor from your village and training her. Apart from training her if we give her a mobile and in that mobile, if we add information about training to the breastfeeding mother, how to hold the baby, how to breastfeed the baby, so that there will be no problem to the mother. And if this information is given with the help of a mobile, will it be easy or it will be easy if she tells it just orally?

**Participant 5:** It is good if it is shown because if it is told orally it may not be understood properly. They may not understand it, if they are told orally. If they see it themselves, they learn better.

**Moderator:** What do you say? If the information is incorporated in the mobile and given to the breastfeeding mother, will it be useful or not?

**All Participants (In chorus):** It will be useful.

**Moderator:** What information should be there in it?

**Participant 4:** It will be having good messages in it. Looking at the message, we have to understand.

**Participant 5:** By listening and seeing we can understand.

1. **Do you accept receiving Breast feeding counselling and advice from such a person?**

**And**

1. **What should be the qualifications of Peer Counsellor?**

**Moderator:** What should be the qualifications? If is a person of our village, a person who has taken training about breastfeeding; not only about breastfeeding but also about how to breastfeed, when to breastfeed and if there is a problem about if who can help in solving it. If such a person is there in your village, you said that it would be useful. But what should be the qualifications of such a person? What type of person she should be so that it will be useful? I am asking you, to which type of person this training is useful?

**Participant 2:** She should be able to work in any type of situation. Even if there is nobody, she should come and tell the mother at any time. She should have taken proper training to advise us, as we are not having any knowledge about it.

**Participant 3:** She should not scold the mother saying “Can you not do it yourself?” Some persons, even though they are trained, they do not do their work. She should not be such a person. She should have patience and work accordingly.

**Participant 4:** She has been trained by others. If she does not advise us how can we understand? If they advise us, we will be able to know.

**Participant 5:** Even if she has any personal problem, she should leave it and give attention towards the health of the baby.

**Participant 6:** She should be having good characters and she should come if anybody calls her.

**Participant 7:** Some persons do not have good characters and they go on telling other people “See, she does not have milk in her breast.” She should not tell like this. That will be a problem.

**Moderator:** She should not tell such things to others

**Participant 7:** Yes, she should not tell others. If she tells, the baby doesn’t digest the breast milk.

**Participant 4:** They should tell all during pregnancy about retracted nipple, nipple pulling out while taking bath.

**Participant 5:** At the time of first delivery, the mother may not know properly about the breastfeeding. If the peer counsellor is trained in that and in turn, if she tells us about this, it will be useful.

**Moderator:** What should be her educational qualifications? Should she be educated or is it okay if she is not educated?

**Participant 5:** She should be educated.

**Participant 7:** She should be educated. Even if she is not educated, if she tells properly people will understand and they do it.

**Participant 8:** Even though she is less educated she should have more knowledge.

**Participant 6:** If she is not well educated, it is okay.

**Participant 5:** If she is less educated and has more knowledge it is okay.

**Participant 4:** No problem even if she is not well educated she can understand.

**Participant 3:** If she is ready, she should understand and come whenever we call her for help.

**Participant 2:** It is not depending upon her education. It depends upon how she understands it and tells us so that it goes into our head. It is not necessary that she should be well educated. If she has less education, it is enough.

**Moderator:** If such persons are there in your community are they recognised and accepted.

**All Participants:** Yes.

**Moderator:** Do the people accept them?

**All Participants (In chorus):** Yes, people will accept such peer counsellor.

**Breastfeeding peer perceived control**

1. (Already answered)
2. **What would be the barriers/problems to have Peer Counsellor?**

- Not asked this question.

**Breastfeeding peer social norm**

1. **Who approves the presence of Peer Counsellor?**

- Not asked this question.

1. **Who disapproves the presence of Peer Counsellor?**

- Not asked this question.

1. ***Program to train breastfeeding peer counselors (10 minutes)***
2. **How does the health worker help you in breast feeding activities?**

- Not asked this question.

1. **Does the health worker visit and advise you about Breast feeding before delivery?**

- Not asked this question.

1. **What does the health worker talk about breastfeeding antenatally?**

**Moderator:** When you were pregnant what ASHAs and ANMs (Health workers) used to talk about breastfeeding?

**Participant 8:** When there was ANC check-up, they used to come to us to take us to the PHC.

**Moderator:** Where they giving you information about breastfeeding, when they came to you?

**Participant 8:** They didn’t give us any information about breastfeeding.

**Moderator:** When you were pregnant….?

**Participant 8:** Nothing.

**Participant 7:** They had given us incomplete information.

**Participant 6:** They have given the information to me.

**Participant 4 and 5:** Yes, we received the information from health workers.

**Participant 2,3 and 5:** We received breastfeeding information from health worker during a meeting held in primary health centre.

**Participant 4:** For me my mother herself has told.

**Moderator:** What did they tell you in the meeting?

**Participant 2:** They told us how to be clean during pregnancy and how to clean our breasts while taking bath. The pregnant women would not have had retracted nipple problem if they had listened to this advice.

**The child of participant 5 started to cry outside and it was brought inside to its mother.**

1. **What happens at birth? Is she present at birth and help you with early starting of Breast Feeding?**

**Moderator:** Immediately after birth who tells you about breastfeeding? what happens at birth?

**Participant 2:** My mother and the doctor told me about breastfeeding.

**Participant 8:** My mother

**Participant 7:** My mother and grandmother told me about breastfeeding.

**Participant 6:** My grandmother told about breastfeeding.

**Participant 5:** My mother and the nurse told me.

1. **How frequently does she visit you after birth of the baby?**

**Moderator:** After delivery, when you go home does anybody come to your house?

**Participant 7 and 5:** Yes.

**Participant 8:** Yes, they come.

**Moderator:** Who comes?

**Participant 8:** Dais or ASHAs come.

**Moderator:** How many times they come in a day?

**Participant 4:** They come twice in a day.

**Moderator:** How many times they come in a month?

**Participant 8:** Once in fortnight.

**Moderator:** Do they ask you anything about breastfeeding when they come to you?

**Participant 8:** They tell us “If you breastfeed the baby it’s weight will increase. Even though the milk does not come give the breast to the baby”.

**Moderator:** Do they observe you, breastfeeding the baby?

**Participant 8:** Yes.

**Participant 5:** Yes, they observe.

**Moderator:** All of you are telling that they examine how you breastfeed your babies. **Participant 2:** There is no ASHA. My mother only takes care of my breastfeeding.

**Moderator:** How many times the health workers come to you? In a week or in a month?

**Participant 4:** They come whenever we call them.

**Moderator:** approximately how many times they come?

**Participant 4:** Whenever there is some problem with my baby.

**Moderator:** By themselves when they come?

**Participant 5:** Once in a week.

**Participant 4:** 4 times in a week.

**Participant 2:** To us, there is nothing like how many times in a week, they come whenever there is date of vaccination to my child.

**Participant 3:** ASHA is near my house only. She comes once in two days.

**Participant 6:** ASHA stays near my house; so she visits frequently.

**Participant 7:** Once in 15 days.

**Participant 5:** If there is any problem, they come to our house and take care of me and my baby.

1. **What is the purpose of visit?**

**Moderator:** Do they give attention towards your breastfeeding and ask you about it if there is any problem?

**All Participants:** Yes, they do.

**Participant 3:** ASHA is next to my house. If there is any injection to be given to us, she comes and tells us saying “Please come there is an injection to be given to you”.

**Moderator:** No, when there is a problem with you, that time does she come and see you?

**Participant 3:** Yes.

1. **How frequently does she assess your breastfeeding practices?**

Question not asked

1. **How many mothers does the health worker visit per day/per month?**

**Participant 4:** Whatever number of mothers are there in the village.

**Moderator:** Approximately.

**Participant 4:** Approximately there are 10 – 15 mothers in the village. In my village there are twenty.

**Moderator:** How many mothers they meet in a day?

**Participant 4:** The mother who have delivered.

**Moderator:** Listen my question. when they come into your village she may be your ANM or ASHA worker. When they come, on that day, how many mothers they meet?

**Participant 4:** 3-4 mothers.

**Participant 6:** Six mothers.

**Participant 5:** Approximately five mothers.

**Participant 6:** 5 to 6 mothers.

**Participant 7:** 8 mothers.

**Participant 5:** They will be having the number of children supposed to examine on that day. And they visit that many mothers and children on that day.

1. **What are the common breast feeding problems you observe in your community?**

- Already answered in section A

1. **Do you think that peer counsellor who is from the same community would help and support mothers?**

- Already answered as yes in previous question

1. **What do you think would be the barriers for Peer Counsellors in the community?**

**Participant 2:** Some people in the community comment… “What is so great about her? What she has learnt and for what she has come into the village?”. Few people make such comments.

**Participant 4:** Some people in the community, are very much interested in commenting on others.

**Participant 6:** People say “Do they give money? Forget whatever they say; why to listen whatever they say?”

**Participant 3:** If such trained person comes to us, it will be helpful to others also.

**Moderator:** Do the people in the community give any trouble to such persons in doing their work?

**Participant 2:** Some people comment negatively about them.

**Participant 3:** There are some bad people. And there are good people also.

**Participant 7:** Some people comment about them behind their back. Due to this some may (She meant peer counsellor) leave their work.

**Participant 7:** If they are explained about it, they do not object.

**Moderator:** Do the people accept them?

**Majority Participants:** Yes.

**Moderator:** Do they give any trouble to them?

**All Participants:** No, they do not give any trouble.

**Participant 5:** If people know that the community and the children get benefitted, they will help.

1. **Will there be future opportunities for these Peer Counsellors to continue in the community?**

**Participant 4 and 5:** Yes, there will be opportunities.

**Moderator:** Even after this study is over, do they get opportunities?

**Participant 4:** Yes, there will be opportunities.

**Moderator:** Do all of you agree to this?

**All Participants (In chorus):** Yes.

**Participant 5:** We have seen such workers earlier. If we encourage them they can work.

1. **What should be the criteria for acceptance of such counsellors by you and the community?**

- Already answered

1. **How do you identify and recruit such counsellors?**

**Moderator:** Can you identify such person in your village? Are they available in your community?

**Participant 8:** Even if they are available, other people in the village are bent upon spoiling the work (tells jokingly)

**Moderator:** In your village? (To number 7)

**Participant 7:** In our village also, they are available but people only comment (All laugh)

**Participant 6:** They are available in our community

**Participant 5:** We may identify such peer counsellor in our community.

**Participant 3:** Some may agree to work. But they think, “what to do after going there and taking that training”.

**Moderator:** If the training is given in their village itself?

**Participant 3:** Yes, then they will agree.

**Participant 5:** Earlier there were no facilities in our villages. Now, ASHAs are a lot of help to us.

**Moderator:** In the same way if a peer counsellor comes into your village?

**Participant 3:** It will be still more useful, if these people (She meant peer counsellor) come into our village.

**Participant 5:** Yes.

**Moderator:** I am asking you once again; how to identify and appoint peer counsellor?

**Participant 2:** You need to come to our village once and ask about it. If they agree, it is okay.

**Moderator:** How they are available to us?

**All Participants (In chorus):** Through ASHAs.

**Participant 2:** If you ask ASHAs, they will help you.

**Participant 4:** If you tell this to ASHAs, they will help you.

**Participant 5:** If you come to Anganwadi along with ASHA and discuss about any person eligible for this work, that way they can be identified and appointed.

**F. Wrap up**

1. ***Ask participants***

**“How did you feel about participating in this session? What was easy? What was hard?”**

**Moderator:** Was the discussion good?

**All Participants (in chorus):** Yes, it was good.

1. ***Summarize and thank participants by saying:***

**“We appreciate all of your great ideas. You have been a big help, and we want to thank you very much for all the information you have shared with us today. We know that your ideas will help to make this a successful program and technology. Thank you once again for your participation – we really appreciate you!”**

**Moderator: Thank you.**

**Focus group interview guide**

**Materials Needed:**

Food and Beverages

Newsprint & Markers

Masking tape

Nametags

Audiotape recorders and tape

Participant incentives

Questionnaires

Pencils

Attendance sheet

**Introductions and Focus Group Process (10 minutes)**

1. *Team members will have nametags on prior to participants entering the focus group venue*
2. *Give participants nametags as they arrive.*
3. *Instruct participants to select celebrity name*
4. *Have participants sign-in on attendance sheet.*
5. *Explain the purpose of the focus group session by saying:*

**“Welcome to today’s focus group. We are planning to develop mobile technology to support breastfeeding peer counselors and breastfeeding mothers. We asked you to come to today’s session because we would like to hear from you about your opinions and ideas on the technology content. You are the experts, and we can learn from you. We need your honest opinion – good and bad – about what we are trying to do. We would like you to share what you think – and what you think other members in your community might think about our project. Everything you are thinking is important to us. There are no right or wrong answers. We value your opinion. We would be very happy if you would help us to make the best technology possible.**

**Please remember to use your celebrity name and refer to others with their celebrity names. Also, try not to use any friends’ names or specific locations. But if you do, we will delete them from the audio recording.**

*As a reminder we are going to turn on the tape recorder now.*

*Is everyone ok with that? TURN TAPE ON: and announce*

“This is focus group # 4 on 28 / 03 / 2018 for the “BEST4Baby”

*1. Ask the participants to introduce themselves by saying their celebrity name and what is your favorite color.*

*2. All members of investigative team introduce themselves.*

**“Now, we would like to develop some group rules so we can learn as much as possible from each other.”**

**Group Rules (10 minutes)**

1. *Develop group rules to protect participants’ confidentiality. Offer examples of group rules if participants are stuck:*

Be respectful

Be honest

Maintain confidentiality

One person speaks at a time

Listen to others

No put downs or insults

Turn off all cell phones

**“Even though we will be talking about breastfeeding and technology development today, we do not require you to talk about your personal experiences with breastfeeding if you do not feel comfortable. You can instead share what you think or others’ experiences. However, please do not use any specific names or identifiable information of others for protecting their privacy. Is that clear to everyone?”**

**BREAST FEEDING EDUCATION SUPPORT TOOL for BABIES**

**Focus Group Discussion guide for Mothers who**

**have successfully breastfed at least one child in the past three years**

**Discussion (40 Minutes)**

**“We are working to develop a mobile technology to support breastfeeding peer counselors and breastfeeding mothers in India. We want to know what you think and what you know about breastfeeding practices. Your inputs will certainly help develop feasible and effective technology to support breastfeeding. We have questions we prepared but we will let our group guide our own discussion. Let’s start with some initial questions about breastfeeding experiences.”**

1. ***Breastfeeding practice and support mechanism (20 minutes)***
2. **Tell us about your breast feeding experience**

**Moderator:** For how long you have breastfed your baby? How many children do you have?

**Participant 1:** This is the first one. Still I am breastfeeding him. Now he is nine months of age.

**Participant 2:** I have two children. For the first child I have breastfed for two years. And for this child I am still breastfeeding and this child is ten months old.

**Participant 3:** I have two children. For the first child I breast fed for one and half years. And this child is nine months old and I am still breastfeeding for it.

**Participant 4:** I have one child of one year old and it is still breastfeeding.

**Participant 5:** I have three children for the first child I breastfed for two years, for the second child one and half years and the third child is still breastfeeding.

**Participant 6:** I have only child of two years old and it is still breastfeeding.

**Participant 7:** I have one child of two years and I breastfed it for two years and now I am pregnant for the last five months and after being pregnant I am not breastfeeding. And to stop breastfeeding I applied medicine to the nipples. So, that the child stops breastfeeding.

**Participant 8:** I am not breastfeeding. This is my first child; she is five months old.

**Moderator:** What do you feed to the child?

**Participant 8:** I give top milk.

**Participant 9:** I have three children. For the first child I breast fed for two years, for the second child one and half years and the third child is eleven months old and it is still breastfeeding.

**Participant 10:** I have four children. For the first child I have breastfed for one and half years, for the second child I breast fed for two years, the third child I breast fed for two years and this child is ten months old and it is still breastfeeding.

**Breastfeeding attitude**

1. **Do you think breast feeding is important for the baby?**

**AND**

1. **Why? What are the benefits?**

**Moderator:** Do you agree that the baby should be breast fed or not?

**All Participants:** Yes, the baby should be breast fed.

**Moderator:** What is the importance of breastfeeding and by breastfeeding what are the benefits to the baby?

**Participant 1:** The babies will become healthy and fine.

**Participant 2:** The baby will be healthy.

**Participant 10:** To make the children strong.

**Participant 9:** Whatever vegetables we eat, the nutrition in it goes to the child through the breast milk and to the child. If we feed the child with top milk it would have only the milk.

**Moderator:** By the nutrients going to the child through the breast milk what will be its use?

**Participant 9:** They will become healthy.

**Participant 8:** To make the child healthy.

**Participant 7:** It will increase the weight of the child and mental development.

**Participant 6:** For the health of the baby.

**Participant 5:** People say that; we should breastfeed the baby within half an hour after delivery. Because mothers milk is good for the baby. Earlier sugar water was used to be given. But now we should feed the baby with breast milk only.

**Participant 9:** By feeding the breast milk that disease to the baby are prevented. (She meant it increases the immunity power of the baby). Therefore, immediately after delivery we are told to breast feed and not to feed anything else.

**Participant 7:** I wanted to tell the same thing what she told. (She meant number 9). If we breastfeed the baby immediately after delivery, the baby will not get any diseases and the baby will have all the nutritious factors.

1. **If you think breastfeeding is not good, what would be the reasons?**

**Participant 10:** Some mothers who are very thin and weak do not breastfeed their baby.

**Participant 6:** Some mothers who do not have breast milk in them they don’t breastfeed their babies. They feed top milk to the babies.

**Participant 8:** I had retracted nipple, so I could not breastfeed my baby.

1. **How long does the mother need to breast feed exclusively and the total duration?**

**Participant 3:** Till 6 months the breastfeeding should be fed exclusively and after that top feed can be given.

**Participant 6:** Up to 3 years’ breast feeding is to be done.

**Participant 1:** Breastfeeding should be done for one year.

**Participant 2:** Up to 2 years to 2 and half years.

**Participant 1:** Up to 1 year.

**Participant 5:** Up to 2 years.

**Participant 6:** Up to 3 years.

**Participant 7:** I breastfed my baby exclusively for 6 months and totally my baby was breastfed for 2 years.

**Participant 8:** Up to 2 years to 2 and half years.

**Participant 9:** As I am also of the same opinion that for two and two and half years the breastfeeding should be done.

**Participant 10:** Up to 2 years.

**Moderator:** According to you for how many months exclusive breastfeeding should be done?

**Participant 1:** 6 Months.

**Participant 2:** 6 Months.

**Participant 3:** 6 Months.

**Participant 4:** 7 Months.

**Participant 5:** 6 Months.

**Participant 6:** 6 Months.

**Participant 7:** 6 Months.

**Participant 8:** 6 Months.

**Participant 9:** 6 Months to 7 Months.

**Participant 10:** 9 Months.

**Moderator:** When did you start top feeding to your baby?

**Participant 1:** After 7 months.

**Participant 2:** After 7 months.

**Participant 3:** After 9 months.

**Participant 4:** After 7 months.

**Participant 5:** After 7 months.

**Participant 6:** After 7 to 8 months.

**Participant 7:** After 8 months.

**Participant 8:** Since birth my baby is fed with top milk.

**Participant 9:** After 10 months.

**Participant 10:** After 9 months.

**Breastfeeding social norm**

1. **Do all of the mothers in the community breastfeed?**

**All Participants (In chorus):** Yes, all the mothers in our community breastfeed their babies.

1. **Who approves breastfeeding around you?**

**All Participants (In chorus):** We breastfeed our babies on our own we don’t need anybody’s approval.

1. **Who disapproves breastfeeding around you?**

**All Participants (In chorus):** No… no…. nobody disapproves for our breastfeeding.

1. **What do you and people around you think about prelacteal feeds?**

**Participant 10:** For my first child I fed sugar water immediately after birth.

**Participant 9:** I fed my baby with breast milk only.

**Participant 8:** I fed my baby goat milk mixed with water.

**Participant 7:** Honey is fed as prelacteal feed.

**Participant 6:** Honey was fed for my baby as there was no milk immediately after delivery.

**Participant 5:** I have breast fed my baby immediately after delivery.

**Participant 4:** Nothing was fed as prelacteal feed for my baby.

**Participant 3:** I have not fed anything as prelacteal feed. I have fed breast milk only.

**Participant 2:** I had given sugar water to my baby.

**Participant 1:** I had fed only breast milk to my baby.

**Moderator:** Why did you give prelacteal feed?

**Participant 10:** On the first day after delivery the mother doesn’t have breast milk secretion, therefore we give about two drops of prelacteal feeds. But as the ASHA workers told me not to give prelacteal feeds I started breastfeeding.

**Participant 9:** I fed breast milk only.

**Participant 7:** Only one drop of honey was fed to my baby because there was no breast milk immediately after delivery.

**Participant 6:** One drop of honey was given to my baby as I had no breast milk immediately after delivery.

**Participant 2:** My baby was crying continuously after birth therefore sugar water was fed to the baby.

1. **What do you and people around you think about colostrum?**

**All Participants (In chorus):** It should be fed to the baby.

**Participant 3:** Only mother’s breast milk should be fed to the baby. It is good for the baby. If any other milk is fed to the baby it doesn’t get digested by the baby.

**Participant 9:** Colostrum is good for babies therefore it should be given to the babies.

**Participant 6:** The thick milk (Colostrum) should be given to the baby. By that the baby’s health will improve.

**Participant 7:** If the baby is fed with colostrum its health improves and it protects the baby from diseases.

**Participant 1:** Colostrum should be given to the baby.

**All Participants (In chorus):** We all have fed our baby with colostrum.

1. **What do you and people around you think about exclusive breastfeeding?**

**All Participants (In chorus):** People around us also tell us to breastfeed exclusively for six months.

1. **What do you and people around you think of gripewater /gutti /any medicines during the first six months?**

**Participant 1:** I have fed gutti bottle and gripe water to my baby. If gripe water is fed the baby will not be irritant. The gutti which is made of almond and dry date is fed to my baby for one month, later gutti bottles available in the shops were brought and fed to my baby. The people in the community also feed these things to their babies.

**Participant 2:** I fed gutti prepared from almond and dry date mixed with breast milk and I also fed gripe water to keep the baby stomach clean. I have fed these to my baby for 6 months. The people in the community also give these things to their babies.

**Participant 3:** I also fed gutti prepared by paste of almond and dry date to my baby and I also fed gripe water to relive the pain abdomen of the baby. I fed these for 5 – 6 months. People in our community also give these things to the babies.

**Participant 4:** I fed gutti bottle and gripe water to my baby for 6 months. Gripe water was fed to baby to relive pain abdomen.

**Participant 5:** I fed almond, dry date and turmeric paste to my baby after three months for two days, but later I fed gripe water to the baby.

**Participant 6:** I fed a mixed paste of dry date and almond to my baby two times. I have not fed gripe water to my baby.

**Participant 7:** I fed gripe water, gutti and tim tim bottle to my baby.

**Participant 8:** I fed gripe water and gutti because the baby used to have pain abdomen after feeding top milk.

**Participant 9:** I fed almond, dry date mixed with breast milk for 3 months.

**Participant 10:** I have not fed gripe water I have fed only Gutti made of dry date and almond.

1. **Anything else you would like to add that you think is important?**

**Participant 6:** Earlier when I was not knowing about breastfeeding the baby. Even though my mother helped me to hold the baby to breastfeed, I couldn’t breastfeed for first 3 days as I had pain in my breast. I used to express the breast milk and feed the baby.

**Participant 10:** My baby was admitted in NICU and I had not breast fed my baby for 2 hours. Then the ASHA worker came and told me to breast feed and immediately I breastfed my baby.

**Participant 9:** ASHA workers used to come to us regularly for 3 months and they were telling us to breastfeed my baby because the breast milk contains all the nutrients. If breast milk is not fed, I will have swelling in my breast, therefore I breast fed my baby for 2 years.

**Participant 3:** My baby had low birth weight and it was given kangaroo mother care. I used to express my breast milk and it was being fed with the help of a paladai.

**Participant 2:** During my previous delivery I had wounds on both the nipples and it was very painful to breastfeed the baby. Therefore, my baby was fed with top milk (Goat milk) for 3 months. Later when wounds were healed I started breastfeeding my baby.

**Participant 1:** During my first delivery I had pain in my breast. The pain was there for two days and it subsided by itself.

**Participant 4:** For the first two days after delivery I had difficulty in breastfeeding and I fed sugar water to my baby.

**Participant 6:** Initially the baby was not breastfeeding properly, it used to feed a little and was going into sleep. Due to my incomplete breastfeeding I had breast engorgement. **Participant 7:** I had no problem.

**Participant 8:** I had retracted nipples on both sides and even though I had enough breast milk I could not feed the baby; therefore, I had breast swelling and the baby was fed with top milk.

**Participant 5:** At seven months of my pregnancy ASHA worker used to tell me to massage my breast. I followed her advice and had no problem.

**Breastfeeding perceived control**

1. **What makes breastfeeding easier?**

- **It is answered under previous question.**

1. **What makes breastfeeding harder?**

**Participant 3:** When the breast is engorged it becomes difficult to breastfeed.

**Participant 5:** I don’t have any idea.

**Participant 7:** If the baby goes into sleep for a long time, the mother will have tightness and pain in her breast due to excessive storage of breast milk.

1. **What does your community think of roles played by maternal depression on breastfeeding?**

**All Participants (In Chorus):** The maternal mental depression will have effect on her breastfeeding.

1. **What does your community think of roles played by maternal smoking on breastfeeding?**

**All Participants (In chorus) except participant 4:** No breastfeeding mother chews tobacco in our community.

**Participant 4:** In my community there is only one mother who chews tobacco.

1. **What does your community think of roles played by maternal drinking on breastfeeding?**

**Participant 10:** Yes, I have heard but I have not seen any mother in the community taking alcohol.

**Participant 9:** In my family they were telling that if the mother takes alcohol she will get relief form the pain of delivery.

**Moderator probed the question to Participant 7.**

**Participant 7:** Ah…. (The participant got little shocked) this is first time I am hearing this thing.

**Rest of the participants (In chorus):** No, we have not heard about it.

1. **What does your community think of roles played by maternal drug use on breastfeeding?**

**All Participants (In chorus):** We don’t know about it.

1. **What does your community think of roles played by maternal anaemia on breastfeeding?**

**Participant 1:** There will not be any effect.

**Participant 2:** Yes, there will be effect.

**Participant 3:** Yes, there will be effect.

**Participant 4:** Yes, there will be difficulty.

**Participant 5:** Yes, there will be difficulty.

**Participant 6:** Yes, there will be difficulty.

**Participant 7:** Yes, there will be difficulty.

**Participant 8:** Yes, there will be difficulty.

**Participant 9:** Yes, there will be difficulty.

**Participant 10:** Yes, there will be difficulty.

**Moderator comment: Nine participant said that the maternal anaemia will have adverse effect on breastfeeding.**

**Moderator:** What difficulty the mother will have in breastfeeding?

**Participant 2:** The mother will not have enough strength to breastfeed the baby.

**Participant 6:** The baby will not have any difficulty.

**Participant 3:** The baby will not have any problem because there will be enough breast milk in the mother if she takes adequate food.

**Participant 5:** If the mother is anaemic, she may have a problem but the baby will not have any problem.

**Participant 7:** If the mother is anaemic the baby will have less milk.

**Participant 8:** The baby will not have any difficulty. The mother may suffer due to anaemia.

**Participant 9:** The baby will not have any problem.

**Participant 10:** The mother will have symptoms like pain in the arms and legs.

1. **What does your community think of roles played by maternal overweight or obesity on breastfeeding?**

**Participant 10:** No, the obesity of the mother will not have any effect on her breastfeeding.

**Participant 9:** I have heard that the obese mother has large breasts which fall on the babies face and may suffocate the baby.

**Participant 8:** No, it doesn’t affect breastfeeding.

**Participant 7:** No, it doesn’t affect breastfeeding.

**Participant 4:** No, it doesn’t affect breastfeeding

**Participant 3:** No, it will not effect on breastfeeding

**Participant 2:** The breasts of obese woman press on the nose of a baby while breastfeeding. It may cause difficulty in baby’s breathing. The mother has to breastfeed her baby lying down position as it is difficult for her to breastfeed in sitting position.

1. **What does your community think of roles played by intimate partner violence on breastfeeding?**

**Participant 9:** If the mother in law, father in law or husband quarrel with the breastfeeding mother she will have mental depression but it will not affect her breastfeeding.

**All Participants (In chorus):** If the woman undergoes violence by her husband, it will affect her breastfeeding.

1. ***Acceptability of breastfeeding peer counselors (10 minutes)***
2. **Where do you get information about breast feeding?**

**It is answered under question 2 and 4 (Section B).**

1. **If you have any breast feeding problem whom do you approach?**

**Participant 1:** ASHA workers help us.

**Participant 2:** Staff nurses and ANMs help us.

**Participant 3:** My mother, mother in law and aunty help me.

**Participant 10:** Mother in law.

**Participant 6:** In the house my mother, father and sister help me.

1. **Are you aware of any programmes by the government to promote breast feeding practices in the country?**

**All Participants (In chorus):** We don’t know any programme. (Nodded their head as indicating they don’t know).

1. **Is there any support of the women from the village/community?**

**Participant 8:** Yes, the people in the community comes and help us.

**Participant 10:** In our village the elder women in the community help us in breastfeeding.

**Participant 9:** ASHA worker comes and helps us.

**Participant 7:** ASHA worker and ANMs help us.

**Participant 6:** ASHA worker and elder women in the village come and help us.

**Participant 5:** Grandmothers in our village help us.

**Participant 4:** Grandmothers help us.

**Participant 3:** At our place also grandmothers help us

**Participant 2:** Grandmothers help us.

**Participant 1:** Aunties help us.

1. **Is she trained in giving breast feeding support and help?**

**All Participants (In Chorus):** No, they are not trained.

**Participant 2:** The grandmothers even though they are not trained have the experience of doing this work for a long time.

**Breastfeeding peer attitude**

1. **Do you think that a person (Peer Counsellor) from your community/village, if trained will be helpful to solve the breast feeding problems?**

**All Participants (In chorus):** Yes, it will be helpful.

1. **Do you accept receiving Breast feeding counselling and advice from such a person?**

**All Participants (In chorus):** Yes, All the people in the community accept such a person.

1. **What should be the qualifications of Peer Counsellor?**

**Participant 3:** She should be having all the knowledge about breastfeeding.

**Participant 1:** She should have caring attitude towards the mothers.

**Participant 2:** She should have clean mind (Good nature)

**Participant 7:** She should be friendly, understand our problems and if any problem to us she should be able to solve it.

**Participant 8:** She should have good heart. She should listen to what we say to her.

**Breastfeeding peer perceived control**

1. **What would be the benefit to have Peer Counsellor?**

**Participant 6:** If we don’t know any information she will tell us.

**Participant 9:** If we don’t know about anything and if she tells us properly we will understand.

1. **What would be the barriers/problems to have Peer Counsellor?**

- **This question was not asked.**

**Breastfeeding peer social norm**

1. **Who approves the presence of Peer Counsellor?**

**All Participants (In Chorus):** All people in our community accept and approves the presence of peer counsellor.

1. **Who disapproves the presence of Peer Counsellor?**

**Participant 8:** One or two people may be there who may not approve her but rest of the community approves the presence of peer counsellor.

**Participant 2:** One or two people may be there who do not accept her presence.

1. ***Program to train breastfeeding peer counselors (10 minutes)***
2. **How does the health worker help you in breast feeding activities?**

**Participant 9:** Immediately after delivery ASHA workers come and tell us about breastfeeding.

**Participant 10:** After delivery ASHA workers tell us about breastfeeding.

1. **Does the health worker visit and advise you about Breast feeding before delivery?**

**AND**

1. **What does the health worker talk about breastfeeding antenatally?**

**Participant 6:** Before delivery when the health workers used to come to us for BP measurement they used to tell us about breastfeeding. They used to tell me to breastfeed my baby within 15 minutes after delivery.

**Participant 7:** During my first pregnancy, ASHA worker visited me and told me to breastfeed my baby immediately after delivery.

**Participant 8:** To me also the same thing was told.

**Participant 4:** They had advised me to breastfeed the baby immediately after birth.

**Participant 9:** To me also they had told the same.

1. **What happens at birth? Is she present at birth and help you with early starting of Breast Feeding?**

**Participant 7:** At the time of my delivery ASHA worker had come to hospital and she was with me. She also told me to breastfeed soon after delivery.

**Moderator:** Was health worker present at the time of delivery?

**All Participants (In chorus):** Yes, ASHA worker was present with me at the time of delivery.

1. **How frequently does she visit you after birth of the baby?**

**Participant 9:** During the first 3 months the health workers visit us in our house once in a week.

**Participant 10:** Yes, they had come.

**Participant 8:** After my delivery when I was still in hospital ASHA worker had visited me. After discharge I went to my mother’s place.

**Participant 7:** Only once she had come to my house.

**Participant 6:** 3 to 4 times she had come to my house.

**Participant 5:** Once in a week.

**Participant 4:** ASHA worker is staying near my house and she used to visit me frequently.

1. **What is the purpose of visit?**

- **This question was not asked.**

1. **How frequently does she assess your breast feeding practices?**

**Participant 9:** The health worker had observed me breastfeed the baby in my house.

**Participant 7:** After delivery the ASHA worker visited me and advised to breastfeed my baby.

1. **How many mothers does the health worker visit per day/per month?**

- **This question was not asked.**

1. **What are the common breast feeding problems you observe in your community?**

**Participant 9:** One of my relative did not breastfeed her baby from one breast as she was unable to hold the baby to breastfeed from that side. Her parents and ASHA worker told her to breastfeed from both side but she didn’t listen to them. She had breast engorgement on that side and it had to be operated. Now also she is breastfeeding from one side only.

1. **Do you think that peer counsellor who is from the same community would help and support mothers?**

**Already answered all participants said that it will be helpful to have peer counsellor.**

1. **What do you think would be the barriers for Peer Counsellors in the community?**

**All participants (In chorus):** There will not be any barriers to the peer counsellor, all people in the community support such peer counsellor and allow her to work.

1. **Will there be future opportunities for these Peer Counsellors to continue in the community?**

**Participant 9:** Yes, there will be opportunity.

**Participant 4:** It will be useful in future to get any information from her.

1. **What should be the criteria for acceptance of such counsellors by you and the community?**

- **It is already answered under Section B. Question 8.**

1. **How do you identify and recruit such counsellors?**

**Participant 1:** Such person is available in my community.

**Participant 2:** Yes, available in my village.

**Participant 3:** Yes, they are available.

**Participant 4:** Yes, they are available.

**Participant 5:** Yes, available.

**Participant 6:** Yes, available.

**Participant 7:** Yes, available.

**Participant 8:** Yes, available in my village.

**Participant 9:** Yes, they are available in village.

**Participant 10:** Yes, available.

**F. Wrap up**

1. ***Ask participants***

**“How did you feel about participating in this session? What was easy? What was hard?”**

1. ***Summarize and thank participants by saying:***

**“We appreciate all of your great ideas. You have been a big help, and we want to thank you very much for all the information you have shared with us today. We know that your ideas will help to make this a successful program and technology. Thank you once again for your participation – we really appreciate you!”**

**Thank you**

**Focus group interview guide**

**Materials Needed:**

Food and Beverages

Newsprint & Markers

Masking tape

Nametags

Audiotape recorders and tape

Participant incentives

Questionnaires

Pencils

Attendance sheet

**Introductions and Focus Group Process (10 minutes)**

1. *Team members will have nametags on prior to participants entering the focus group venue*
2. *Give participants nametags as they arrive.*
3. *Instruct participants to select celebrity name*
4. *Have participants sign-in on attendance sheet.*
5. *Explain the purpose of the focus group session by saying:*

**“Welcome to today’s focus group. We are planning to develop mobile technology to support breastfeeding peer counselors and breastfeeding mothers. We asked you to come to today’s session because we would like to hear from you about your opinions and ideas on the technology content. You are the experts, and we can learn from you. We need your honest opinion – good and bad – about what we are trying to do. We would like you to share what you think – and what you think other members in your community might think about our project. Everything you are thinking is important to us. There are no right or wrong answers. We value your opinion. We would be very happy if you would help us to make the best technology possible.**

**Please remember to use your celebrity name and refer to others with their celebrity names. Also, try not to use any friends’ names or specific locations. But if you do, we will delete them from the audio recording.**

*As a reminder we are going to turn on the tape recorder now.*

*Is everyone ok with that? TURN TAPE ON: and announce*

“This is focus group # 05 on 04 / 04 / 2018 for the “BEST4Baby”

*1. Ask the participants to introduce themselves by saying their celebrity name and what is your favorite color.*

*2. All members of investigative team introduce themselves.*

**“Now, we would like to develop some group rules so we can learn as much as possible from each other.”**

**Group Rules (10 minutes)**

1. *Develop group rules to protect participants’ confidentiality. Offer examples of group rules if participants are stuck:*

Be respectful

Be honest

Maintain confidentiality

One person speaks at a time

Listen to others

No put downs or insults

Turn off all cell phones

**“Even though we will be talking about breastfeeding and technology development today, we do not require you to talk about your personal experiences with breastfeeding if you do not feel comfortable. You can instead share what you think or others’ experiences. However, please do not use any specific names or identifiable information of others for protecting their privacy. Is that clear to everyone?”**

**BREAST FEEDING EDUCATION SUPPORT TOOL for BABIES**

**Focus Group Discussion guide for Mothers who**

**have successfully breastfed at least one child in the past three years**

**Discussion (40 Minutes)**

**“We are working to develop a mobile technology to support breastfeeding peer counselors and breastfeeding mothers in India. We want to know what you think and what you know about breastfeeding practices. Your inputs will certainly help develop feasible and effective technology to support breastfeeding. We have questions we prepared but we will let our group guide our own discussion. Let’s start with some initial questions about breastfeeding experiences.”**

1. ***Breastfeeding practice and support mechanism (20 minutes)***
2. **Tell us about your breast feeding experience.**

**Participant 1:** I have breastfed immediately after delivery. The baby started vomiting after breastfeeding. The baby was admitted in NICU for seven days as it had jaundice. I was told to express the breast milk and feed the baby for four days. I started breastfeeding on fourth day. The baby also had stenosis of anus for which it was operated.

**Participant 2:** I delivered by caesarean. Within half an hour the baby was given to me for breastfeeding. Even though I had caesarean I sat up and breastfed my baby. Now the baby is fine. For six months exclusively I breastfed the baby. There after I started rice porridge along with breastfeeding.

**Participant 3:** We should breastfeed the baby within one hour of birth.

**Moderator:** When did you breastfed your baby?

**Participant 3:** I breastfed within twenty minutes. I had no problem in breastfeeding.

**Participant 4:** I had also delivered by caesarean section. I was unconscious for two hour after caesarean; therefore I started breastfeeding two hours after delivery. Then I continued to breastfeed for six months. After six months I gave rice porridge, rice and cattle milk to the baby along with breast milk.

**Participant 5:** I started breastfeeding twenty minutes after delivery. I am still breastfeeding and there is no problem in breastfeeding.

**Participant 6:** My baby was given to me five hours after delivery. Therefore I could breastfeed the baby after five hours.

**Moderator:** Where was the baby till five hours? And why it was not given to you?

**Participant 6:** The baby was with my husband. It was meconium stained; therefore it was cleaned, treated and then given to me.

**Participant 7:** After ten minutes of my delivery the baby was cleaned and given to me and I started breastfeeding. By breastfeeding postpartum bleeding will be controlled. By breastfeeding the emotional bondage between the mother and the child increases.

**Participant 8:** I also had caesarean. Half an hour after caesarean, the baby was given to me for breastfeeding. I started breastfeeding but I could not get breast milk initially. After ten to fifteen minutes of trying, I got my breast milk secretion and I successfully started breastfeeding the baby. Now the child is three months old and it is doing fine.

**Participant 9:** At the time of delivery I had high BP of around 320mmHg (As per her statement). Therefore, I had to undergo caesarean delivery. The baby’s weight was very less and it was admitted in NICU for 3 days. I was unconscious for 27 days. After 27 days the baby was given to me for breastfeeding. I breastfed my baby for three months. But after three months my breast milk dried up. Then I went to the doctor to consult and I was told that my breast milk was supressed by medicine to prevent my breast engorgement. So I could breastfeed only for three months and after that I am giving top milk to my baby.

**Breastfeeding attitude**

1. **Do you think breast feeding is important for the baby?**

**All Participants in chorus agreed that breastfeeding is important for the baby.**

**(All participants raised their hands and said “Yes…yes… it is important”. Everyone nodded their head in acceptance).**

1. **Why? What are the benefits?**

**Participant 1:** Whatever good things we eat goes to baby through our breast milk and babies grow well.

**Participant 2:** Baby will be healthy and it will not have any diseases. Even if there is fever in baby we should not stop breastfeeding; because the breast milk itself is a medicine for baby. Whatever calcium we eat in our food it goes to baby and it becomes healthy and it will not have any illnesses. Therefore we must breastfeed the baby.

**Participant 3:** There is vitamin A in the breast milk. If we give top feed the child will have diarrhoea. There will be no problem to the baby if it is breastfed.

**Participant 4:** By breastfeeding, the baby will have good growth, weight gain and the baby will have strength to walk and move.

**Participant 5:** If the baby is breastfed it will have good growth and it will not have growth retardation. If we don’t breastfeed the baby early, its brain will not have good growth and it will not have any heart problem. The baby will have good blood circulation.

**Participant 6:** If the baby is top fed, it will not have good growth. Therefore, we should breastfeed the baby.

**Participant 7:** By breastfeeding, there will be good emotional bonding between the mother and child. It protects the health of the baby from many diseases.

**Participant 8:** The baby will have good growth and it will not have any problems that may lead to admission to NICU.

**Participant 9:** If we breastfeed, baby will become strong. And it will walk and move at an early age.

1. **If you think breastfeeding is not good, what would be the reasons?**

**Majority participants said breastfeeding is good.**

**Participant 3:** If the mother has blood cancer or any other illness, it is not good to breastfeed the baby.

**Participant 4:** If there is any condition like as told by participant 3 one should not breastfeed.

**Participant 5:** When the mother has got ulcer and swelling of the breast, it is not good to breastfeed the baby. If the mother breastfeeds in such condition the baby becomes weak.

**Participant 6:** If there is swelling in the breast (She meant breast abscess), it is not good to breastfeed. Because there is pain in the breast and it will not produce clean milk.

**Participant 7:** If the mother is not well, she should not breastfeed. If she breastfeeds when she is ill, the baby also may become ill.

**Participant 8:** It is always good to breastfeed.

**Participant 9:** If the mother is having fever, she should not breastfeed. The baby will also have fever, if she breastfeeds.

1. **How long does the mother need to breast feed exclusively and the total duration?**

**Moderator:** How long does the mother need to breastfeed exclusively?

**Participant 1:** The mother should breastfeed exclusively for seven months.

**Participant 2:** For six months.

**Participant 3:** For six months.

**Participant 4:** For six months.

**Participant 5:** For six months.

**Participant 6:** For six months.

**Participant 7:** For six months.

**Participant 8:** For six months.

**Participant 9:** For six months.

**(They also said that after six months the baby can be given thin rice porridge, soft fruit juice, soft food and top milk)**

**Moderator:** For how long the breastfeeding should be done totally?

**Participant 9:** One and half years.

**Participant 6:** One and half years.

**Participant 8:** Two to three years.

**Participant 7:** Two to three years.

**Participant 4:** Two to three years.

**Participant 5:** Three years.

**Participant 3:** Two to two and half years.

**Participant 1:** Two years.

**Participant 2:** Two years.

**Breastfeeding social norm**

1. **Do all of the mothers in the community breastfeed?**

**All the participants said that all the mothers in their community breastfeed their baby. Participant 2: Few working women in our community breastfeed their babies when they are at home. When they are at work away from home, the babies are fed with top milk. (Participant 7 and 8 also have same opinion.)**

**Participant 5:** “One of my close relative is not at all breastfeeding her baby. Her four earlier babies died at an early age. Her family members thought that her breast milk was poisonous and the babies died due to her breast milk only”.

**Moderator:** What happened to present (fifth) baby?

**Participant 5:** This baby is not breastfed; it is fed with top milk. And it is alive.

1. **Who approves breastfeeding around you?**

**Moderator:** Immediately after delivery who approves you to breastfeed the baby?

**Participant 9:** My aunty told me to breastfeed.

**Participant 8:** We ourselves breastfeed the baby and the doctors in the hospital also tell us to breastfeed the baby.

**Participant 7:** Doctors and my family members tell us to breastfeed.

**Participant 6:** Doctors, ASHA workers and my mother in the family tell us to breastfeed. **Participant 5:** Doctors and family members tell us to breastfeed the baby. We on our own breast feed the baby.

**Participant 4:** Doctors, my mother and my mother in law tell us to breastfeed the baby.

**Participant 3:** Doctors, ASHA workers, Elders in the community, grandmother and other family members tell us to breastfeed the baby.

**Participant 2:** Doctors and my mother tell us to breastfeed the baby.

**Participant 1:** Doctors and my mother tell us to breastfeed the baby.

1. **Who disapproves breastfeeding around you?**

**All participants (In chorus) : “Nobody in the community disapproves breastfeeding.”**

1. **What do you and people around you think about prelacteal feeds?**

**Participant 7:** Some people feed honey and sugar water before breastfeeding.

**Participant 6:** Sugar water is fed before breastfeeding.

**Participant 9:** If sugar water is fed the baby’s lungs become congested. Therefore it should not be given.

**Participant 8:**  The child should be fed with breast milk only. Honey should not be fed to the baby.

**Participant 5:** Doctors gives polio drops before breastfeeding.

**Participant 3:** Sugar water, cow milk and honey are given; but all these should not be given as prelacteal feeds.

**Participant 2:** Only sugar water and honey is fed to the baby before breast milk.

**Participant 4:** If the mother doesn’t have breast milk, doctors prescribed milk powder.

**Participant 1:** They feed only sugar water.

**Participant 8:** People say to feed honey and sugar water only before breastfeeding.

**Participant 7:** If the mother undergoes caesarean delivery, she is not able to sit and breastfeed. At that time doctors prescribe milk powder to the baby. And cow milk is also fed to baby.

**Statement by moderator: (All Participants say that the people in the community especially the elders tell them to give prelacteal feeds like honey and sugar water. But the mothers of present generation including the participants don’t like to give prelacteal feeds to their babies.)**

1. **What do you and people around you think about colostrum?**

**Participant 1:** It is good to feed colostrum to baby. People in the community also say that it is good to breastfeed the colostrum.

**Participant 2:** People in the community ask us to feed the colostrum. It is good for health and it prevents any disease coming to the baby.

**Participant 3:** We should feed the first milk (Colostrum). Because it contains vitamin A and provides immune power. And people also tell us to feed it.

**Participant 4:** We should breastfeed the thick milk (Colostrum). Even if there is any problem to us, the mother or the mother in law helps us to breastfeed colostrum to the baby. It is good for baby’s health and people also tell us to feed it to the baby.

**Participant 5:** Compulsorily we should feed the colostrum. People also tell us to feed it.

**Participant 6:** We should feed it compulsorily and nobody tells us not to feed it to the baby.

**Participant 7:** Soon after birth it should be fed to the baby and it prevents any disease to the child.

**Participant 8:** It should be fed. It contains immune power and with that the baby will grow properly.

**Participant 9:** It should be fed. It increases the quality of the blood of the baby.

**Participant 3:** It has immune power and it prevents the disease coming to the baby.

**Participant 2:** It should be fed. It helps in the growth of the brain and increases the memory power and intelligence. It helps in the growth of ears, eyes and other organs properly.

**Participant 1:** It helps in the growth of the child and the growth of eyes, ears and other organs properly.

**Participant 4:** It is good for the baby.

1. **What do you and people around you think about exclusive breastfeeding?**

**Participant 8:** Some women, who go outside for work feed top food along with breast milk. Elders in the community tell us to feed top food along with breast milk. Now a days after the arrival of powder milk in the market, the people in our neighbourhood feed it to the babies before six months.

**Participant 7:** People tell us to feed the baby with almond, dry dates along with the breast milk in the first six months.

**Participant 6:** Immediately after five months people tell us, “Don’t feed too much breast milk only; along with that feed crushed banana and kichadi (soften rice) to make the baby strong to stand up” . But my opinion is to feed breast milk only till six months.

**Participant 5:** If the child is obese, people say your milk only is not sufficient to the child; give some top food so that the baby will not become hungry and irritant.

**Participant 4:** If we continue the breastfeed the baby will not stop breastfeeding. Therefore people tell us to give some top feeds like cow milk and buffalo milk along with breast milk, thinking that only breast milk is not sufficient for the child before six months. But I have not given any top feed.

**Participant 3:** People tell us to give some liquid top feeds like cow milk, sheep milk and semolina porridge.

**Participant 2:** In my village people tell us to breastfeed up to six months and not to give any top feed.

**Participant 1:** I have breastfed till six months and along with that I have fed dry dates and almond paste. People also tell us to give these top feeds.

1. **What do you and people around you think of gripewater /gutti /any medicines during the first six months?**

**Participant 1:** I have fed gutti by mixing almond and dry dates within six months. People say that it is good for the baby and I also feel that it is good for the growth of the baby.

**Participant 2:** I have fed the gutti for the first few days only. My mother in law advised me to do so. It is prepared by various herbal plant nuts (Arali Kaai, Bajji, Dry date, Kyara seed, Ajavain) they say that it prevents cough and fever. I fed it because elders in the family told me to do so.

**Participant 3:** People tell us to feed the gripe water, gutti and some medicines so that the baby sleeps well. But I have not fed it.

**Participant 4:** If the baby is irritating we feed gripe water so that it sleeps well. And when we take it to the hospital the doctor prescribed some medicines. If it is good for the baby’s health we continue it. If we give medicine the baby sleeps well and improves.

**Participant 5:** If the baby is weak, people say to us “Your children are very weak; feed them Cerelac (Artificial baby feed) or gutti so that they will become strong”. My feeling is, even though the children become obese, it is only hollow fatness without any strength inside and they will not be healthy. (She laughs)

**Participant 6:** People told me “Your baby is very weak, feed it gutti or something which is available in medical shop”. My family members were ready to bring it from medical shop; but I told them that I would not feed it to my baby.

**Participant 7:** I fed almond and dry date because, by doing so the children will grow well. And if we give Bala kadu (A type of Ayurveda medicine) it prevents cough and cold.

**Participant 8:** People tell us to feed, but I don’t feed. Only breast milk is sufficient for the baby and there will be no problem.

**Participant 9:** I fed top feed to my baby for three months because my baby was very small weighing one kilogram only. As per doctor’s advice I fed the baby with gripe water, dry date and almond. Since then the baby started growing well. Blood transfusion was done to my baby and to me. I had high blood pressure during delivery. For the first three months I gave breast milk to my baby with the help of my mother and mother in law. After three months I had fever and my breast milk dried up and I started feeding cow milk.

1. **Anything else you would like to add that you think is important?**

**Participant 7:** All doctors tell us to breastfeed our babies. We need to breastfeed the baby frequently at least 8 to 9 times per day so that the baby’s health is maintained. The mother’s breast milk is the main food for the baby.

**Breastfeeding perceived control**

1. **What makes breastfeeding easier?**

**Participant 1:** It is good to breastfeed.

**Participant 7:** During antenatal period and near the date of delivery, the mother should keep her breast clean. She can do this by applying oil and massaging while taking bath so that she can easily breastfeed her baby immediately after birth and the baby will not have any diseases. As per doctor’s advice the mother should take nutritious food.

**Participant 8:** During pregnancy, whenever time is available she should apply oil and massage her breast. By doing so, the baby will get breast milk immediately after birth.

**Participant 9:** During pregnancy the mother should eat nutritious food and fruits, so that she will have good breast milk for her baby.

**Participant 6:** The mother should maintain cleanliness even after delivery so that the baby breastfeeds easily. If the mother doesn’t maintain cleanliness she may have swelling of her breast.

**Participant 5:** I don’t know much about this.

**Participant 3:** We should breastfeed the baby frequently. If we breastfeed the baby only two times a day the breast milk will dry up. We must breastfeed every half an hour. As we breastfeed the baby the breast milk secretion goes on increasing and both the mother and the baby will be healthy.

**Participant 2:** If the mother does not breastfeed properly she will have breast engorgement. The mother will have pain in the breast and it will be difficult for the baby to breastfeed. The mother should apply oil to her breast and massage and after that it should be cleaned by a cloth soaked in warm water. By doing so, she will have easy breast milk secretion.

**Participant 7:** The elders in the family tell the mother to eat green vegetables to have good breast milk.

**Participant 1:** While taking bath, the mother should massage and clean her breast with oil during antenatal period.

1. **What makes breastfeeding harder?**

**Participant 1:** When my baby was admitted in NICU it became difficult for me to breastfeed. I used to express my breast milk and was giving it to feed with spoon. When I was not breastfeeding for the first four days my breasts were painful.

**Participant 2:** If we do not breastfeed the breast becomes painful. Therefore we should breastfeed. After caesarean delivery it is difficult to sit up and to breastfeed. Some mothers do not have milk secretion and they top feed their babies with cow milk. Some mothers don’t know how to breastfeed their babies even though there is milk in their breasts. They also don’t know how to hold the baby to breastfeed.

**Participant 3:** If we don’t breastfeed frequently the milk secretion becomes blocked and the mother may have engorgement of the breast. If the mother doesn’t breastfeed she will have swelling in the breast.

**Participant 4:** If it is her first baby, the mother may not be knowing how to breastfeed. Even if other mothers in the hospital, her mother or mother in law try to tell her how to breastfeed she may be unable to understand.

**Participant 5:** If we don’t breastfeed the breasts get engorged. When I had this problem my family members told me to do hot fomentation of the breast but I had breast abscess and had pus and blood coming from it.

**Participant 6:** For one day my baby did not breastfeed and I got swelling of the breast which turned out to become an abscess and I got operated for it. There was one mother in my area whose baby was not breastfeeding; she had both of her breasts engorged. She consulted a doctor, but even now she has swelling in one of her breasts. She tried to breastfeed her baby but the baby was not feeding properly.

**Participant 7:** If the mother doesn’t breastfeed her baby, she will have engorgement of her breast. If the mother is bleeding heavily after delivery, she cannot sit and breastfeed her baby early. If there is illness of the mother or the baby it will be difficult for her to breastfeed.

**Participant 8:** There was one mother next to me in the hospital. She had retracted nipple on both sides and she could not breastfeed her baby. Some women wear innerwear which becomes a problem for breastfeeding the baby.

**Participant 2:** Even when there is excessive milk secretion, it will be a problem for breastfeeding, as the nipple is large and the baby cannot hold it in its mouth and suck. In that case, if the mother expresses some milk and throws it away then it will be easy for her to breastfeed.

**Participant 9:** If the baby is too small then also it is difficult to hold the nipple and breastfeed. When the mother has undergone caesarean and there are sutures on her abdomen, it is difficult for her to breastfeed.

**Participant 7:** If the mother had an operation on her breasts she will have difficulty in breastfeeding.

1. **What does your community think of roles played by maternal depression on breastfeeding?**

**(All Participants (In chorus) said “Yes, the mental depression has an effect on breastfeeding”.)**

**Participant 1:** Due to mental depression of the mother her breast milk secretion becomes less. (Participant 2 & 3 also have the same opinion)

**Participant 4:** If the mother is mentally depressed, she will be in her own world, detached from the surroundings and she may not give proper attention towards her baby. This may affect her breastfeeding.

**Participant 5:** If the mother has only male children or female children continuously, her family members become unhappy with her. Due to the behaviour of family members, the mother becomes mentally depressed and she may not take proper nutritious food which will have an effect on her breast milk secretion. Even if she wants to breastfeed her baby, there will not be sufficient breast milk in her.

**Participant 6:** If the mother is mentally depressed, she will not have breast milk secretion. The mother and her baby will not be healthy. She may not have proper attention towards her baby. She will be in her own world. In our neighbourhood there was one mother who was mentally depressed because she delivered a female child and she had no attention towards her baby. The baby was one month old and was crying a lot. But the mother was not breastfeeding it. The mother died two months after delivery and the baby is alive but very weak.

**Participant 7:** Due to mental depression the mother will have insufficient breast milk and it will have effect on her baby’s health.

**Participant 8:** If the mother is mentally depressed, she will not take proper food and cannot give proper attention towards the baby. It will have effect on her breastfeeding and baby becomes weak.

**Participant 9:** If the mother is mentally depressed because of disputes with her mother in law or father in law, she may not give proper attention towards her baby. She will have less breast milk secretion and she cannot breastfeed her baby adequately.

**Moderator:** What the community think about mother’s mental depression will have effect on breastfeeding.

**Participant 9:** The community people advice the mother as “Do not take too much into your mind if you worry, the breast milk secretion will come down and your baby will not grow properly.”

**Participant 8:** People say “Do not worry much about your problem. Give attention towards your baby because it is too small. You can think about your problem later on.”

**Participant 7:** People say “Do not get depressed, if you get depressed you will have less breast milk secretion therefore do not stop breastfeeding”.

**Participant 6:** People advise us, “Do not take too much tension in your mind, it will reduce your breast milk secretion and it will have effect on your baby.

**Participant 5:** They console the mother and ask her to take proper care of the baby.

**Participant 4:** People advise us saying, “Do not take too much of tension; it will bring down your breast milk secretion. You take proper food and buffalo milk and give attention towards the baby”.

**Participant 3:** People say, “If you have mental tension you will not have breast milk. So don’t take mental tension”.

**Participant 2:** People say “Do not take mental tension, it will not last long. If the new born baby does not grow now, how it will grow in future? So, do not have too much mental tension and take care of the baby and breastfeed it”.

**Participant 1:** People say to us, “Take proper food. If you don’t have proper food you will not get sufficient breast milk.”

**Common opinion of all Participants about community thinking about maternal depression and its effect on breastfeeding.**

**Moderator Comment: Community people advice the mothers saying, “Don’t take too much mental tension; eat proper food, otherwise it will reduce your breast milk secretion and the baby will not have proper growth.”**

1. **What does your community think of roles played by maternal tobacco usage on breastfeeding?**

**Majority participants (In chorus) said some mothers chew tobacco after delivery.**

**Participant 1:** The mother will have chest pain and the baby also may have it. One of my close relative chews tobacco and she has this habit since before marriage.

**Participant 2:** The mother can breastfeed her baby but the babies will have repeated illness of cough and chest infection. The babies will have the same bad effects which the mother has due to tobacco chewing. Therefore breastfeeding mother should not chew tobacco.

**Participant 3:** The mother gets cancer due to tobacco chewing and the baby may also have cancer. Therefore one should not chew tobacco.

**Moderator:** Do the people in your community approve the tobacco chewing of breastfeeding mothers?

**All Participants (In Chorus) said, the community people tell the mothers not to chew tobacco.**

**Participant 2:** People say to mothers, “Eat something nutritious rather than chewing tobacco”.

**Participant 4:** If the mother chews tobacco the baby may get breathlessness and other diseases. Therefore the local people tell us “Now, you are pregnant why you are chewing tobacco? What benefit will you get from it?” Some mothers discontinue after listening to the people; but some will continue to chew tobacco”.

**Participant 5:** The mother may have cancer due to tobacco chewing. The baby may also have cancer through the breast milk. The mother’s breast milk secretion may be cut off because the tobacco contains many harmful chemicals.

**Participant 6:** Some mothers chew tobacco even though they are advised not to do so. Due to tobacco chewing its harmful chemicals go into the chest of the mother and through the breast milk to the baby also.

**Participant 7:** The children may get cancer which the mother had through the breast milk either during childhood or when they are grown up. And the child may have other diseases also. Therefore the breastfeeding mother should not chew tobacco. The community people also advice not to chew tobacco.

**Participant 8:** If the mother chews tobacco she may get cancer and other diseases. When she has some diseases doctor’s and the community advise her not to breastfeed her baby. And that will lead to adverse effect on the baby. By chewing tobacco the mother will have loss of appetite and she may not eat adequate food which will have effect on her breast milk secretion. She will have less breast milk secretion and she will be unable to breastfeed her baby which will have effect on the growth of the baby. The bad chemicals in the tobacco pass through breast milk and will affect the health of baby.

**Participant 9:** One mother who was my neighbour chewed tobacco even though her husband told her not to chew it at the seventh month of her pregnancy. Even though the people scared her saying, “The tobacco which you are chewing is going into the baby’s mouth; don’t chew it”. But she continued chewing tobacco and she told them “What you are saying is nonsense; I have been chewing it since long time; let my baby and me live or die, I don’t care and I will continue to chew tobacco”. Even though her husband beat her she did not stop chewing tobacco. At the time of her delivery also she had problem and the baby was very small, anaemic and of low birth weight. So it was admitted in NICU. The mother had cancer. Later on she stopped tobacco chewing and she was able to breastfeed her child.

1. **What does your community think of roles played by maternal drinking on breastfeeding?**

**Participant 5:** Yes. I have seen some mothers taking alcohol. Whether the mother delivers normally or by caesarean they take alcohol to get relief from the pain of delivery (She laughs while telling this).

**Moderator:** Whether she takes it herself or somebody gives it to her?

**Participant 5:** The elders in the family like the grandmothers gave her cashew alcohol to drink (She laughs while telling this) when she told them that she was having pain after delivery.

**Moderator:** How much alcohol they give her to drink?

**Participant 5:** One capful of the bottle (10ml). (She laughs while telling it)

**Moderator:** Does it have any effect on breastfeeding?

**Participant 5:** Yes, it does have effect on breastfeeding. After taking alcohol the mother becomes intoxicated and she will have deep and long sleep and even though the child is crying she cannot give attention towards breastfeeding her baby. She cannot wakeup for at least 24 hours from the sleep and feed her baby.

**Participant 6:** I have seen giving one glass of alcohol to the breastfeeding mother when she was having cough and they give one capful alcohol to the baby also when it is crying too much. Then both the mother and the baby go into sound sleep. Even though she wakes up she cannot get up and take her baby to breastfeed and as the baby is in deep sleep it cannot cry for breastfeeding. (During the entire conversation all the participants keep on laughing). And when one mother underwent caesarean she was given one glass of alcohol to relieve the pain of the operation. At that time the mother went into deep sleep and she could not breastfeed her baby even though the baby was crying.

**Participant 7:** I have not seen any mother taking alcohol but I have heard about it. It affects her breastfeeding.

**Participant 8:** I have not seen or heard about the breastfeeding mothers taking alcohol. (Participant 9, 1, 2 and 3 had the same opinion)

**Participant 2:** Some grandmothers say to give half spoon of alcohol when the baby is having cough and breathing difficulty.

**Participant 4:** If the mother takes alcohol during breastfeeding it may have effect on the baby in future. Therefore the mother should be careful about it.

1. **What does your community think of roles played by maternal drug use on breastfeeding?**

**All Participants (in chorus):** No, no… we have not seen and heard about breastfeeding mothers taking drugs.

**Participant 5:** Taking drugs may lead to kidney failure.

**Participant 7:** It does have effect on the health of the baby and baby may have any disease.

1. **What does your community think of roles played by maternal anaemia on breastfeeding?**

**Participant 1:** If the mother is anaemic the baby may not have sufficient blood. But it does not affect breastfeeding.

**Participant 2:** The mother’s anaemia doesn’t have effect on her breastfeeding.

**Participant 3:** If the mother is anaemic she will have less breast milk.

**Participant 4:** If the mother is anaemic she will have insufficient breast milk.

**Participant 5:** If the mother is anaemic she will not have breast milk.

**Participant 6:** If the mother is anaemic she will not have breast milk.

**Participant 7:** The mother can breastfeed her baby. But if she is anaemic, it will have an effect on the health of the baby.

**Participant 8:** I had caesarean and I was anaemic; but it did not have any effect on my breastfeeding. The community people say, if we eat proper food we will have breast milk and we can breastfeed. And there is no relation between anaemia and breastfeeding.

**Participant 9:** If the mother is anaemic she may have weakness and giddiness; so it is difficult for her to sit up and breastfeed her baby.

**Participant 7:** If the mother is anaemic the baby will not have good blood circulation.

**Moderator comment: Participant 1, 2 and 8 told that there is no effect on mother’s anaemia on breastfeeding. And the rest of the participants told that it has effect on breastfeeding and on the baby.**

1. **What does your community think of roles played by maternal overweight or obesity on breastfeeding?**

**Participant 1:** There is no problem in breastfeeding.

**Participant 2:** There is no problem in breastfeeding. But the frequency of breastfeeding will be less because it is difficult for her to sit up every time. (Other participants laugh)

**Participant 3:** Yes, there is problem in breastfeeding because it is difficult for her to sit up.

**Participant 4:** Even though it is difficult for the mother to sit up, she can have extra support of a pillow to her back and to the child so that the child will be at the level of chest and she can breastfeed.

**Participant 5:** There is an old saying in the community that “Is the fruit heavy to the vine?” So even though it is difficult to a mother she breastfeeds her baby. If she could not sit up she breastfeeds her baby in laying down position.

**Participant 6:** Even though it is difficult for an obese mother to breastfeeds her baby, with the help of family members she breastfeeds her baby. The community people say that it is difficult for an obese mother to breastfeed.

**Participant 7:** There is nothing more important for the mothers than their baby. So they take every effort to breastfeed.

**Participant 8:** Because of large size of breasts of obese mothers sometimes it is difficult for the baby to breath while breastfeeding as the breasts blocks the nose of the baby. But she takes the help of family members to hold the baby during breastfeeding.

**Participant 9:** It is difficult for the obese mother to hold the baby during breastfeeding.

**Moderator comment: All the participants said that it is a little difficult for breastfeeding the baby if the mother is obese and they said that the people in the community also say the same. Mainly it is difficult to sit up and hold the baby to breastfeed; but with the help of elders in the family in holding the baby they can breastfeed.**

1. **What does your community think of roles played by intimate partner violence on breastfeeding?**

**Participant 9:** Even though she is angry on her husband, she makes it sure that it doesn’t affect the breastfeeding of her baby. The people in the community also tell us to breastfeed the baby.

**Participant 8:** Even though there is quarrels between husband and wife, both of them take care of the baby. After the quarrel is over they get calm and the mother breastfeeds her baby.

**Participant 7:** We should not stop breastfeeding even if there is quarrel between husband and wife. She thinks that If she doesn’t breastfeed, the baby gets suffered. So in any condition she breastfeeds the baby. At the most she may stop breastfeeding for one or two hours. But later, she breastfeeds her baby.

**Participant 6:** If the husband and wife quarrel, sometimes the husband snatches away the baby from the mother and keeps it with him; but when the baby starts crying he gives it back to the mother for breastfeeding. The people in the community scold and counsel the husband so that he gives back the baby to the mother for breastfeeding.

**Participant 5:** In our community it is common to have quarrel between husband and wife. When they quarrel, the husband even though he is at fault he keeps the baby with him and asks the wife to go to her mother’s house and he doesn’t give the child back to the mother for two to three days.

**Participant 4:** Even though there is quarrel between husband and wife it should not become a problem for the baby.

**Participant 3:** If there is quarrel between husband and wife, the mother becomes mentally depressed. She may have less milk secretion and it will affect on her breastfeeding.

**Participant 2:** Even though there is quarrel between husband and wife, Two hours after the quarrel the mother breastfeeds her baby. People in the community say “Why you stopped breastfeeding your baby because of quarrel between you and your husband? Feed the baby.”

**Participant 1:** Even though there is a quarrel between husband and wife, the mother should breastfeed her baby, otherwise the baby will suffer.

**Moderator:** Do you all agree to this that the people in the community ask the mother to breastfeed her baby? Even though there is quarrel between husband and wife?

**All Participants (In chorus):** Yes…….

1. ***Acceptability of breastfeeding peer counselors (10 minutes)***
2. **Where do you get information about breast feeding?**

**Participant 7:** Through ASHAs, Staff nurses, Doctors, the elders in the community and our elder sisters who have delivered already; they tell us how to breast feed the baby.

**Participant 8:** Doctors, Nurses, Family members like mother and mother in law tell us.

**Participant 9:** Mother in law, Father in law and neighbours, tell us.

**Participant 6:** Nurses, my mother and mother in law and those who accompany the mother for delivery like neighbours.

**Participant 5:** All my family members, Doctors and Nurses tell us. Even though they don’t tell us I myself breastfeed the baby; it’s a god’s gift nobody need to tell me. (Laughs)

**Participant 4:** AHSAs, Doctors, Grandmother, my mother and Mother in law tell us about breastfeeding.

**Participant 3:** AHSAs, Doctors, Nurses, the elders in the family who have delivered earlier.

**Participant 2:** My mother and my elder sister told me about breastfeeding procedure.

**Participant 1:** Nurses, Doctors and my mother.

1. **If you have any breast feeding problem whom do you approach?**

**Participant 1:** I ask my mother.

**Participant 2:** I ask my neighbours

**Participant 3:** I ask my mother.

**Participant 4:** I ask my mother in law.

**Participant 5:** My mother and my grandmother

**Participant 6:** I ask my mother.

**Participant 7:** I ask ASHAs when they come for postnatal visit.

**Participant 8:** I ask my mother.

**Participant 9:** I ask my mother.

1. **Are you aware of any programmes by the government to promote breast feeding practices in the country?**

**Participant 7:** When we are pregnant mothers meetings are conducted at Anganwadi Centres by the health department of the government. In that meeting we are told about breastfeeding along with antenatal care.

**Participant 4:** In the mothers meeting ASHAs and ANMs tell us about breastfeeding. They arrange a meeting for pregnant women in the anganwadi where the doctor of the PHC and other health workers come and tell us about breastfeeding.

**Participant 1:** During Antenatal period we attend meetings arranged by ANMs and ASHAs where we learn about breastfeeding.

**Participant 2:** ANM and ASHAs arrange meeting for pregnant women where they tell us about nutrition and breastfeeding.

**Participant 3:** ASHA worker tell us about breastfeeding.

**Participant 5:** ASHAs and the staff in the hospital tell us about breastfeeding.

**Participant 7:** Every month mothers meeting are held in which we are told about breastfeeding.

**Participant 8:** At mothers meeting in PHC we are told about breastfeeding.

1. **Is there any support of the women from the village/community?**

**Participant 6:** My sister and neighbours come and help me.

**Participant 7:** Elders who have more experience than us.

**Participant 4:** Grandmothers those were mothers earlier help us.

**Participant 3:** Those who are elders to us, they have got all the experience. With their experience they tell us.

**Participant 7:** If there is any problem in breastfeeding anganwadi workers come and help us.

**Participant 1 & 2:** ASHA workers help us when they come as their routine visit. People who come to see the baby tell us about breastfeeding.

**Participant 8:** Grandmothers help us. My elder aunty used to tell us. She also used to conduct deliveries earlier.

**Participant 7:** Nobody comes exclusively to tell us about breastfeeding.

**Moderator 2:** In what other ways the community people support a breastfeeding mother? For example while traveling in a bus or when the mother is in a crowd of a fair.

**Participant 8:** They tell us to cover the chest and baby with saree while breastfeeding.

**Participant 5:** The neighbours tell us to come into their house and breastfeed the baby, when it is time to breastfeed. If the mother is not having breast milk the community people help her in giving top milk and top feeds.

**Participant 4:** They provide seat to the breastfeeding mothers while traveling in a bus. If the mother is breastfeeding in open sunlight they ask her to come into their house for breastfeeding.

**Participant 7:** The community people help the breastfeeding mother in all the ways so that there will not be any evil eye falling on the child.

1. **Is she trained in giving breast feeding support and help?**

**All Participants:** None of them are trained in breastfeeding.

**Breastfeeding peer attitude**

1. **Do you think that a person (Peer Counsellor) from your community/village, if trained will be helpful to solve the breast feeding problems?**

**All Participants (In chorus) :** Yes, it will be helpful.

**(Everybody raised their hands in acceptance and nodded yes)**

1. **Do you accept receiving Breast feeding counselling and advice from such a person?**

**All Participants (In chorus) :** Yes, we accept such person.

1. **What should be the qualifications of Peer Counsellor?**

**Participant 1:** She should have good hearted and well behaved. She should not become angry and should not scold.

**Participant 2:** She should not become angry while counselling the mother. She should counsel in a way so that the mother understands it on first occasion. Otherwise the mother keeps on asking her again and again till she understands or the mother may get frustrated and say “You can go, I have understood everything.”

**Participant 3:** She should be experienced.

**Participant 4:** She should have a clean heart and good attitude towards the baby. Even if we ask her anything again and again, she should tell us patiently.

**Participant 5:**  She should be experienced, learned and should have patience.

**Participant 7:** She should have the experience of breastfeeding herself and she should be able to tell about it to other mothers so that they can understand it properly. She should have patience.

**Participant 8:** She should have patience and should not become angry.

**Participant 9:** Even if the breastfeeding mother asks her repeatedly when she has not understood, the counsellor should explain her properly and patiently. If she becomes impatient and angry the mother also may become impatient and angry. If she tells us calmly we can understand better and breastfeed the baby in a better way.

**Participant 7:** She herself should be clean.

**Breastfeeding peer perceived control**

1. **What would be the benefit to have Peer Counsellor?**

**Participant 7:** We can protect our baby’s health, can prevent other diseases coming to out babies and if they tell us how to breastfeed properly, babies deaths can be prevented.

**Participant 6:** It is good for us if they tell us about breastfeeding. We can breastfeed better.

**Participant 5:** We would not be knowing how to breastfeed at the time of delivery. If she tells us about this, it will be helpful for us.

**Participant 4:** It will be good for the baby and the mother. The baby will be healthy.

**Participant 3:** It will be good for both mother and child.

**Participant 2:** When there is nobody else in the family, if they come and tell us about breastfeeding, it will be helpful for us and the baby will be healthy.

**Participant 1:** It will be good for the baby. If we don’t know about anything, we can learn it from her.

**Participant 9:** It will be beneficial.

**Participant 8:** It will be beneficial for those who have delivered for the first time. They can understand everything about breastfeeding.

**Moderator:** Will it be helpful in solving the problems of breastfeeding?

**All Participants (In chorus by raising their hands):** Yes.

1. **What would be the barriers/problems to have Peer Counsellor?**

**All Participants (In chorus):** There will be no problem.

**Breastfeeding peer social norm**

1. **Who approves the presence of Peer Counsellor?**

**All** **Participants (In chorus):** All will approve the presence of Peer counsellor.

1. **Who disapproves the presence of Peer Counsellor?**

**All** **Participants (In chorus):** Nobody disapprove the presence of Peer counsellor.

1. ***Program to train breastfeeding peer counselors (10 minutes)***
2. **How does the health worker help you in breast feeding activities?**

**Participant 7:** They come and tell us about breastfeeding when we are pregnant. They tell us to be clean and breastfeed the baby immediately after delivery. When they accompany us to the hospital for delivery then also they tell us. After delivery they come to visit us and tell us again about breastfeeding.

**Participant 6:** ASHAs come to our house to tell us about breastfeeding.

**Participant 5:** ASHAs come to our house to tell us about breastfeeding.

**Participant 4:**  ASHA workers tell us everything about breastfeeding.

**Participant 3:** ASHA workers tell us “As much as you breastfeed your baby, it would be good for you and your baby”. They tell us how to hold the baby and how to hold the neck of the baby while breastfeeding.

**Participant 2:** AHSAs tell us “Be clean and keep your baby also clean. If there is any bad smell the baby will have vomiting.”

**Participant 1:** They tell us “Take bath and keep yourself clean; otherwise the baby will have vomiting”.

**Participant 9:** They tell us to eat good food so that the baby will get good breast milk.

**Participant 8:** AHSA workers tell us to take care of baby and to breastfeed frequently.

1. **Does the health worker visit and advise you about Breast feeding before delivery?**

**Participant 9:** Yes, they tell us.

**Participant 8:** Yes, they tell us.

**Participant 7:** In mothers meeting they tell us to breastfeed immediately after delivery. They don’t visit us but they call us for the meeting and tell us.

**Participant 6:** In mothers meeting they tell us about breastfeeding.

**Participant 5:** They tell us during a meeting at anganwadi.

**Participant 4:** AHSAs tell us in a meeting at anganwadi. We understand and continue it.

**Participant 3:** AHSA workers tell us.

**Participant 1 & 2:** They call us to anganwadi and tell us.

**Participant 2:** It is not possible for them to tell us individually. Therefore they call us to anganwadi.

1. **What does the health worker talk about breastfeeding antenatally?**

All participants agree that one or the other health workers tell the mother about breastfeeding before delivery. The responses are already recorded under previous question.

1. **What happens at birth? Is she present at birth and help you with early starting of Breast Feeding?**

**All Participants:** Yes, they help us at the time of delivery for breastfeeding.

**Participant 1:** ASHA worker was with me at the time of delivery.

**Participant 2:** ANMs, ASHAs and my mother were with me at the time of delivery.

**Participant 4:** ASHA, Doctor, mother in law or grandmother will be there at the time of delivery.

**Participant 3:** Nurses, ASHAs, Grandmother or whoever elder is there in our family.

**Participant 5:** All help us. They help us in sitting up and holding the baby to breastfeed.

**Participant 6:** ASHAs and nurses help us in breastfeeding.

**Participant 7:** ASHA and nurses help us in breastfeeding.

**Participant 9:** ASHAs help us in breastfeeding.

1. **How frequently does she visit you after birth of the baby?**

**Participant 9:** Daily they come, our house is nearby. About 10 times they visit.

**Participant 8:** 7 to 8 times in six months.

**Participant 7:** 5 to 6 times in a month.

**Participant 6:** 7 to 8 times in six months.

**Participant 5:** 3 times in a month.

**Participant 4:** 7 to 8 times.

**Participant 3:** Once in a week.

**Participant 2:** 8 times.

**Participant 1:** 7 to 8 times.

1. **What is the purpose of visit?**

**Participant 1:** To see the mother and child.

**Participant 2:** To see whether the mother and child are kept clean.

**Participant 3:** To see the mothers health and to check the baby’s weight.

**Participant 4:** To say to keep the child clean, to breastfeed, to see whether the baby’s weight is increasing and to check the blood pressure of the mother.

**Participant 5:** To see whether the child is fine and whether the baby’s strength is improved or not.

**Participant 6:** To see the baby’s weight.

**Participant 7:** To examine the health of the child and to tell the mother to breastfeed frequently.

**Participant 8:** To see whether the child is kept clean and they also tell us to keep surrounding area clean.

**Participant 9:** To see whether the baby has gained weight.

1. **How frequently does she assess your breast feeding practices?**

**Moderator:** Do they observe your breastfeeding?

**All Participants (In Chorus):** Yes.

**Participant 9:** Approximately 2 times.

**Participant 8:** Whenever they come, they see.

**Participant 7:** 2 to 3 times.

**Participant 6:** Every time they visit they see.

**Participant 5:** Every time they visit they see.

**Participant 4:** 3 to 4 times.

**Participant 3:** 2 to 3 times.

**Participant 2:** 1 to 2 times.

**Participant 1:** 3 times.

1. **How many mothers does the health worker visit per day/per month?**

- Question not asked.

1. **What are the common breast feeding problems you observe in your community?**

- Already answered in Section A. Question 9.

1. **Do you think that peer counsellor who is from the same community would help and support mothers?**

**All Participants (In chorus):** Yes, it will be helpful.

**(Everybody raised their hands in acceptance and nodded yes)**

1. **What do you think would be the barriers for Peer Counsellors in the community?**

**Participant 8:** They may have difficulty to come to our houses daily. They have to finish their works in their home and also do this counselling work. They may have to leave their work in their home and do this work and it might be difficult for them.

**Participant 7:** It is their duty and they should do this work. It is inevitable for them. To do this work she has to leave any other work. There will not be any problem form the community; rather it is beneficial to the community.

**Participant 6:** There will not be any problem form community.

**Participant 5:** No problems form the community.

**Participant 4:** No problems form the community.

**Participant 3:** No problems form the community.

**Participant 2:** No problems form the community.

**Participant 1:** No problems form the community.

**Moderator comment: All participants agree that there will not be any problem from the community to the peer counsellor.**

1. **Will there be future opportunities for these Peer Counsellors to continue in the community?**

**Participant 7:** There will be opportunity, if the government gives them opportunities.

**Participant 6:** Yes, they have opportunity.

**All Participants (In chorus):** Yes, there is opportunity.

1. **What should be the criteria for acceptance of such counsellors by you and the community?**

- Already answered in Section B. Question 8.

1. **How do you identify and recruit such counsellors?**

**Participant 7:** We should arrange a meeting of elders in the village and talk to them. We need to ask the delivered women who have taken services from such persons. ASHAs are already familiar to the mothers. Likewise these women are also familiar to them. Anganwadi workers will help you.

**Participant 9:** Local elected members (Gram panchayat members) can also help in this matter.

**Moderator:** Who else can help in identifying peer counsellors?

**All Participants (In chorus):** ASHAs, Anganwadi workers and Health workers.

**F. Wrap up**

1. ***Ask participants***

**“How did you feel about participating in this session? What was easy? What was hard?”**

**All Participants:** We got knowledge and we felt like we also can help others. We can also tell others what we learnt here. So many things we learnt what we did not know earlier. By arranging such meetings in our community it is going to help us. This information is very much necessary for the mothers.

1. ***Summarize and thank participants by saying:***

**“We appreciate all of your great ideas. You have been a big help, and we want to thank you very much for all the information you have shared with us today. We know that your ideas will help to make this a successful program and technology. Thank you once again for your participation – we really appreciate you!”**

**Moderator: Thank you.**

**Focus group interview guide**

**Materials Needed:**

Food and Beverages

Newsprint & Markers

Masking tape

Nametags

Audiotape recorders and tape

Participant incentives

Questionnaires

Pencils

Attendance sheet

**A. Introductions and Focus Group Process (10 minutes)**

*1. Team members will have nametags on prior to participants entering the focus group venue*

*2. Give participants nametags as they arrive.*

*3. Instruct participants to select celebrity name*

*4. Have participants sign-in on attendance sheet.*

*5. Explain the purpose of the focus group session by saying:*

**“Welcome to today’s focus group. We are planning to develop mobile technology to support breastfeeding peer counselors and breastfeeding mothers. We asked you to come to today’s session because we would like to hear from you about your opinions and ideas on the technology content. You are the experts, and we can learn from you. We need your honest opinion – good and bad – about what we are trying to do. We would like you to share what you think – and what you think other members in your community might think about our project. Everything you are thinking is important to us. There are no right or wrong answers. We value your opinion. We would be very happy if you would help us to make the best technology possible.**

**Please remember to use your celebrity name and refer to others with their celebrity names. Also, try not to use any friends’ names or specific locations. But if you do, we will delete them from the audio recording.**

*As a reminder we are going to turn on the tape recorder now.*

*Is everyone ok with that? TURN TAPE ON: and announce*

“This is focus group # 6 on 05/04/2018 for the “BEST4Baby”

*1. Ask the participants to introduce themselves by saying their celebrity name and what is your favorite color.*

*2. All members of investigative team introduce themselves.*

**“Now, we would like to develop some group rules so we can learn as much as possible from each other.”**

**B. Group Rules (10 minutes)**

1. *Develop group rules to protect participants’ confidentiality. Offer examples of group rules if participants are stuck:*

Be respectful

Be honest

Maintain confidentiality

One person speaks at a time

Listen to others

No put downs or insults

Turn off all cell phones

**“Even though we will be talking about breastfeeding and technology development today, we do not require you to talk about your personal experiences with breastfeeding if you do not feel comfortable. You can instead share what you think or others’ experiences. However, please do not use any specific names or identifiable information of others for protecting their privacy. Is that clear to everyone?”**

**BREAST FEEDING EDUCATION SUPPORT TOOL for BABIES**

**Focus Group Discussion guide for Facility- and community-based Health Care providers**

**Discussion (40 Minutes)**

**“We are working to develop a mobile technology to support breastfeeding peer counselors and breastfeeding mothers in India. We want to know what you think and what you know about breastfeeding practices. Your inputs will certainly help develop feasible and effective technology to support breastfeeding. We have questions we prepared but we will let our group guide our own discussion. Let’s start with some initial questions about breastfeeding experiences.”**

**A. *Breastfeeding practice and support mechanism (20 minutes)***

1. Do all of the mothers in your community breastfeed?

**Participant 1:** All mothers breastfeed in our community.

**Participant 2:** All mothers breastfeed in our community.

**Participant 3:** All mothers in our community breastfeed.

**Participant 4:** All mothers breastfeed their babies in our community.

**Participant 5:** All mothers breastfeed in our community.

**Participant 6:** All mothers breastfeed their babies in our community.

**Participant 7:** All mothers breastfeed their babies in our community.

**Participant 8:** All mothers breastfeed their babies in our community.

**Participant 9:** All mothers breastfeed their babies in our community.

**Participant 10:** In our community, all mothers breastfeed their babies.

**Moderator comment: All participants told that all the mothers in their community breastfeed their babies.**

**Breastfeeding attitude**

1. Are there any circumstances that might arise that you can think of where it might be okay for you to encourage a mother not to breast?

**Participant 1:** Those mothers who are HIV infected don’t feed their babies and they should not breastfeed their babies. When the mother has breast abscess, retracted nipple or cracked nipple, she is unable to breast feed her baby.

**Participant 2:** I have not seen any such situation.

**Participant 3:** Only mothers who are HIV infected should not breastfeed their babies.

**Participant 4:** Those mothers who are mentally depressed do not have consciousness that she should breastfeed her baby.

**Moderator:** Does the mother has intention of not breastfeeding her baby?

**Participant 4:** No. (She meant the mother has intention to breastfeed her baby).

**Participant 5:** I have not come across any such situation.

**Participant 6:** During the initial period of my service there was a belief that, for the first three days after delivery, the colostrum which is thick, was not being fed to the babies.

**Participant 7:** I have not seen any such situation in our community.

**Participant 8:** I have also not come across any such situation in our community.

**Participant 9:** There was one woman in our community, who had three female children. In her forth delivery also, she had female baby. As she wanted a male baby, she neglected the fourth female child and did not breastfeed the baby on the first day after delivery.

**Participant 10:** I don’t know about any such situation.

1. How long does the mother need to breast feed exclusively and the total duration?

**Participant 1:** 6 months.

**Participant 2:** 6 months.

**Participant 3:** 6 months.

**Participant 4:** 6 months.

**Participant 5:** 6 months.

**Participant 6:** 6 months.

**Participant 7:** 6 months.

**Participant 8:** 6 months.

**Participant 9:** 6 months.

**Participant 10:** 6 months.

**Moderator:** What should be the total duration of breastfeeding?

**Participant 1:** Up to 1 year.

**Participant 2:** Up to 2 and half years.

**Participant 3:** Up to 2 and half years to 3 years.

**Participant 4:** Up to 2 years.

**Participant 5:** Up to 2 years.

**Participant 6:** Up to 2 years.

**Participant 7:** Up to 2 years.

**Participant 8:** Up to 2 years.

**Participant 9:** Up to 2 years.

**Participant 10:** Up to 2 years.

**Breastfeeding social norm**

1. Who approves breastfeeding around you?

**Participant 10:** In the hospital, where the mother delivers, the staff nurse tells her to breastfeed.

**Participant 9:** The staff nurse in the hospital and her mother or mother in law in the family tells her to breastfeed.

**Participant 8:** If the mother delivers in the hospital, the doctors or the staff nurse tells her to breastfeed. In the family, if she is in her mother’s house, her mother or elder sister advise her to breastfeed; and if she is in her husband’s house, her mother in law or other elders in the family tell her to breastfeed.

**Participant 7:** In the hospital, where the mother delivers, the staff nurse or doctor tells her to breastfeed. After she goes to her house, her mother or mother in law or other elders in the family advise her to breastfeed.

**Participant 6:** In the hospital, where the mother delivers the doctor and staff nurse tells her to breastfeed. In the community, her mother, other elders like grandmothers, anganwadi workers, ANMs or ASHA workers advise her to breastfeed.

**Participant 5:** In the hospital, the staff nurse advises her to breastfeed. In the community, when we (ASHA worker) visit her in her house advise her to breastfeed. And elders in her family like her mother, mother in law, elder sister and parents advise her to breastfeed.

**Participant 4:** If she delivers in the hospital, the staff nurse or the attenders who accompany her for delivery advise her to breastfeed. If she delivers in the home, experienced elders in the family advise her to breastfeed.

**Participant 3:** During the antenatal period of the mother, the doctors, staff nurses and anganwadi workers advise her about breastfeeding. After delivery, doctors, staff nurse, ASHA workers and in the house, her mother and elders in the community tell her to breastfeed.

**Participant 2:** In the hospital, where the mother delivers, doctors and the staff nurses tell her to breastfeed. In the family, her mother, mother in law and other elders advise her to breastfeed.

**Participant 1:** Doctors, staff nurses and mother in law advise her to breastfeed.

1. Who disapproves breastfeeding around you?

**All participants (In chorus):** There is nobody in the community who disapproves breastfeeding.

1. What do you and people around you think about prelacteal feeds?

**Participant 1:** According to my opinion, no prelacteal feeds should be fed to the babies. But few illiterate people feed honey, sugar water and cow milk during the first three days after delivery, thinking that there is no breast milk secretion during that period.

**Participant 2:** According to my opinion prelacteal feeds should not be fed to the babies but people in the community feed honey and sugar water to the babies as prelacteal feeds thinking that there will be no breast milk in the mother during the first 3 days.

**Participant 3:** According to my opinion no prelacteal feeds should be given to baby. Earlier, people in the community used to give honey and sugar water as prelacteal feeds to the babies. But now people also don’t like to give prelacteal feeds to the babies.

**Participant 4:** My opinion is, no prelacteal feeds should be given to the babies. But some people in the community feed prelacteal feeds like honey when the baby cries excessively so that the baby becomes calm.

**Participant 5:** Presently, nobody gives prelacteal feeds to the babies because the mother will be there in the hospital for 3 days after delivery and we see and observe that she breastfeeds her baby exclusively. And even in the community 80 percent of the people don’t like giving prelacteal feeds to babies.

**Participant 6:** Earlier, people in the community used to feed prelacteal feeds to babies but now there is awareness in the community that prelacteal feeds should not be given to babies. My opinion is no prelacteal feeds should be given.

**Participant 7:** My opinion is no prelacteal feeds should be given. But some people give prelacteal feeds to babies. They think that the mother has tired during delivery and has become too weak to breastfeed her baby and she needs rest. At that time they give some prelacteal feeds to the baby.

**Participant 8:** My opinion is prelacteal feeds should not be given. In the community some people still give prelacteal feeds to babies when the mother is sleeping. But this habit of giving prelacteal feeds is becoming less as we are educating the community.

**Participant 9:** I and the people in my community don’t like to give prelacteal feeds to the babies.

**Participant 10:** If the delivery occurs in the PHC, we (The staff in the PHC) don’t allow prelacteal feeds to the baby. But if the mother delivers at her home in the community, before we go to visit her, the family members give prelacteal feeds to the baby. They think that the prelacteal feed is good for the baby.

1. What do you and people around you think about colostrum?

**Participant 1:** It is very good for the baby. It gives immunity against many diseases. People also are in favour or feeding colostrum to the babies and nobody opposes it.

**Participant 2:** During antenatal period the mother is educated by doctor, staff nurse and ANMs to breastfeed the colostrum within half an hour after delivery. Nobody in the community opposes feeding colostrum to babies.

**Participant 3:** Now, there is awareness in the community that the colostrum has got immunity power and nobody opposes feeding it to babies. I am also of the opinion that it should be fed to babies.

**Participant 4:** I and the people in my community are of the opinion that the colostrum should be fed to babies.

**Participant 5:** I am of the opinion that the colostrum should be breast fed to the baby. Now there is awareness in the community that colostrum should be fed to babies.

**Participant 6:** We educate the people in the community to breastfeed the colostrum to babies and nobody in the community opposes to feed colostrum to babies.

**Participant 7:** I am of the opinion that the colostrum should be fed to babies because it increases the immunity of the baby. The people in the community also have been educated in breastfeeding the colostrum and they don’t oppose it.

**Participant 8:** Colostrum should be fed to babies within half an hour after delivery so that the baby becomes healthy. People also like to feed colostrum.

**Participant 9:** I and the people in my community like to breastfeed the colostrum to the babies.

**Participant 10:** I and the people in my community are in favour of feeding colostrum to babies.

1. What do you and people around you think about exclusive breastfeeding?

**Participant 1:** I and the people in my community are in favour of exclusive breastfeeding to babies.

**Participant 2:** People in my community have been educated and they breastfeed the babies exclusively for 6 months.

**Participant 3:** I and the people in my community are of the opinion that the babies should be exclusively breastfed for 6 months.

**Participant 4:** Now there is awareness in the community and the people like to breastfeed the babies for 6 months exclusively.

**Participant 5:** I and the people in my community are of the opinion that, for the first 6 months only breast milk should be fed to the babies.

**Participant 6:** I am of the opinion that, for the first six months the baby should be breastfed exclusively; and the people in the community are also of the same opinion.

**Participant 7:** I and the people in my community are in favour of breastfeeding the baby exclusively for six months.

**Participant 8:** Except in some special situation like twin babies, people prefer to breastfeed exclusively for six months. In case of twin babies, in addition to breast milk people give top milk, thinking that the mother’s breast milk is insufficient for both the babies.

**Participant 9:** Only in case of twin babies, people in the community give top milk or powder milk in addition to breastfeeding. Otherwise they give breastfeeding exclusively for 6 months.

**Participant 10:** I and the people in my community are in favour of exclusive breastfeeding for 6 months.

1. What do you and people around you think of gripewater /gutti /any medicines during the first six months?

**Participant 10:** Yes, within 3 months, people in the community give gripe water to babies. People think that if gripe water is fed to baby, the baby stays calm and do not become restless. They also think that it makes the baby to breast feed better and it improves its health. We tell the people not to feed gripe water to babies.

**Participant 9:** We tell the people not to feed gripe water and other things to babies; but people give gripe water and gutti made of dry date, almond and cashew nut. They think that it improves the weight of the baby.

**Participant 8:** People in the community give gripe water thinking that it improves the digestive power of the baby and easy passing of stools. They also feed paste of almond mixed with breast milk thinking that it improves the baby’s health.

**Participant 7:** If the baby cries excessively, the family members give gripe water to the baby, thinking that it makes the baby sleep well. People in spite of our advice not to feed anything to the baby, they give paste of almond, thinking that it improves the weight of the baby.

**Participant 6:** In spite of our advice of not to feed anything else than breast milk, people feed gripe water to babies thinking that it improves the digestive power of the baby. People also feed water to the babies.

**Participant 5:** Since 4 years in our community people don’t feed anything other than the breast milk to the babies. But before 4 years’ people used to feed nutmeg and cashew nut to babies thinking that it makes the baby sleep well. Now this habit is stopped.

**Participant 4:** We tell the mothers to drink more milk (she meant cow milk) so that the baby will get both water and breast milk in a better way. I don’t know about any other things being given to babies.

**Participant 3:** In our community nobody gives anything else other than breast milk to babies. We also advise them to feed breast milk only. And not anything else.

**Participant 2:** Even though we tell the mothers to feed breast milk only, and not to feed anything else, they feed gripe water one month after delivery, thinking that the baby sleeps well and will have good health.

**Participant 1:** The doctors in the hospital where the mother has delivered prescribe multivitamin, vitamin D and Phenergan drops to babies. I also prescribe the same medicines for the babies.

1. Why do the mothers think they do not have enough milk?

**Participant 1:** If the baby cries excessively, the mother thinks that the breast milk which she is having is insufficient.

**Participant 2:** No, I have not come across any such mother.

**Participant 3:** The grandmothers in the community tell the breastfeeding mothers that “There is sufficient milk in the mother given by god, even though it is not visible to our eyes". So, therefore people don’t believe that there is insufficient breast milk for the baby.

**Participant 4:** If the baby cries excessively and becomes weak, some mothers think that their breast milk is insufficient for their baby and they ask the doctor to prescribe powder milk and gripe water for their baby.

**Participant 5:** A few days back, a mother delivered in our hospital. For three days there was no breast milk secretion from that mother and the baby started crying excessively. The mother also thought that "Since I am not feeling my breasts heavy and painful, probably I am not having milk in my breasts".

**Participant 4:** I was also there with that mother during the first three days after delivery. I also tried to make the baby suck the breasts and also tried to express the milk from the breasts, but there was no milk coming from her breasts. We gave the baby to another mother for breastfeeding. And we referred the mother and the baby to paediatrician. Now she is having sufficient breast milk.

**Participant 6:** There was one mother in our community who had no breast milk secretion after one week of delivery. The elders in her family had told her not to drink more water and she was drinking less water. We met her and told her to drink more water and cow milk daily and we also told her about the position while breastfeeding. 2 to 3 days after doing so she started having sufficient breast milk.

**Participant 7:** If the mother is mentally depressed she may have insufficient breast milk.

**Participant 8:** Some family members don’t allow the mothers to drink enough water and to eat proper food. This leads to insufficient breast milk secretion. But the mother wonders "Why I am not getting enough breast milk?" and she approaches a doctor for this problem.

**Participant 9:** I don’t know any such situations.

**Participant 10:** If the delivery was difficult or if it was caesarean delivery, some mothers think that, they will not have sufficient breast milk. And some mothers think that if they are weak and don’t take nutritious food, they will not get sufficient breast milk.

1. What do you think will help woman to breastfeed longer, do exclusive breastfeeding?

**Participant 5:** We, the health workers and the elders like her mother, mother in law and grandmother in the family, need to advice the mother to drink more water and to eat nutritious food, so that she will breastfeed the baby properly.

**Participant 4:** We, all the staff nurses, ASHA workers and the family members should advise the mother to breastfeed her baby regularly and continuously. We should also advise her to drink more water and to have nutritious food with all types of vegetables.

**Moderator:** Who else should advice the mother?

**Participant 9:** ANMs also should advise.

**Participant 2:** We also (Anganwadi worker) advise the mother.

**Participant 8:** The women in the community who have experience of breastfeeding also advice the mother.

**Participant 3:** The mother should take nutritious food frequently and whenever the baby demands breast milk, she should breastfeed it. There should not be any time restriction for breastfeeding.

**Participant 6:** Whenever the baby wants to feed, the mother should breastfeed. By doing so, she will have more breast milk secretion. She should also take nutritious food like sprouted mung beans.

**Participant 7:** The mother should breastfeed her baby as and when it demands. If she breastfeeds more she will become hungrier and will take more food, which leads to more breast milk secretion. She should also eat all types of food items like sprouted mung beans, sweets, vegetables and should drink more water.

**Participant 8:** She should breastfeed frequently.

**Participant 9:** The mother should have fresh food always.

**Participant 10:** The mother should eat freshly prepared food and she should breastfeed the baby frequently.

1. Anything else you would like to add that you think is important?

**This question was not asked.**

**Breastfeeding perceived control**

1. What makes breastfeeding easier?

**Participant 1:** The mother should be seated in proper position in a well-ventilated room. And the baby also should be held in proper position while breastfeeding. The baby should suck the breast having both the nipple and areola in its mouth. The mother should wear comfortable dress.

**Participant 2:** The mother should not wear tight dress. She should sit in a comfortable position while breastfeeding. And during pregnancy, she should take nutritious food.

**Participant 3:** The mother should not have mental depression and there should not be any type of tension in the family. She should have a proper environment and clean place to breastfeed her baby.

**Participant 4:** The family members should give all the support to the mother to breastfeed her baby and she should be tension free. For the working mothers, there should be privacy for breast feeding at the work place. The environment should be quiet and peaceful. If there is more noise the baby gets scared and stops breastfeeding. And whatever participant one has told should also be there.

**Participant 5:** The mother should hold the baby properly, resting the baby’s head on her elbow so that it breastfeeds easily.

**Participant 6:** In addition to what participant 1 has said, the family members should give all the support to breastfeeding mother.

**Participant 7:** The mother should hold the baby while breastfeeding, so that the baby’s body should be touching the mother’s abdomen and the nipple and areola should be properly kept in the baby’s mouth. The family members should give all the support to breastfeeding mother.

**Participant 8:** The mother should be having good health. The place where she breastfeeds, should be calm and comfortable for both the mother and baby.

**Participant 9:** Sometimes, because of the bad smell of the mother’s sweat, the baby may not like to breastfeed. Therefore, the mother should keep her breasts clean always.

**(All other participants also agreed with participant 9)**

**Participant 10:** The mother should keep her breasts clean by wiping it with clean cloth every time after breastfeeding.

**Participant 6:** As for as possible the mother should breastfeed in sitting position only.

**Moderator:** How to make breastfeeding easy for a mother, when she is travelling in a public bus?

**Participant 4:** We should give her a seat to sit.

**Participant 3:** The persons sitting by her side should make space for her to breastfeed.

**Participant 5:** She should cover her baby with saree while breastfeeding.

**Participant 1:** For working women, sufficient period of maternal leave should be given.

**Participant 10:** The working mothers should breastfeed her baby before going to work and after coming from the work. In the middle time, her mother or father should give top feed to the baby in the house.

**Participant 3:** It is a problem for working mothers. It is my experience, that when I came back from the work and breastfed my baby, it used to vomit the milk.

**Participant 9:** Some mothers take their babies along with them to the work place.

**Participant 8:** Some mothers come to their house at the lunch hour and breastfeed the baby.

**Participant 8, 6 and 7(In chorus):** There is a place called rest room available for breastfeeding at working places.

**Participant 6:** It is there in only some places.

**Participant 5:** Now such places are made compulsory in all work places.

1. What makes breastfeeding harder?

**Participant 10:** Sometimes, there may not be a proper place for the mother to breastfeed her baby.

**Participant 9:** If the breasts are engorged it is difficult for the mother to breastfeed.

**Participant 8:** While travelling in a bus, it is difficult for a mother to breastfeed her baby. And it is also difficult while waiting in the bus stop.

**Participant 7:** It is difficult and embarrassing for the mother to breastfeed while travelling in a crowded vehicle. It is also difficult if the mother is suffering from cracked nipple or engorgement of the breast.

**Participant 6:** If the baby is not feeling well, it is difficult to breastfeed. And it is difficult to breastfeed in a crowded place.

**Participant 5:** When breast is engorged, it is difficult to breastfeed the baby. And it is difficult to breastfeed while traveling.

**Participant 4:** If the baby has cleft palate or any other illness it is difficult to breastfeed. And if the mother is under too much of work pressure, she may not breastfeed her baby in time.

**Participant 3:** There was a mother in our community who had a wound on her breast. Because of the wound she could not breastfeed properly. The wound was infected and she had abscess on both of her breasts which were operated in the hospital. During that time, she was unable to breastfeed the baby. And if the baby is suffering from cough, fever or breathlessness it is difficult for the baby to breastfeed.

**Participant 2:** I have not come across any such situation.

**Participant 1:** There are two types of problems; one is maternal, if there is excessive breast milk secretion it is risky for the baby because the baby may have milk aspiration. The other problem is, if the baby is not well, and if the breasts are large in size it is difficult for the baby to breastfeed.

1. What does your community think of roles played by maternal depression on breastfeeding?

**Participant 1:** No, it will not affect her breastfeeding.

**Participant 2:** Even though the mother is mentally depressed, if there is support for her in the family, the baby can be taken to her and can be breastfed.

**Participant 3:** Even though the mother is mentally depressed, there will be breast milk secretion as usual and with the help of family members, the baby can be breastfed.

**Participant 4:** It will cause some problem. If the mother does not breastfeed properly because of her mental depression, she may have swelling in her breasts. And if the baby does not breastfeed properly, the breast milk secretion also gets affected. I have seen one mother who was mentally depressed and was not breastfeeding properly.

**Participant 5:** Even though the mother is mentally depressed, if there is support from the family members, the baby can be breastfed properly.

**Participant 6:** If the mother is mentally depressed, she may have less breast milk secretion and she may not give adequate attention towards breastfeeding the baby; and the baby may lose weight.

**Participant 7:** The breast milk secretion becomes less.

**Participant 8:** The breast milk secretion becomes less and the mother may not feel like breastfeeding the baby.

**Participant 9:** Because of mental depression, the mother will not take adequate food; and her breast milk secretion becomes less. She may not breastfeed her baby on her own, but if somebody in the family helps her, she can breastfeed.

**Participant 10:** If the mother is mentally depressed, she will not give proper attention towards breastfeeding. This leads to less breast milk secretion.

1. What does your community think of roles played by maternal smoking on breastfeeding?

**Participant 1:** If the mother chews tobacco, there is no effect on her breast milk secretion or breastfeeding the baby.

**Participant 2:** There may be decrease in the breast milk secretion. There may be effect on baby also. The baby may have cough and sputum.

**Participant 3:** The mothers who chew tobacco will become thin and may not be active. Even though they chew tobacco, they are able to breastfeed their babies. It may have some effect on the baby also.

**Participant 4:** The child may become irritable due to the bad smell of the tobacco used by the mother. The baby may have vomiting and allergic symptoms due to the smell of tobacco. As the baby is very young, it may have some disease due to the use of tobacco by its mother.

**Participant 10:** If the mother chews tobacco, the baby may have cancer.

**Moderator:** How many of you agree with participant 10 who says if the mother chews tobacco her baby may have cancer? Please raise your hands to agree with him.

**(Nobody responds)**

**Participant 6:** The baby may not get cancer by the use of tobacco by its mother; but its immunity may become less. The breast milk secretion also will be less.

**Participant 5:** If the breastfeeding mother chews tobacco, it may have bad effect on her uterus and her baby may have breathing problems.

**Participant 7:** Some mothers who chew tobacco swallow it instead of spitting it out. In such cases it causes infection to the mother as well as to the baby.

**Moderator:** Does the tobacco chewing has any effect on breast milk production?

**All Participants except participant 1(In chorus):** Yes, it decreases breast milk production.

**Participant 8:** There is one mother in our community who chews tobacco regularly. She is not having breast milk secretion at all. Her baby is fed with top milk.

1. What does your community think of roles played by maternal drinking on breastfeeding?

**Participant 1:** If the mother takes alcohol, there is no effect on her breastfeeding. I have seen some fisher women taking alcohol after delivery to relieve the pain of child birth.

**Participant 2:** I have not seen or heard of any breastfeeding mother taking alcohol.

**Participant 3:** I have not seen or heard of any breastfeeding mother taking alcohol.

**Participant 4:** I have seen some breastfeeding mothers of a particular community, who take alcohol after delivery to get relief from pain and to have good relaxation. It may have some effect on breastfeeding as the mother goes into deep sleep and because of that, there may be disturbance in the regularity of breastfeeding. Even though the child cries for breastfeeding, she may not be aware of it under the influence of alcohol.

**Participant 5:** I have not seen or heard of any such things.

**Participant 6:** I don’t know about mothers taking alcohol. But I have heard about people giving a small quantity of alcohol to babies having cough and cold.

**Participant 7:** Due to consumption of alcohol by the mother, she becomes drowsy and there will be less emotional attachments with her baby. Even though the baby cries for breastfeeding, she cannot listen to it and some breastfeeding episodes may be missed due to that. Because of this, the baby may lose its weight.

**Participant 8:** Some breastfeeding mothers take alcohol because they think that it increases the hunger and appetite. But it doesn’t have any effect on breast milk secretion.

**Participant 9:** I have not heard anything about it.

**Participant 10:** I have not heard anything about it.

1. What does your community think of roles played by maternal drug use on breastfeeding?

**Participant 10:** I don’t know about any breastfeeding mother taking drugs.

**Participant 9:** I have also not heard.

**Participant 8:** I have also not heard.

**Participant 7:** I have not heard.

**Participant 6:** I have not heard.

**Participant 5:** I have not heard.

**Participant 4:** I have not heard.

**Participant 3:** I have not heard.

**Participant 2:** I have not heard.

**Participant 1:** Some mothers from fisher men community take drugs in small doses to increase the appetite and hunger.

1. What does your community think of roles played by maternal anaemia on breastfeeding?

**Participant 1:** The mother may be having weakness due to anaemia. But there will be no effect of anaemia on her breastfeeding.

**Participant 2:** Because of anaemia, the breastfeeding mother may have less breast milk production. The quality of the breast milk also will be poor.

**Participant 3:** There is no effect of anaemia on the breastfeeding. My own experience is that, I had chronic anaemia; but still I used to get enough breast milk for my baby.

**Participant 4:** Because of anaemia, the mother may have weakness; but her anaemia will not come in the way of her breast milk secretion or breastfeeding.

**Participant 5:** There will be no effect on breast milk secretion or breastfeeding if the woman is having anaemia.

**Participant 6:** If the mother is anaemic, her breast milk secretion will be less. And because of anaemia, she will be weak and cannot hold her baby properly while breastfeeding. This will lead to less breastfeeding to her baby than expected.

**Participant 7:** If the mother is anaemic, she will have less breast milk secretion. And because she doesn’t take adequate food and becomes weak, she cannot breastfeed her baby properly.

**Participant 8:** If the mother is having anaemia, her breast milk production will be less. As she becomes weak, she cannot hold and breastfeed her baby properly.

**Participant 9:** The maternal anaemia will not affect breastfeeding.

**Participant 10:** The maternal anaemia doesn’t have any effect on breast milk production. Because of her weakness, it may be difficult for her to take and hold the baby to breastfeed.

1. What does your community think of roles played by maternal overweight or obesity on breastfeeding?

**Participant 4:** If the mother is obese, she may not have good position to hold the baby and breastfeed. She will feel uncomfortable and tiredness while breastfeeding.

**Participant 7:** If the mother is obese, she will have more sweating and she may not be hygienic. Due to that she will have less emotional bonding with the baby.

**Participant** **6:** She has to breastfeed her baby in lying down position as for as possible. She cannot breastfeed in sitting position properly.

**Participant 3:** The obese mothers have more chances of undergoing caesarean section. Therefore, it is difficult for her to sit up and breastfeed her baby. Most of the time they breastfeed in lying down position.

**Participant 8:** Obese mothers are usually less active and they need the assistance of helpers in breastfeeding. They will have less breast milk production.

**Participant 2:** I don’t have any idea.

**Participant 1:** If the mother is obese, she may have less breast milk production. She may have difficulty in taking and holding the baby to breastfeed and it is difficult for her to have a good position for breastfeeding. And if she undergoes caesarean section, then also it is difficult for her to sit up and breastfeed.

**Participant 5:** The obese breastfeeding women will need a helper to take and hold the baby during breastfeeding. She cannot take and hold the baby on her own like the normal women do.

**Moderator:** How many of you agree that if the mother is obese, she will have less breast milk production? Please raise your hands.

**(Participant 1, 3, 4, 5, 8, 9 and 10** raised their hands.)

1. What does your community think of roles played by intimate partner violence on breastfeeding?

**Participant 10:** There is a popular saying in the community that “The baby suffered in the fight between husband and wife”. She neglects breastfeeding her baby even though it cries for breast feeding. Because she thinks that her husband has beaten her, even though she has done all her duties. Sometimes the husband takes away the baby with him for about one hour after the quarrel. During that time, the mother cannot breastfeed her baby.

**Participant 9:** Even though there is violence from the husband, the mother takes care of her baby and breastfeeds after the quarrel is over.

**Participant 8:** 50% of the mothers’ breastfeed their babies even though there is violence from her husband. The other 50% of the mothers neglect their breastfeeding because of the husband’s violence.

**Participant 7:** Some mothers neglect breastfeeding their babies because of IPV as she goes into depression and feels isolated from the husband and family members. But some mothers breastfeed their babies in spite of IPV, because they think that the baby should not suffer due to IPV.

**Participant 6:** Even though there is violence from the husband, the mother breastfeeds her baby because the emotional bond between the mother and the baby is very strong. Because of IPV she may not take food properly for a few days and her breast milk secretion may become less.

**Participant 5:** Whatever may be the violence from the husband, the mother doesn’t show her anger on the baby and she breastfeeds in any condition.

**Participant4:** Even if the husband beats her, the mother cannot withhold the breastfeeding when the baby cries.

**Participant 3:** Sometimes the husband may do violence on his wife. When the quarrel is over, the mother breastfeeds her baby. She doesn’t have any intention of not breastfeeding her baby.

**Participant 2:** Even if there is violence from the husband, the mother breastfeeds her baby. She gets attracted towards the care of the baby. She may stop breastfeeding for half an hour or one hour. But after that, she definitely breastfeeds her baby.

**Participant 1:** Because of quarrel between husband and wife, the mother may get depressed and the frequency of breastfeeding may come down.

**B. Acceptability of breastfeeding peer counselors (10 minutes)**

1. Where do you get information about breast feeding?

**Participant 1:** The mothers get information about breastfeeding from the elders in the family, community, from the mothers of her same age and from the media like television, newspapers, posters etc.

**Participant 2:** The mother gets information about breastfeeding from neighbours, anganwadi workers, and health care providers, parents and elders in the family.

**Participant 10:** Through public awareness, mothers meeting and from the community.

**Participant 9:** From her past experience in the previous deliveries.

**Participant 8:** The mother gets breastfeeding information from her mother.

**Participant 7:** She gets information from other mothers in the family who have breastfeeding experience.

**Participant 6:** The mother gets information about breastfeeding during antenatal clinics, nutritional programmes, health education programmes and through health education materials like banners and posters.

**Participant 5:** Through IEC (Information Education and Communication) activities conducted by government.

**Participant 3:** The mother may get information about breastfeeding through reading books.

1. If you have any breast feeding problem whom do you approach?

**Participant 10:** The mother approaches her mother if she has any problem in breastfeeding.

**Participant 9:** If there is any problem in breastfeeding, she approaches her mother first and then if necessary she approaches the hospital.

**Participant 8:** She approaches her mother, hospital and staff nurse.

**Participant 7:** She approaches her mother, mother in law and the doctor.

**Participant 6:** She approaches her mother, friends, ANMs, ASHA workers and doctors.

**Participant 5:** First she approaches her mother and if necessary as per her mother’s advice, she may go to doctors and clinics.

**Participant 4:** First she approaches her mother, and if necessary she goes to hospital.

**Participant 3:** First she approaches her mother in the family and if necessary she goes to a doctor.

**Participant 2:** She approaches her mother and elders and if necessary she approaches a doctor.

**Participant 1:** She approaches her family members, friends and family doctor.

1. Are you aware of any programmes by the government to promote breast feeding practices in the country?

**Participant 10:** I don’t know.

**Participant 9:** Recently the government has started a programme called MAA - Mothers Absolute Affection programme (A National Breastfeeding Promotion Programme).

**Participant 2:** We conduct programmes about breastfeeding in the month of August every year from 1^st^ to 7^th^ of August.

**Participant 5:** We conduct Breastfeeding week programme in the month of August.

**Participant 8:** MAA programme.

**Participant 6:** We conduct breastfeeding week programme from 1^st^ to 7^th^ of August every year.

1. Is there any support from the women from the village/community?

**Participant 8:** The community people provide seat to the breastfeeding women while travelling in a bus.

**Participant 2:** People provide place and privacy to the breastfeeding woman when she is outside her house like in a bus stop.

**Participant 3:** For breastfeeding mothers who go for work on daily wages, the employers help her by making facility for drinking water, giving her time for breastfeeding and they give her light work only.

**Participant 8:** The employers give her one hour time during lunch break for breastfeeding. The neighbours also take her child to her work place for breastfeeding.

**Participant 4:** Some people in the community take up her work for some time, so that she can breastfeed her baby during that time.

**Moderator:** When the mother goes for work do the other mothers in the family or in neighbourhood breastfeed her baby?

**Participant 8:** Yes, they can breastfeed if they have good relationship.

**Participant 2:** If they are sisters, they can breastfeed the baby.

**Participant 5:** If there are sisters both of whom are breastfeeding mothers, they can breastfeed each other’s baby. The breastfeeding mothers in the neighbourhood can also breastfeed the baby.

1. Is she trained in giving breast feeding support and help?

**Not asked this question.**

**Breastfeeding peer attitude**

1. Do you think that a person (Peer Counsellor) from your community/village if trained will be helpful to solve the breast feeding problems?

**All Participants except participant 1 (In chorus):** Yes, it will be helpful.

1. Do you accept receiving Breast feeding counselling and advice from such a person?

**All Participants (In Chorus):** Yes, if she is properly trained all the breastfeeding mothers accept receiving breastfeeding counselling and advice from her.

1. What should be the qualifications of Peer Counsellor?

**Participant 4:** She herself should be a mother.

**Participant 9:** She should have experience of breastfeeding.

**Participant 1:** She should have minimum education to read and write. (All participants agreed to this)

**Participant 5:** She should have patience to listen and advice the mothers.

**Participant 6:** She should have pleasant behaviour with smiling face; and she should have good communication skill.

**Participant 3:** She should have the ability of convincing the people who do not understand easily.

**Participant 4:** She should not get hurt if somebody comments on her; and she should be ready to convince them by telling repeatedly.

**Participant 8:** She should have passed at least 7^th^ or 10^th^ year of schooling. She should be acceptable to the people in the community.

**Participant 7:** She should have enough experience of breastfeeding so that she can educate the mother and answer if any questions are asked by the mother.

**Participant 6:** She should not discriminate the mothers on the basis of their caste.

(All participants agreed to the opinion of participant 6).

**Breastfeeding peer perceived control**

1. What would be the benefit to have Peer Counsellor?

**Participant 4:** The baby’s health improves and the mother will be happy. The baby’s immunity power will improve. The baby will not cry too much and the family will be happy.

**Participant 6:** The breastfeeding mothers will be able to understand when and how to breastfeed better. And they also receive information about how long to breastfeed.

**Participant 7:** In some families there may not be elders to advice the breastfeeding mother, for such mother the peer counsellor will be very beneficial and she will advise the breastfeeding mother like her own mother.

**Participant 2:** Both the mother and baby’s health will improve.

**Participant 3:** The breastfeeding mother need not go far away to get the information. She will get all the information about breastfeeding in her house only.

**Participant 8:** Some breastfeeding mothers may feel shy to tell even to her own mother about breastfeeding problems. To such breastfeeding mothers it will be very helpful to get help from peer counsellor.

**Participant 6:** It will be helpful for the both the breastfeeding mothers and health department in advising the breastfeeding.

**Participant 9:** The baby will grow better.

**Participant 10:** Both the mother and the child will have better care.

1. What would be the barriers/problems to have Peer Counsellor?

**All Participants (In chorus):** There will be no problems or barriers to have peer counsellor.

**Breastfeeding peer social norm**

1. Who approves the presence of Peer Counsellor?

**All Participants (In chorus):** All the people in the community approves the presence of peer counsellor.

1. Who disapproves the presence of Peer Counsellor?

**Participant 9:** Very rarely someone in the community may disapprove the peer counsellor.

**Participant 8:** About 3 to 4 persons in 100 may not approve the peer counsellor. They think that “we know whatever the peer counsellor is telling”.

**Participant** **7:** Some people think that “We don’t have enough time to listen to all that she is telling”.

**Participant 2:** Some women may think that “Why we should leave our work and listen to her? She just goes on telling us same things always”.

**Participant 3:** Some people think that “Why we should leave our work and listen to her?”

**Participant 4:** If they don’t approve, she should motivate them.

**Participant 5:** If she counsels them at their convenient time, they will approve. Some illiterate people may disapprove. And those people who have nobody in the family to help the breastfeeding mother and she herself has to do all the work may not approve.

**Participant 10:** Sometimes it is difficult to convince even the educated people because they think that they know everything and there is nothing for them to learn. It is easy to convince the uneducated people.

**Participant 8:** Some educated people don’t listen to others. Because they think that they know everything.

**Participant 5:** Such people are very rare, only 1 in 1000.

**Participant 4:** Some people think that “We have been to higher hospital and we know everything. There is nothing to learn from anybody”. Such people may not approve.

**Participant 7:** Some people, even though they have been to higher hospital they may not be knowing all the things about breastfeeding. It is only us who have to counsel them properly about breastfeeding then only they will understand about breastfeeding.

**C. Program to train breastfeeding peer counselors (10 minutes)**

1. Tell us about your practice on breastfeeding with women before they deliver.

-

1. What do you talk about breastfeeding antenatally?

-

1. What happens at birth?

-

1. How frequently do you visit the mother after birth?

**Participant 1:** I don’t visit breastfeeding mother’s houses, they only come to clinic.

**Participant 2:** I visit 7 to 8 times in 6 months.

**Participant 3:** I visit once in a week during antenatal period and after delivery, I visit to call them for mothers meeting.

**Participant 4:** I don’t visit the houses. (She is a staff nurse working in the PHC)

**Participant 5:** I visit 6 times during 6 months after delivery.

**Participant 9:** I go 12 times to visit during 6 months after delivery.

**Participant 6:** I visit 4 to 6 times during 6 months after delivery.

**Participant 8:** I visit 6 times during 6 months after delivery.

**Participant 7:** I don’t visit the houses. (She is a staff nurse working in the PHC)

1. What is the purpose of visit?

AND

1. How frequently you assess breast feeding practices of the mother?

**Participant 2:** I observe whether the mother is breastfeeding her baby properly or not. And we tell her to breastfeed properly and to maintain hygiene. I observe her breastfeeding technique once in a while and not every time I visit her.

**Participant 3:** We need to explain about breastfeeding every time when we visit those mothers who are not educated. For educated people we need not explain many times.

**Participant 5:** Even though they are educated, we need to explain them about breastfeeding every time we visit. We observe breastfeeding every time we visit. We also take the weight of the baby and observe their cleanliness of surrounding area.

**Participant 9:** We tell them to breastfeed frequently and in sitting position. We explain them about whatever is necessary to them. We enquire whether the baby is properly breastfeeding and the baby is passing the stools. We don’t explain every step of breastfeeding on each and every visit.

**Participant 5:** From the first day to seventh day, we enquire about her personal hygiene and whether the baby has passed urine and stools properly and whether she is drinking enough water.

**Participant 8:** We enquire her about her breastfeeding and if she is having any other problems.

**Participant 6:** We tell the mother to breastfeed her baby properly. Whenever we visit, we tell the mother about the position of breastfeeding and other things about breastfeeding. We tell them more about breastfeeding only.

1. How many mothers you have to visit per day/per month?

**Participant 10:** I visit 40 to 50 mothers in a month.

**Participant 9:** I visit 5 to 6 mothers in a month.

**Participant 8:** I visit 18 to 20 mothers in a month.

**Participant 6:** I visit 40 to 60 in a month.

**Participant 5:** I visit 10 to 15 mothers in a month.

**Participant 3:** I visit 10 to 15 mothers in a month.

**Participant 2:** I visit 20 mothers in a month.

**Participant 1:** About 6 to 10 breastfeeding mothers come to my PHC with breastfeeding problems.

1. What are the common breast feeding problems you observe in your community?

**Participant 1:** Because of unhygienic condition breast abscess formation and fever are very common. The breastfeeding women are very shy to tell their problems to the doctor.

**Participant 2:** I have not seen any breastfeeding problems.

**Participant 3:** If the babies are small they cannot feed all the milk present in the breast; some quantity of breast milk is left in the breasts which may lead to breast engorgement with fever.

**Participant 4:** The problems are common among the mothers who delivered for the first time. Cracked nipple and breast engorgement are the common problems in breastfeeding mothers. They also may not be knowing the correct procedure of breastfeeding, breast abscess is also common among these mothers. Because of faulty position of the mother during breastfeeding the baby may get suffocated due to pressing of the breast against the baby’s mouth and nose; and sometimes it may lead to death of the baby.

**Participant 9:** Swelling in the breast is also a problem in breastfeeding mothers.

**Participant 8:** If the mother has delivered by caesarean section she cannot sit up and breastfeed her baby.

**Participant 5:** Because of unhygienic condition and bad smell, vomiting of baby is also common.

**Participant 7:** Some babies are laid in the cradle immediately after breastfeeding without burping. These babies will have vomiting.

1. Do you think that peer counsellor who is from the same community would help and support mothers?

**All Participants (In chorus):** Yes, it will help.

(Even participant 1 agrees with other participants. He had disagreed earlier under question 6 of section B)

1. What do you think would be the barriers for Peer Counsellors in the community?

**Participant 5:** There may be some problems from the family members. And there may be quarrels between the family members and the peer counsellor.

**Participant 1:** There may be problem form society. It is difficult for the peer counsellor to work in the rural area.

**Participant 6:** There may be difference of opinion among the community members about the selection of peer counsellor based on the caste of the peer counsellor.

**Participant 2:** Some mothers may not listen patiently to the peer counsellor and they may show lack of interest in the counselling.

**Participant 7:** If the peer counsellor explains in very much detail the mothers may show less interest in listening to her. They may think “The peer counsellor is telling the same thing again and again”.

**Participant 8:** Some mothers may think that the peer counsellor discloses her personal information to others and may breach the confidentiality.

**Participant 6:** Agrees with participant 8 by nodding.

1. Will there be future opportunities for these Peer Counsellors to continue in the community?

**All Participants (In chorus):** Yes, there are future opportunities for the peer counsellor.

1. What should be criteria for acceptance of such counsellors by you and the community?

AND

1. How do you identify and recruit such counsellors?

**Participant 1:** She should be a local person of 30 years and above age. We should select a person with the help of local community.

**Participant 2:** She should be a local resident. We can identify such person with the help of persons working in the health system.

**Participant 4:** We should identify a person having good communication skill.

**Participant 8:** We need to identify such person with the help of community leaders.

**Participant 6:** We need to choose a person as peer counsellor with the help of ASHA, ANM, Anganwadi worker, local panchayat members and local leaders whose words are listen by the community. The local NGOs (Non-government organization) can also help in identifying peer counsellor.

**Participant 9:** We need to identify the peer counsellor with the help of ASHAs and panchayat members.

**Participant 5:** We should create the awareness about the need of peer counsellor so that eligible persons will come forward voluntarily to work as peer counsellor. We need to call about 4 eligible candidates. Among them we need to select one person who is most eligible.

**Participant 3:** We will identify eligible persons and bring them to you. You can select one among them.

1. Do you think training of peer counsellors is necessary?

**All Participants (In chorus):** Yes, it is good if she is trained in breastfeeding.

1. What should be the content of the training?

AND

1. What knowledge and skills should be taught to them?

**Participant 1:** The peer counsellor should give antenatal care, she should tell the mother to keep her nipple clean and she should visit during postnatal period and if the mother has retracted nipple peer the counsellor should advice the mother how to correct it. She should tell the mother not to wear tight dress. During postnatal period, she should tell the mother to have proper position while breastfeeding.

**Participant 4:** The peer counsellor should tell the mother about initiation of breastfeeding within half an hour after delivery. She should tell the mother about posture of breastfeeding and personal hygiene. She should tell the mother to have balanced diet. She should explain the mother about the importance of breastfeeding. She should explain the family members also to give proper support to the breastfeeding mother during her pregnancy and postnatal period.

**Participant 7:** She should tell the mother to breastfeed every 2 hours so that the baby’s weight increases. She should also tell the mother to keep the surrounding area of her house clean and to have personal hygiene. She should tell the mother burping after breastfeeding because it prevents vomiting after breastfeeding. She should tell the mother not to allow others to touch the baby because there may be chance of infection to the baby. She should tell the mother to expose her baby to sun light for 10 to 20 minutes every morning, till the baby becomes 1 month old. She should tell the mother to breastfeed from both sides, otherwise she will have breast abscess. If she gets breast abscess she cannot breastfeed her baby.

**Participant 5:** She should tell the mother that by breastfeeding she will not have breast engorgement. And by breastfeeding, there will be less bleeding during postpartum period. She should tell her to breastfeed at least 8 to 10 times per day. And to see whether the baby has passed urine at least 5 to 6 times in a day. She should tell to wash the hands before touching the baby. She should tell the mother about early ambulation.

**Participant 3:** She should tell the mother that by breastfeeding she will not have early conception. And it increases the emotional bond between the mother and child. She should tell the mother to take the baby on the solder after breastfeeding for burping. She should tell the mother not to breastfeed while she is sleeping.

**Participant 9:** She should tell the mother not to wear tight bra and to keep the baby clean. She should also tell the mother to breastfeed exclusively for 6 months.

**Participant 2:** She should tell her about personal hygiene and not to breastfeed in lying down position. She should tell her that compulsorily she should breastfeed her baby every 2 hours. She should advice the mother about spacing of pregnancy for at least 3 years. She should tell her to have nutritious food.

**Participant 6:** She should advise the mother about the care of the nipple and to breastfeed from both sides. She should tell the mother to have good ventilation in the house. If there is too much delay in breastfeeding the mother should throw away the fore milk and then breastfeed her baby. She should tell her that if she breastfeeds, the uterus will come to its original size. She should advice the mother to be mentally prepared before delivery and not to have any tension. She should tell her to feed the colostrum.

**Participant 8:** She should educate the mother not to feed prelacteal feeds like honey, sugar water and gripe water. And she should tell the mother to observe whether the baby is passing urine or not. She should tell the mother to feed both the fore milk and the hind milk. She should tell her to take care of her sutures if they are applied. She should advice the mother to breastfeed exclusively for 6 months.

**Participant 10:** She should tell the mother that as much she breastfeeds, both the baby and herself will be healthy.

**Participant 8, 5 and 4:** She should tell the mother not to chew tobacco.

**Participant 10 and 4:** She should tell the mother not to take alcohol and drugs.

1. Can we use mobile device to help Peer Counsellor counsel the mothers regarding optimal Breast feeding practice?

**All Participants (In chorus):** Yes, mobile app can be used for counselling by the peer counsellors.

1. How do you think using Mobile app would help Peer Counsellor?

**Participant 1:** Through app by showing pictures. The app can be downloaded from the peer counsellor’s mobile by the mothers and in turn they can share it to other mothers.

**Participant 3:** Through slides.

**Participant 6:** Through showing videos.

**Participant 8:** Through showing videos of mother breastfeeding her baby with proper procedure.

**Participant 4:** All the techniques of breastfeeding should be shown step by step through videos. And it should be shown what happens if proper breastfeeding is not done. Mobile is easy to carry and it can be used anywhere as it doesn’t require a large place.

**Participant 2:** We need to educate the community along with breastfeeding so that the people will understand the importance of breastfeeding.

**Participant 3:** Each step of breastfeeding should be explained separately.

**Participant 6:** Oral explanation is not much effective but visual explanation is more effective. (Participant 2 agrees with Participant 6).

**Participant 3:** Messages can be sent through mobile.

**Participant 9:** The mobile can be taken to the breastfeeding mother’s home where they are staying.

**Participant 10:** Through mobile app the level of public awareness about breastfeeding increases.

**Participant 3:** The experiences of breastfeeding mother can be recorded and saved in the mobile by peer counsellors. And it can be used to educate other mothers.

1. What will be the barriers using M-Health by the Peer Counsellor?

**Participant 8:** Using the mobile may have adverse effect on the baby’s brain.

**Participant 7:** Operating the mobile app is a challenge for the peer counsellor.

**Participant 6:** Charging the mobile and maintaining the charge is also challenge. If it is not charged properly it may not work.

**Participant 1:** The mobile can be misused for other purpose.

**Participant 2:** There may not be network accessibility in some areas.

**Participant 10:** There may not be proper internet connectivity in some places.

**Participant 10 and 8:** Some people in the community misunderstand and comment negatively about the use of the mobile app by the peer counsellor.

**F. Wrap up**

1. *Ask participants*

**“How did you feel about participating in this session? What was easy? What was hard?”**

**All** **Participants:** We felt good about participating in this group discussion.

1. *Summarize and thank participants by saying:*

**“We appreciate all of your great ideas. You have been a big help, and we want to thank you very much for all the information you have shared with us today. We know that your ideas will help to make this a successful program and technology. Thank you once again for your participation – we really appreciate you!”**

**Thank you.**

**Focus group interview guide**

**Materials Needed:**

Food and Beverages

Newsprint & Markers

Masking tape

Nametags

Audiotape recorders and tape

Participant incentives

Questionnaires

Pencils

Attendance sheet

**A. Introductions and Focus Group Process (10 minutes)**

*1. Team members will have nametags on prior to participants entering the focus group venue*

*2. Give participants nametags as they arrive.*

*3. Instruct participants to select celebrity name*

*4. Have participants sign-in on attendance sheet.*

*5. Explain the purpose of the focus group session by saying:*

**“Welcome to today’s focus group. We are planning to develop mobile technology to support breastfeeding peer counselors and breastfeeding mothers. We asked you to come to today’s session because we would like to hear from you about your opinions and ideas on the technology content. You are the experts, and we can learn from you. We need your honest opinion – good and bad – about what we are trying to do. We would like you to share what you think – and what you think other members in your community might think about our project. Everything you are thinking is important to us. There are no right or wrong answers. We value your opinion. We would be very happy if you would help us to make the best technology possible.**

**Please remember to use your celebrity name and refer to others with their celebrity names. Also, try not to use any friends’ names or specific locations. But if you do, we will delete them from the audio recording.**

*As a reminder we are going to turn on the tape recorder now.*

*Is everyone ok with that? TURN TAPE ON: and announce*

“This is focus group # 7 on 13 / 04 / 2018 for the “BEST4Baby”

*1. Ask the participants to introduce themselves by saying their celebrity name and what is your favorite color.*

*2. All members of investigative team introduce themselves.*

**“Now, we would like to develop some group rules so we can learn as much as possible from each other.”**

**B. Group Rules (10 minutes)**

1. *Develop group rules to protect participants’ confidentiality. Offer examples of group rules if participants are stuck:*

Be respectful

Be honest

Maintain confidentiality

One person speaks at a time

Listen to others

No put downs or insults

Turn off all cell phones

**“Even though we will be talking about breastfeeding and technology development today, we do not require you to talk about your personal experiences with breastfeeding if you do not feel comfortable. You can instead share what you think or others’ experiences. However, please do not use any specific names or identifiable information of others for protecting their privacy. Is that clear to everyone?”**

**BREAST FEEDING EDUCATION SUPPORT TOOL for BABIES**

**Focus Group Discussion guide for Members of breastfeeding mother’s support network (e.g., mother, mothers-in law, etc.)**

**Discussion (40 Minutes)**

**“We are working to develop a mobile technology to support breastfeeding peer counselors and breastfeeding mothers in India. We want to know what you think and what you know about breastfeeding practices. Your inputs will certainly help develop feasible and effective technology to support breastfeeding. We have questions we prepared but we will let our group guide our own discussion. Let’s start with some initial questions about breastfeeding experiences.”**

1. ***Breastfeeding practice and support mechanism (20 minutes)***
2. **Tell us about breast feeding experiences around you.**

**And**

**6. Do all of the mothers in the community breastfeed?**

**Participant 1:** In our community all mothers breastfeed, all mothers should breastfeed and breastfeeding is good.

**Participant 2:** Majority of the mothers breastfeed their babies but there are some women who do not breastfeed and they prefer top milk; but they are very few in number. They may be one in ten mothers.

**Participant 3:** All the mothers breastfeed their babies.

**Participant 4:** All the mothers breastfeed and I have not seen any mother giving top milk.

**Participant 5:** I have seen all the mothers breastfeeding. My own brother’s wife had delivered by caesarean section for the first three days after delivery I only help her to breastfeed her baby as she was unable to sit up.

**Participant 6:** I have also seen mothers breastfeeding.

**Participant 7:** I have seen mothers breastfeeding. I had also breastfed my daughter and the mothers in our community breastfeed.

**Participant 8:** Breastfeeding is important for the baby. It gives strength to the baby for its entire life. If the babies are breastfed they will be healthy.

**Participant 9:** All mothers in our community breastfeed and it is good for the baby.

1. Do you think breast feeding is important for the baby?

**Participant 1:** Breastfeeding is important and it is good for the baby.

**Participant 2:** Only breast milk is good for the baby.

**Participant 3:** Breast milk is very good for the baby. If anything else is fed, the baby will have vomiting.

**Participant 4:** Breastfeeding is very important for the baby.

**Participant 5:** Breastfeeding is good for the baby. It gives strength to the baby.

**Participant 6:** Breastfeeding is good for the baby. Whatever nutritious food we eat, it goes to the baby through breast milk.

**Participant 7:** Breast milk only is very good for the baby.

**Participant 8:** Breast milk is good for the baby and I had breast fed my son for ten years.

**Participant 9:** Breastfeeding is good for the children.

Moderator Comment: All participants said breastfeeding is good for the baby.

**Breastfeeding attitude**

1. Why? What are the benefits?

**Participant 9:** Top milk is not good for the baby. If the children are fed with top milk they may have bloating of the stomach and they may have diarrhoea. But if they are breastfed they babies will not have any of such problems.

**Participant 8:** With breastfeeding the baby will get strength and it prevents diseases to baby.

**Participant 7:** Mothers breast milk is good for the baby. If the baby is fed with top milk, it will have cough and cold. If the baby is breastfed it will not have such problems and it is good for the baby.

**Participant 6:** By breastfeeding the children will get all the nutritious ingredients in the food the mother eats. If the babies are given top feed, they will have problems.

**Participant 5:** By breastfeeding the babies will have good growth. If they are fed with top feed, they will have vomiting and diarrhoea. To feed the baby with top milk, one has to use bottle which may cause infection to the baby. This problem is not there with breastfeeding.

**Participant 4:** When the baby is breastfed, it is not seen by others and no evil eye falls on the breast milk and the baby takes sufficient quantity. If we give the top milk to the baby, we use bowl which can be seen by others then the baby may have evil eye and then baby will have vomiting. If the baby is breastfed it will have good growth. My own grandchild was given top milk for three days as the mother was having fever and was admitted in the hospital; the baby started having good growth only after initiating breastfeeding again.

**Participant 3:** If the baby is breastfed, it will have good growth. Otherwise its growth is hindered.

**Participant 2:** By breastfeeding it is beneficial to the baby as it gets all the nutrition in the breast milk. If the breast milk is fed to the babies frequently, they will grow properly. **Participant 1:** Breast milk is good for babies. If they are fed with top milk, they will have vomiting and diarrhoea.

1. If you think breastfeeding is not good, what would be the reasons?

**Participant 9:** When the mother is not well or when she has pain in the breast we tell her to feed goat's milk instead of breast milk.

**Participant 8:** When the mother is having fever the breastfeeding is decreased. Because the baby also may get fever.

**Participant 7:** My own daughter had cracked nipple and it was painful for her to breastfeed at that time. We consulted a doctor and were told to apply ointment to the nipple. Alternatively, we used to apply the ointment to the nipple on one side and breastfeed on the other side after cleaning it. Even though it was difficult the baby was breastfed from both breasts and we didn’t stop breastfeeding.

**Participant 6:** I have not come across such situations but very rarely when the mother is suffering from fever, the breastfeeding is stopped for one or two days.

**Participant 5:** When the mother’s breast become painful we have stopped breastfeeding for two to three hours only.

**Participant 4:** When my daughter was not well she had stopped breastfeeding her baby for two days. I have not seen any other mother in our community who has not breastfed her baby.

**Participant 3:** When the mother is having swelling in her breast the doctors advise her not to breastfeed for about four days because if she breastfeeds the blood and pus coming from the breast may enter the baby’s stomach. Therefore, in such condition the mothers are advised to give top feed.

**Participant 2:** I have not come across any such conditions where the breastfeeding is not advised.

**Participant 1:** Only when the mother is not well the breastfeeding is stopped because the baby also may have the mother’s disease. In such condition the baby is fed with top milk.

1. How long does the mother need to breast feed exclusively and the total duration?

**Participant 1:** For 5 months the mother should breastfeed exclusively and the total duration of breastfeeding should be up to 2 to 3 years along with top feed.

**Participant 2:** The mother should breastfeed her baby for 8 to 9 months exclusively and totally breastfeed the child for two years along with the top feed.

**Participant 3:** The mother should breastfeed her baby for 10 months exclusively and with top feed she should breastfeed her child for up to 1 and half to 2 years.

**Participant 4:** Exclusive breastfeeding should be done for 10 to 11 months and the total duration of breastfeeding should be for 2 years.

**Participant 5:** The mother should breastfeed exclusively for one year and totally she should breastfeed for 3 years.

**Participant 6:** For 6 months the mother should breastfeed exclusively and she should go on breastfeeding for 1 to 1 and half year duration.

**Participant 7:** For one year the mother should breastfed exclusively and the total duration of breastfeeding should be for 2 years.

**Participant 8:** We in the community breastfeed the babies exclusively for six months. Total duration of breastfeeding should be 3 years.

**Participant 9:** For 6 months we in the community exclusively breastfeed the babies and totally for 2 to 3 years the babies are breastfed.

**Breastfeeding social norm**

1. Do all of the mothers in the community breastfeed?

**All Participants (In chorus):** Yes, all mothers in the community breastfeed their babies.

1. Who approves breastfeeding around you?

**Participant 1:** In the hospitals the doctors tell the mother to breastfeed her baby. In the family we the elders, tell the mother to breastfeed.

**Participant 2:** Doctors tell the mother to breastfeed her baby. We the elders in the family also tell her. The mother herself should approve to breastfeed her child.

**Moderator 2:** Who gives permission for breastfeeding?

**Participant 1:** In the hospital it is a doctor who tells her to breastfeed her baby. In the family we the elders tell the mother to breastfeed.

**Participant 4:** The mother doesn’t take anybody’s approval. She herself starts breastfeeding her baby after delivery.

**Moderator 2:** Who tells the mother to breastfeed?

**Participant 4:** Her mother tells her to breastfeed.

**Participant 5:** Her mother.

**Participant 6:** Her mother.

**Participant 7:** Her mother.

**Participant 8:** Her mother.

**Participant 9:** Her mother.

1. Who disapproves breastfeeding around you?

**All Participants (In chorus):** Nobody disapproves for breastfeeding.

1. What problems did you encounter in breastfeeding?

**Participant 1:** When the mother has engorgement or swelling in her breast, it is a problem for her to breastfeed.

**Participant 2:** When there is engorgement of the breast and it is painful, it is difficult to breastfeed

**Participant 3:** When there is more milk in the mother’s breast then also it is difficult to breastfeed.

**Participant 4:** When the mother has fever she may have less milk secretion. This may cause a problem in her breastfeeding.

**Participant 5:** When the mother has fever she may have less milk secretion. And if the mother has swelling in her breast she may have blood and pus coming from her breast. Her breast milk also is not fit for feeding. And it should be expressed and thrown away.

**Participant 6:** I don’t know any such situations. Where the mother encounters problem in breastfeeding.

**Participant 7:** When the breasts become painful.

**Participant 8:** If the mother stops breastfeeding the breasts become engorged and it becomes a problem in breastfeeding.

**Participant 9:** When the breasts get engorged it is difficult for the mother to breastfeed.

1. What do you and people around you think about prelacteal feeds?

**Participant 9:** honey as prelacteal feeds.

**Participant 8:** We feed honey or sugar water as prelacteal feeds.

**Participant 7:** People say not to feed anything to the baby so we give only breast milk to the babies.

**Participant 6:** In earlier days the elders in the family like the grandmothers used to tell to give honey and sugar water as prelacteal feeds. But the present generation people don’t give anything as prelacteal feeds and we give breast milk only to babies.

**Participant 5:** People in our community tell us to give honey and sugar water as prelacteal feeds but we don’t give any such things. We give only breast milk.

**Participant 4:** In my family we have not given any prelacteal feeds to my grandchildren but one of my neighbour gave sugar water to the baby as the mother had no breast milk for five days. After five days a doctor prescribed her milk powder.

**Participant 3:** Only breast milk is good for the baby in my opinion. But some people in the community feed honey and sugar water as prelacteal feeds.

**Participant 2:** I have not given any prelacteal feeds in my family and I don’t know about the people in the community giving prelacteal feeds to babies.

**Participant 1:** After birth, if the baby is crying too much people give sugar water to the baby. And if the baby doesn’t cry they feed breast milk only.

1. What do you and people around you think about colostrum?

**Participant 1:** That milk is thick because it is there in the breast for nine months and I think it may cause problem to the baby; therefore we don’t feed it to the baby. We express it and throw it away. People also say not to feed it. Some people in the community tell us not to feed it.

**Participant 2:** We should feed the thick milk colostrum. People in our community also tell us to feed it.

**Participant 3:** We should not feed the thick milk colostrum. We are scared that it may cause some problem to the baby; therefore, we express the colostrum which comes first after delivery and we throw it away. People say to feed the colostrum.

**Participant 4:** We feed the milk which come after the colostrum. The colostrum should be fed to the baby, it is good. I have fed it to my children. People also tell to feed it. But earlier generation people tell not to feed it.

**Participant 5:** The colostrum should not be thrown away it should be fed to the baby. People in the community also tell to feed it.

**Participant 6:** It should be fed. It prevents diseases in the child. Nobody in the community tell not to feed it, but advice to feed it.

**Participant 7:** It should be fed. People in the community also advise to feed the colostrum.

**Participant 8:** It should be fed. People in the community also tell to feed it.

**Participant 9:** I have not fed the colostrum. People also tell us not to feed colostrum; they advise to give honey to the baby.

1. What do you and people around you think about exclusive breastfeeding?

**Participant 1:** I and the people in my community opine that for the first five months breastfeeding should be done exclusively.

**Participant 2:** The people in the community say that for the first 5 to 6 months, only breastfeeding should be done exclusively and nothing else should be given to baby.

**Participant 3:** For the first 5 months only breast milk should be fed. The people in the community say the same thing.

**Participant 4:** I and the people in the community are of the opinion that for the first 6 months exclusively breast milk should be fed.

**Participant 5:** I and the community say that exclusive breastfeeding should be given to the baby for the first 6 months.

**Participant 6:** I and my community are of the opinion that only breast milk should be fed exclusively for the first 6 months.

**Participant 7:** I am also of the opinion that for the first 6 months the baby should be breastfed exclusively. And the people in the community also say so.

**Participant 8:** Breast milk should be fed for the first 6 months exclusively.

**Participant 9:** Breast milk should be fed for the first 6 to 7 months exclusively. The people in the community also say the same.

1. What do you and people around you think of gripewater /gutti /any medicines during the first six months?

**Participant 1:** People in the community tell to feed gripe water and gutti to babies within first six months because they think that it is good for the babies. I also feel that it is good for the babies.

**Participant 2:** My opinion is that, till 6 months after delivery nothing of such things should be fed to babies. Now a day’s people also don’t feed such things to babies.

**Participant 3:** It is good to feed the babies with milk bottles; one bottle every month. If it is fed the babies will be calm. People in the community also say, “it is good for the babies if they are fed with dry dates and Jajikai (Nutmeg) mixed with breastmilk.

**Participant 4:** I have not given such things to our babies in the family. People in the community and our neighbours have fed gripe water to their babies; one bottle of gripe water for every month they used to give. People say that it is good for babies because they will be calm after feeding with dry dates. But I am of the opinion that such things should not be fed to babies during the first six months.

**Participant 5:** During the first six months such things should not be fed; if fed, it spoils the breast milk in the stomach of the baby. Dry dates and Nutmeg mixed with breast milk are also fed to babies.

**Participant 6:** People in the community tell to feed such things during the first month after birth so that the children who cry more will become calm. But I have not fed these things to the babies in our family and I am of the opinion that these should not be fed to babies.

**Participant 7:** I have not fed any such things to our babies and I am of the opinion that these should not be fed to babies. But people in the community tell to give such feeds to babies.

**Participant 8:** It is good to feed dry date paste to babies during the first six months. People in the community also think so. Dry dates are rubbed and mixed with breast milk to make a paste and it is fed to babies. We also feed herbal root and nut which prevents colicky pain and the children become healthy.

**Participant 9:** In our days we used to feed such things to babies. I don’t know whether the present day mothers feed such things or not. It is good to feed such things to babies.

1. Anything else you would like to add that you think is important?

**Breastfeeding perceived control**

1. What makes breastfeeding easier?

**Participant 1:** If the babies are healthy and strong they breastfeed properly and the mother gets relief in the breasts. Otherwise if the babies are weak they cannot feed breast milk adequately and the breast milk gets stored in the breasts and the breasts become engorged.

**Participant 2:** Immediately after birth the babies mouth is small and it breastfeeds slowly and cannot feed adequately. It is difficult for the mother to breastfeed. As the baby grows and become strong it breastfeeds adequately and there will not be any problem for the mother. To make the breastfeeding easy the baby should be breastfed frequently and continuously.

**Participant 3:** If there is more milk in the breasts it comes out while taking bath and while warming up the breast. If there are other small children in the family, they can be fed with extra milk so that it doesn’t get stored and so prevents breasts engorgement. Only if the babies breastfeed, it becomes easy for the mother.

**Participant 4:** The remaining milk in the breast after breastfeeding the baby should be expressed and thrown away; otherwise the mother will have swelling in her breast. After one or two months the baby become strong enough to feed all the breast milk and the mother will not have any problem.

**Participant 5:** The extra breast milk should be expressed and thrown away. The baby should be breastfed immediately after delivery.

**Participant 6:** The baby should be breastfed within 3 hours after delivery.

**Participant 7:** The mother should breastfeed her baby properly.

**Participant 8:** Warm water should be poured on the breasts to make them soft and there after the nipple should be put into the baby’s mouth. If the baby doesn’t suck the breast milk it should be expressed and fed to the baby.

**Participant 9:** The breasts should be washed and massaged with warm water, so that they become soft and easy for the baby to breastfeed.

1. What makes breastfeeding harder?

**Participant 4:** When the mother is having fever she may not be having enough strength to take and hold the baby to breastfeed. And also if she is suffering from fever, cough, cold or vomiting, she will take less food. And will have less breast milk. In such conditions it is a problem for the mother to breastfeed the baby.

**Participant 5:** When the mother had cough and cold or giddiness due to weakness, she cannot breastfeed the baby.

**Participant 3:** When the mother is suffering from fever or if she has delivered by caesarean operation, she is too weak to sit up and breastfeed the baby.

**Participant 2:** When the mother has delivered from caesarean section she is unable to sit up, at that time it is difficult for her to breastfeed.

**Participant 1:** When the breasts are engorged, it is difficult for the mother to breastfeed. At that time we need to express the breast milk and feed it to the baby or if it doesn’t take we have to throw it away.

**Participant 6:** When the mother has delivered by caesarean, it is difficult for her to breastfeed.

**Participant 7:** When the breasts have wounds and have become painful it is difficult to breastfeed.

**Participant 8:** When the mother has undergone caesarean section.

**Participant 9:** When the mother is ill it is difficult for her to breastfeed.

1. What does your community think of roles played by maternal depression on breastfeeding?

**Participant 5:** When a mother wants a male child and if she delivers a female child she becomes mentally depressed and she doesn’t show interest towards breastfeeding that female child. When she is mentally depressed she may have less breast milk secretion.

**Participant 4:** If the mother is mentally depressed she may have less breast milk secretion. She will not be happy and she doesn’t show interest in breastfeeding.

**Participant 3:** If anybody in her family gives her mental torture her breast milk secretion becomes less and as she doesn’t take food well, the breast milk may dry up. Even though she wants to breastfeed and tries to breastfeed she will not have breast milk in her. That in turn leads to more mental depression.

**Participant 2:** I tell you the example of my own daughter; she has 3 female babies and in her fourth delivery also she had female baby. She thought that "I have only female children in my fate". Her family members wanted a male child therefore she became mentally depressed and neglected the child by not breastfeeding properly. Her breast milk secretion also became less. After 3 years of my counselling now she has become mentally normal.

**Participant 1:** If the mother is mentally depressed, she will not be in a sound mind and she will not breastfeed properly even though there is breast milk in her. And if she doesn’t take food properly the breast milk gets burnt out.

**Participant 9:** If the mother is mentally depressed she will not take food properly and she will not have sufficient breast milk in her. She will not breastfeed properly.

**Participant 8:** If the mother is mentally depressed she will have less breast milk secretion.

**Participant 7:** If she doesn’t worry too much about the gender of the child she will breastfeed in a better way.

**Participant 6:** If the mother is mentally depressed, she will not take food adequately and her breast milk gets dried up. Even if she tries to breastfeed she will not secrete enough breast milk.

1. What does your community think of roles played by maternal smoking on breastfeeding?

**Participant 1:** If the mother chews tobacco, she will not have good quality breast milk and the baby may have problems.

**Participant 2:** If the mother chews tobacco both the mother and the child may get diseases like vomiting and burning in the chest.

**Participant 3:** If the mother chews tobacco, it goes into the baby’s body through breast milk. The child may get cough and the breast milk may dry up.

**Participant 4:** I don’t know anything much about breastfeeding mothers chewing tobacco. (Laughs)

**Participant 5:** I don’t know anything about breastfeeding mothers chewing tobacco.

**Participant 6:** If the mother chews tobacco, the baby may get cancer or heart problem.

**Participant 7:** One should not chew tobacco.

**Participant 8:** If the mother chews tobacco, she will get cancer and the baby also gets cancer.

**Participant 9:** Now a day’s mothers chew tobacco.

1. What does your community think of roles played by maternal drinking on breastfeeding?

**Participant 5:** People give fenny brand of alcohol to breastfeeding mother to relieve the pain. It may have some effect on breastfeeding.

**Participant 6:** If the mother drinks alcohol she gets intoxicated. The breast milk may burn out. It causes problems to the mother and babies also.

**Participant 9:** If the mother drinks alcohol she gets relief from body pain and get good sleep. The mother will have good breast milk secretion and it is good for the baby also.

**Participant 8: (Laughs loudly)**

**Participant 3:** Alcohol should not be given to breastfeeding mother. If she goes into sleep after drinking alcohol she may turn on towards the baby during sleep and may put her weight on the baby which will be a serious problem to a baby. I have seen one mother who had taken alcohol. During sleep she had turned on her baby which was sleeping by her side. The baby got suffocated and was struggling to breath because the mothers breast was pressing the baby’s mouth and nose. The grandmother of the baby noticed it immediately and push the mother away from the baby and the baby started breathing freely. The mother had no attention towards herself and towards her baby.

**Participant 4:** I don’t know anything about alcohol drinking.

**Participant 2:** Taking alcohol is not good for mother because it reduces breast milk secretion.

**Participant 1:** If cattle eats parthenium, the milk which it gives will also contain parthenium and it is not good for health. Likewise, if the mother takes alcohol her breast milk also contains alcohol and it is not good for the baby. The breast milk secretion also becomes less.

1. What does your community think of roles played by maternal drug use on breastfeeding?

**Participant 9:** It is not good for breastfeeding mothers.

**Participant 8:** I have not seen anybody taking drugs.

**Participant 7:** It is not good.

**Participant 5:** It doesn’t have any effect on breast milk.

**Participant 6:** It is not good. It reduces breast milk secretion.

**Participant 4:** It is not good. It spoils the breast milk and that milk is not good for the baby. The milk will not be tasty.

**Participant 3:** It is not good for the baby.

**Participant 2:** I don’t know anything about drugs.

**Participant 1:** I don’t know about it.

1. What does your community think of roles played by maternal anaemia on breastfeeding?

**Participant 1:** If the breastfeeding mother is anaemic she will have less strength and less breast milk secretion.

**Participant 2:** She will have less strength and less breast milk secretion. Therefore the baby will get less breast milk.

**Participant 3:** If the mother is anaemic she will not take food properly. Then the breast secretion also comes down.

**Participant 4:** She will have less food, less breast milk secretion. The baby gets less breast milk and it will have problem.

**Participant 5:** The anaemic mother eats less food, therefore she will have less breast milk secretion and the baby will not get adequate breast milk.

**Participant 6:** If the mother is anaemic she will have less breast milk and it will be a problem for the baby.

**Participant 7:** Yes, the mother will have less breast milk secretion.

**Participant 8:** If the mother is suffering from anaemia, she will have less breast milk secretion and her breast milk may burnout.

**Participant 9:** If the mother is anaemic, she will not have sufficient breast milk.

1. What does your community think of roles played by maternal overweight or obesity on breastfeeding?

**Participant 1:** It will be difficult for the mother to breastfeed.

**Participant 2:** If the mother is obese, she will have breast milk but it is difficult for her to breastfeed because of her obesity.

**Participant 3:** The obese mothers usually do not have breast milk and they cannot breastfeed.

**Participant 4:** The obese mothers have less breast milk and it is difficult for her to breastfeed as she is unable to move.

**Participant 5:** It is difficult for obese women to breastfeed and they will be having less breastmilk secretion. They will not get up and sit from the bed on their own and they cannot turn from one side to another easily on the bed.

**Participant 6:** If the mother is obese, she will be having less breast milk. Then the baby will have to suck more to get more breast milk due to which the mother tends to feed the baby with top milk using a bottle.

**Participant 7:** The obese mothers have less breast milk.

**Participant 8:** Some obese mothers have less breast milk. It is not difficult for them to breastfeed as they have enough strength to get up and breastfeed the baby.

**Participant 9:** The obese mother cannot eat adequate and they will have less breast milk. They give top milk in a bottle. They need some one’s help to sit up and breastfeed.

1. What does your community think of roles played by intimate partner violence on breastfeeding?

**Participant 8:** It will not have any effect on the mother’s breast milk secretion and she will breastfeed her baby in any condition because she loves the baby very much.

**Participant 9:** I am also of the same opinion.

**Participant 7:** I don’t have any idea about it.

**Participant 6:** It does not have any effect on breastfeeding. The mother breastfeeds her baby in any condition.

**Participant 5:** Breast milk will not become less if the husband of the woman beats her and she will breastfeed her baby.

**Participant 4:** Whatever may happen the mother will not stop breastfeeding her baby.

**Participant 3:** If there is IPV she will stop taking food and her breast milk secretion becomes less.

**Participant 2:** If the husband of the breastfeeding mother beats her she will have mental tension and she stops taking food. Therefore, she will not have adequate breast milk secretion.

**Participant 1:** Husband violence is only till they have food and sleep in the night. Afterwards the situation becomes normal.

***B.*** ***Acceptability of breastfeeding peer counselors (10 minutes)***

1. Where do you get information about breast feeding?

**Participant 1:** My grandmother and my mother in law used to tell me about breastfeeding. After learning from them we used to tell this to others. The information about breastfeeding is given by the staff in the hospitals.

**Participant 2:** Doctors tell us about breastfeeding. In our family we tell this to our daughters we were told by our mother in law and elders in our family.

**Participant 3:** Doctors and elders in the community tell us about breastfeeding. We give the same information to our daughter and daughters in law.

**Participant 4:** I was told by my mother in law. Now the doctors in the hospital and elders in the family give all the information about breastfeeding.

**Participant 7:** ASHA workers and ANMs give us the information about the breastfeeding.

**Participant 8:** ASHA workers and elders in the family tell about breastfeeding. We also get information about breastfeeding during group discussions.

**Participant 9:** Elders in the community father and mother in the family give the information about breastfeeding.

1. If you have any breast feeding problem whom do you approach?

**Participant 1:** We approach traditional birth attendant or ASHA workers. In earlier days we used to approach the elders in the community.

**Participant 2:** We approach the hospitals.

**Participant 3:** We approach the hospitals and ask ASHAs for help.

**Participant 4:** We ask ASHAs for help. And approach the hospital along with ASHA.

**Participant 5:** We go to hospital along with ASHA.

**Participant 6:** We approach ANM; according to their suggestion we go to hospital.

**Participant 7:** We ask ASHAs for help. We go to that hospital to which she suggests.

**Participant 8:**  We approach the hospital.

**Participant 9:** We also approach the hospital. In earlier days we used to approach the elders or whoever is having knowledge about it.

1. Are you aware of any programmes by the government to promote Breast feeding practices in the country?

**All Participants (In chorus):** No, we don’t know. (All raised their hands saying no)

1. Is there any support from the women from the village/community?

**Participant 5:** People in the community give eatables to breastfeeding mothers like food, fruits, dry dates, a porridge made of garden cress seeds, eggs and raw coconuts.

**Participant 9:** when the mother delivers in the hospital tea and biscuits are given by the people.

**Moderator probed the question:** Do anybody from the community help the mother if she is having any problem in breastfeeding?

**Participant 9, 1, 3 and 6 (In chorus):** Nobody comes and helps.

**Participant 8:** Elders in the community give suggestions to breastfeeding mother when she has breast engorgement. The woman who has the same name as that of the breastfeeding mother pour warm water on the chest and wave the locking chain of the door in front of her chest (A superstitious concept). Then the breast becomes soft and the mother breastfeeds her baby easily.

**Participant 5:** People enquire her whether she has adequate breast milk; whether she has breastfed her baby or not. Everybody in the community tell her to breastfeed.

1. Is she trained in giving breast feeding support and help?

**All participants (In chorus):** Nobody in the community is trained about breastfeeding.

**Breastfeeding peer attitude**

1. Do you think that a person (Peer Counsellor) from your community/village, if trained will be helpful to solve the breast feeding problems?

**All participants (In chorus):** Yes, it will be useful. (All participants raised their hands to say yes)

1. Do you accept receiving Breast feeding counselling and advice from such a person?

**All participants (In chorus):** Yes, we accept. (All participants raised their hands to say yes).

1. What should be the qualifications of Peer Counsellor?

**Participant 1:** Better if she is educated.

**Participant 2:** She should be educated.

**Participant 5:** She should be educated. She should have good character. She should be able to explain properly to the breastfeeding mothers.

**Participant 8:** She should be clever and should know what happens when something is told to people. If she understands this, she will be accepted by the community.

**Participant 4:** If she is educated she will be having some knowledge. She should be able to give information.

**Participant 3:** She should explain all the things about breastfeeding.

**Participant 2:** She should be knowledgeable.

**Participant 1:** She should be knowledgeable and educated. If she explains us in a better way we learn easily. She should be like a ASHA or ANM.

**Breastfeeding peer perceived control**

1. What would be the benefit to have Peer Counsellor?

**Participant 2:** We and the community will be improved and the children's health also will be improved.

**Participant 3:** Children’s health will be improved. The person who do not know everything will also understand about breastfeeding and other things.

**Participant 4:** Some women do not have the knowledge about breastfeeding. If the peer counsellor explains her about it she will understand. Some breastfeeding mothers may not be listening to the elders in the family. For such mothers if the peer counsellor comes and explains about breastfeeding, she will listen to her and understand.

**Participant 5:** Some breastfeeding mothers get engaged too much in household works neglecting breastfeeding the baby. If the peer counsellor tells her she will first breastfeed her baby and then she will attend the household work and it is good for the child.

**Participant 6:** She should not be short tempered. She should explain everything patiently. It is beneficial because she gives knowledge to the breastfeeding mothers.

**Participant 7:** It is useful to us and to the community as well.

**Participant 8:** The community improves. The women will have more knowledge. The babies will be better breastfeed and will become healthy.

**Participant 9:** She should be having good knowledge and should be able to explain everything. If she is old aged she herself will be forgetful.

1. What would be the barriers/problems to have Peer Counsellor?

**Participant 5:** There will not be any problem of any type for us.

**Participant 6:** There will not be any problem.

**Participant 8:** If somebody interferes in the work of peer counsellor, we need to approach the local panchayat and get it solved. We need to tell the members in the panchayat that “The peer counsellor had gone to a family to tell about breastfeeding and someone in the family is objecting for this. Please come and solve the problem”. Some arrogant men or alcoholic men may be there in the family who may ask the peer counsellor “Why you have come to our family”. And this will create a problem. We should not react to such persons. We should neglect such persons wisely and go ahead with our work. If we indulge in argument with them, there will be a tension.

**Participant 3:** If we, all the women get united, there will be no problem. If there is any problem, we can discuss about it and solve it.

**All participants (In chorus):** There will be no problem to have a peer counsellor and there will not be any problem from her to the community.

**Participant 5:** Very few people may not allow the peer counsellor into the worship place in their house (Where they do worship their idols of gods) if she belongs to a lower caste in the community. But if we convince the family members that she has come to counsel about breastfeeding they will allow her in their house.

**Breastfeeding peer social norm**

1. Who approves the presence of Peer Counsellor?

**Participant 5:** We need to ask the elected chairman and the elders in the community. There is one old man in our village who approves. We need to discuss about this with the elders at a meeting in Anganwadi centre.

**Participant 9:** Elected members of the local panchayat and community leaders.

**Participant 8:** If some four people like us give approval there is no need to take anybody else’s approval. And all the people in the community will give approval for peer counsellor.

**Participant 7:** Elders in the community give approval.

**Participant 4:** All the members of the local panchayat will approve.

**Participant 3:** There is a chairman in our village. We need to ask him and panchayat members.

**Participant 2:** My brother himself is the chairman. Our community is a closely knit community.

1. Who disapproves the presence of Peer Counsellor?

**All Participants (In chorus):** Nobody disapproves.

1. **Program to train breastfeeding peer counselors (10 minutes)**
2. How does the health worker help you in breast feeding activities?

**Participant 8:** ASHAs give us information. They tell to breastfeed the baby immediately after delivery. If the mother is in some problem and delay breastfeeding for some time; if baby cries during that time they give honey or sugar water to babies till the mother starts breastfeeding. The health workers help in cleaning the breast with warm water, massaging and breastfeeding.

**Participant 9:** ASHAs advice the mother to breastfeed the baby.

**Participant 7:** ASHAs come and tell the mothers to breastfeed the baby.

**Participant 5:** ANMs also come and tell the mothers to breastfeed the baby’s. they also help in correction the nipples if they are retracted.

**Participant 4:** They tell all the techniques of breastfeeding if the baby doesn’t breastfeed.

**Participant 3:** They tell about hand washing, taking bath and if the nipples are retracted how to correct it and how to put it into the baby’s mouth.

**Participant 2:** Our ASHA is also good. She tells about everything about breastfeeding and other things.

**Participant 1:** Our ASHA also tells everything about how to breastfeed the baby.

1. Does the health worker visit and advise you about Breast feeding before delivery?

**All Participants (In Chorus):** Yes, they tell about breastfeeding before delivery.

1. What does the health worker talk about breastfeeding antenatally?

**Participant 5:** The health worker gives advice about proper taking of food, drinking enough water and regular check-ups at hospitals. Sometimes the ASHA herself takes the pregnant woman to the hospital for check-up. She tells about breastfeeding also; how to wash the breast and feed the baby.

**Participant 8:** During antenatal period the health workers tell the mother to eat sprouted cereals (Madike kalu), green leafy vegetables.

**Participant 1:** She does not tell about breastfeeding during antenatal period.

**Participant 3:** The health workers tell about nutrition, walking and regular health check-ups during antenatal period. They don’t tell anything about breastfeeding.

**Participant 4:** They tell about having nutritious food. They don’t tell about breastfeeding before delivery.

**Participant 5:** They don’t tell about breastfeeding before delivery. They tell about it after delivery.

1. What happens at birth? Is she present at birth and help you with early starting of BREAST FEEDING?

**Participant 8:** The health workers help the mother in washing the breasts putting the breast to the baby’s mouth and breastfeeding.

**Participant 1:** They put the baby on the mother’s chest. Afterwards they give the baby to us. If the baby cries they tell the mother to breastfeed or to give sugar water to the baby.

**Participant 2:** ASHA worker tells the mother to clean the breast and feed the baby. They also tell to give breast milk only and not anything else like sugar water. She takes the baby to the mother and asks her to breastfeed. She herself helps the mother to sit and breastfeed the baby.

**Participant 3:** She asks the mother to sit-up and breastfeed the baby. If there is wound, she asks her to lie down on one side and breastfeed.

**Participant 4:** The health workers tell the mother to wash her breasts cleanly and put the nipple in the baby’s mouth to breastfeed properly.

**Participant 5:** They help the mother in all the way to breastfeed the baby. They help her in putting the nipple to the baby’s mouth and how to breastfeed the baby.

**Participant 6:** The ASHA and health workers give the baby to the mother, puts the nipple in the baby’s mouth and ask her to breastfeed.

**Participant 7:** The health workers teach the mother how to breastfeed the baby.

**Participant 9:** ASHAs take care of the breastfeeding mothers. If the baby cries, she tells the mother to breastfeed the baby. They make sure that the mother has breasted the baby.

1. How frequently does she visit you after birth of the baby?

**Participant 5:** Once in a week or once in fortnight they come. In 6 months they come about 10 to 15 times.

**Participant 4:** During the first 6 months after delivery they come for at least 6 to 7 times.

**Participant 3:** They come about 4 to 5 times.

**Participant 2:** ASHAs stays near my house, she comes daily.

**Participant 1:** Yes, daily she comes.

**Participant 6:** She comes once in 2 days or once in 4 days.

**Participant 7:** She comes 7 times in 6 months.

**Participant 8:** About 4 to 5 times in 6 months.

**Participant 9:** 7 to 8 times they come.

1. What is the purpose of visit?

**Participant 9:** She asks whether the breastfeeding mother is fine. She tells her to eat green leafy vegetables and sprouted cereals ( Madike kalu) and to give gutti to children.

**Participant 8:** If the baby has not breastfed, she asks the mother to breastfeed.

**Participant 7:** She asks about delivered women and children. She asks the mother to breastfeed the baby properly in sitting position. She tells her not to breastfeed in lying down position.

**Participant 6:** She tells the mother to breastfeed the baby and to get her baby vaccinated.

**Participant 5:** She asks the mother whether she is fine and whether she is breastfeeding properly. She also checks the weight of the baby.

**Participant 4:** She asks about the mother and baby’s condition and tells her to take food properly and to breastfeed the baby properly.

**Participant 3:** She tells the mother to take good care of the baby, to breastfeed properly.

**Participant 2:** She tells her about breastfeeding and to take care of the baby.

**Participant 1:** She checks the weight of the baby once in fortnight or once in a month and she also checks the BP of the mother.

1. How frequently does she assess your breast feeding practices?

**Participant 5:** The mother should cover the baby with her saree when breastfeeding. The mother should hold the baby in proper position. They also tell to keep the areola part of the breast in the baby’s mouth. She tells to wipe and keep the breast clean. Whenever she comes she observes the process of breastfeeding.

**Participant 6:** To wipe the breast and keep it clean. They also tell her to breastfeed the baby properly**.** She tells to clean the baby’s mouth after breastfeeding.

**Participant 7:** To hold the baby on the chest after breastfeeding and to rub the back (till Burping).

**Participant 4:** The ASHA worker observes the mother and asks her to show how she breastfeeds her baby every time she visits her. She does this about 5 to 6 times.

**Participant 6:** Yes, she observes every time she comes to visit her.

**Participant 7:** Whenever she comes to visit the mother she observes her breastfeeding.

**Participant 8:** Each time she visits; she observes the baby breastfeeding.

**Participant 3:** Whenever she visits, she observes the breastfeeding of the mother.

**Participant 1:** I am also of the same opinion.

1. How many mothers does the health worker visit per day/per month?

**Participant 1:** I don’t know about it. Nobody has come to our house.

**Participant 2:** There is only one ASHA to our village. She visits two or three mothers daily.

**Participant 3:** She visits as many mother as those who have delivered.

**Participant 5:** 10 to 15.

**Participant 7:** 5 to 6 women.

**Participant 6:** She visits all the delivered mothers. I don’t know the exact number.

**Participant 8:** 4 to 5 mothers.

1. What are the common breast feeding problems you observe in your community?

**It is already answered in Section A. Question 9.**

1. Do you think that peer counsellor who is from the same community would help and support mothers?

**All participants (In chorus):** Yes, it will be helpful.

1. What do you think would be the barriers for Peer Counsellors in the community?

**All participants (In chorus):** She will not have any barriers. We will see to it that there will not be any barriers for her.

1. Will there be future opportunities for these Peer Counsellors to continue in the community?

**All participants (In chorus):** Yes.

**Participant 2:** We will all help her. The government should give her salary.

**Participant 3:** The government should give her salary.

**Participant 6:** People in the community will help her. Nobody gives her much salary; they may give her food grains and vegetables which they grow in their farms. The government should give her salary.

**Participant 8:** The government may give her salary.

**Participant 4:** The government should give her salary.

1. What should be criteria for acceptance of such counsellors by you and the community?

**Participant 8:** If we have understanding all the community will accept her. If we go wrong nobody will accept her.

**Participant 5:** She should have good characters.

**Participant 8:** She should have good contact with the people.

**Participant 7:** She should be having good knowledge.

**Participant 2:** If she knows everything we accept her and she also accepts us.

**Participant 5 and 6:** She should be from our village only.

**Participant 6:** Some people do not respect the person from their own village but respect a person from outside.

**Participant 3:** We should get adjusted and procced

**Participant 2:** All should agree.

1. How do you identify and recruit such counsellors?

**Participant 5:** The government should give training to them.

**Participant 6:** You doctors and ANMs only should train them.

**Participant 2:** You only (Doctors) should train them.

**Participant 5:** To identify you should ask ASHAs.

**Participant 6:** ASHAs can ask the people to gather and discuss about it. After discussing we can get information from them.

**Participant 7:** ASHAs are there, you can ask them.

**Participant 2:** My own brother is there as chairman in local panchayat. There are other wise and elder people also in the community.

**F. Wrap up**

1. *Ask participants*

**“How did you feel about participating in this session? What was easy? What was hard?”**

**All participants said it is good.**

**Participant 1:** We understood so many things which we have forgotten.

**Participant 2:** It will be good for us. We became wiser.

**Participant 3:** We understood so many things again.

**Participant 4:** We learnt many things.

**Participant 5:** I recalled my experiences of forth delivery I attended. We got more knowledge so that we can tell this to others.

**Participant 6:** It became useful for us to tell our daughters and daughter in law who are going to deliver in future, how to breastfeed.

**Participant 7:** My daughter has delivered recently; it is useful for me to tell her how to breastfeed.

**Participant 8:** We appreciated whatever we have discussed with you.

**Participant 9:** We are happy with your discussion.

1. *Summarize and thank participants by saying:*

**“We appreciate all of your great ideas. You have been a big help, and we want to thank you very much for all the information you have shared with us today. We know that your ideas will help to make this a successful program and technology. Thank you once again for your participation – we really appreciate you!”**

- **Thank you**

**Focus group interview guide**

**Materials Needed:**

Food and Beverages

Newsprint & Markers

Masking tape

Nametags

Audiotape recorders and tape

Participant incentives

Questionnaires

Pencils

Attendance sheet

**Introductions and Focus Group Process (10 minutes)**

1. *Team members will have nametags on prior to participants entering the focus group venue*
2. *Give participants nametags as they arrive.*
3. *Instruct participants to select celebrity name*
4. *Have participants sign-in on attendance sheet.*
5. *Explain the purpose of the focus group session by saying:*

**“Welcome to today’s focus group. We are planning to develop mobile technology to support breastfeeding peer counselors and breastfeeding mothers. We asked you to come to today’s session because we would like to hear from you about your opinions and ideas on the technology content. You are the experts, and we can learn from you. We need your honest opinion – good and bad – about what we are trying to do. We would like you to share what you think – and what you think other members in your community might think about our project. Everything you are thinking is important to us. There are no right or wrong answers. We value your opinion. We would be very happy if you would help us to make the best technology possible.**

**Please remember to use your celebrity name and refer to others with their celebrity names. Also, try not to use any friends’ names or specific locations. But if you do, we will delete them from the audio recording.**

*As a reminder we are going to turn on the tape recorder now.*

*Is everyone ok with that? TURN TAPE ON: and announce*

“This is focus group # 8 on 03 / 07 / 2018 for the “BEST4Baby”

*1. Ask the participants to introduce themselves by saying their celebrity name and what is your favorite color.*

*2. All members of investigative team introduce themselves.*

**“Now, we would like to develop some group rules so we can learn as much as possible from each other.”**

**Group Rules (10 minutes)**

1. *Develop group rules to protect participants’ confidentiality. Offer examples of group rules if participants are stuck:*

Be respectful

Be honest

Maintain confidentiality

One person speaks at a time

Listen to others

No put downs or insults

Turn off all cell phones

**“Even though we will be talking about breastfeeding and technology development today, we do not require you to talk about your personal experiences with breastfeeding if you do not feel comfortable. You can instead share what you think or others’ experiences. However, please do not use any specific names or identifiable information of others for protecting their privacy. Is that clear to everyone?”**

**BREAST FEEDING EDUCATION SUPPORT TOOL for BABIES**

**Focus Group Discussion guide for**

**Mothers who had problem in exclusive breastfeeding**

**Discussion (40 Minutes)**

**“We are working to develop a mobile technology to support breastfeeding peer counselors and breastfeeding mothers in India. We want to know what you think and what you know about breastfeeding practices. Your inputs will certainly help develop feasible and effective technology to support breastfeeding. We have questions we prepared but we will let our group guide our own discussion. Let’s start with some initial questions about breastfeeding experiences.”**

1. ***Breastfeeding practice and support mechanism (20 minutes)***
2. **Tell us about your breast feeding experience**

**Participant 1:** During the 7^th^ month of my pregnancy, my breasts started milk secretion and the milk was overflowing. I have two children and for both the pregnancies, I experienced it. One month after delivery, I started having pain in the breasts and I consulted a doctor. The nurses in the hospital tried to extract milk with the help of syringes (She meant breast pump). The milk was extracted for a week and I had pain at the site of the breasts where the pump was applied, I could not bear the pain. Then on the 8^th^ day, I had wound on the breasts where the pump was applied. Afterwards an operation was done on my breast. Even after operation, I had pain. When I got it checked, it was found that there was swelling in the breast. Then the doctors told me to get operated for the swelling again.

**Moderator:** What was your experience of your first childbirth?

**Participant 1:** It started with my first childbirth only.

**Moderator:** You have not breastfed for your second child also.

**Participant 1:** I have breastfed my second child from one breast only.

**Moderator:** For how long you have breastfed exclusively without giving any top feeds?

**Participant 1:** For 6 months.

**Moderator:** From which side you did not breastfeed your babies?

**Participant 1:** From my left side I did not breastfeed. For the first child I breastfed the baby for one month on the left side but after that I could not.

**Moderator:** Did you give any top feed to your baby?

**Participant 1:** I gave Cerelac (commercial top feed) after 6 months.

**Participant 2:** I have only one child of 9 months old. One month after delivery, I had swelling in the breasts on both sides. When I consulted a doctor, I was advised to get operated.

**Moderator:** Are you giving top feed to your baby now?

**Participant 2:** Yes.

**Moderator:** Since when you are giving top feed?

**Participant 2:** Since birth, I am giving goat milk as top feed to my baby.

**Moderator:** How you give top feed to the baby?

**Participant 2:** With a bottle.

**Participant 3:** I breastfed my baby for the first one month only. Thereafter I was not aware of taking care of my baby. I did not feel well and I did not take care of my baby. I lived about myself only. I had breast milk but I did not know that I should breastfeed my baby. Even I was not taking the baby with me. My mother and my sister took care of my baby.

(**NOTE:** This participant 3 had postpartum psychosis).

**Moderator:** What did they feed the baby?

**Participant 3:** They gave bottle milk to my baby.

**Participant 4:** I have two children. I had breast milk for my first child; but I was not having breast milk secretion on the right side. And now also I do not have breast milk secretion from the right side. On the left side, I was having very little breast milk secretion and now that breast also has stopped secretion and I am giving only top milk to my baby.

**Moderator:** Since when you started giving top milk?

**Participant 4:** At the age of 4 months, my second baby was operated for heart problem and since then I am giving top milk. At the time of that operation I expressed my breast milk and thrown it away. Then the breast milk secretion stopped completely.

**Moderator:** Did you breastfeed your baby before it was operated at the age of 4 months?

**Participant 4:** I had little breast milk secretion and used to breastfeed the baby and I was not giving any top milk at that time.

**Moderator:** What about your first child?

**Participant 4:** I breast-fed my first child, but I was not having milk secretion on my right side breast.

**Moderator:** Why did you feel that you have no breast milk secretion from the right side?

**Participant 4:** My first delivery was a caesarean section. After delivery, it was difficult for me to turn on the right side. I used to turn only left side and used to feed my baby from left breast only. After few days when I tried to feed the baby from the right side, the baby did not feed from that side and there was no breast milk secretion from the right side. I feed my 6 months old baby with top milk and cerelac (Commercial top feed)

**Participant 5:** I have two children and for both of them I did not have breast milk secretion after both the deliveries.

**Moderator:** How do you say that you did not have breast milk?

**Participant 5:** I did not have milk in my breasts. After both of my deliveries I was not well and I didn’t have breast milk secretion. I was not knowing about what had happen to me. Both my children are brought up with top feed only.

(**NOTE:** This participant 5 had postpartum psychosis)

**Moderator:** Who took care of your both children?

**Participant 5:** My mother in law took care of my both the children. I was not well and I was not thinking of taking care of my children. Even I was not taking the child to me and I used to leave the children without any care. Therefore, my mother in law herself took care of the babies by top feeding.

**Moderator:** Did this happen to the second child also?

**Participant 5:** Yes, the same thing happened to this second child also, which is now 7 months old.

**Participant 6:** My first child had heart disease and it died at the age of 5 months. My second child is 1 year old and it is also having congenital heart disease. I had breast milk secretion for both the babies but both of them were unable to suck the breast milk. Therefore, I gave them top milk only. For this second child I expressed my breast milk and fed it with spoon for five months. Because of expression of milk, I got swelling in the breast. After that, I gave cow milk till 9 months. Now I am giving buffalo milk to my child.

**Participant 7:** This is my third child, which is five months old. I had breast fed for the first two children. This third child is having cleft palate and cleft lip and it is unable to breast feed, even though I have sufficient breast milk secretion. Therefore, I am giving goat milk.

**Participant 8:** I have two children. For the first child I had no problem in breast-feeding. Two months after the birth of second child, I got infection in the breast and the breast was swollen. I was advised to get operated. I went to a nearby private hospital where I was operated. A lot of puss and blood was taken out from my breast. I got a big wound where the operation was done; for which skin grafting was done. Therefore, I could not breastfeed my baby and I am giving top feed. I gave powder milk to my baby when I was admitted in the hospital and after coming to home, I am giving cow milk and goat milk. I give the top milk with spoon during the daytime and I give the milk with a bottle during the night time because the child cries if the bottle is not given for feeding.

**Participant 9:** I have three children; the first child is 6 years old. And I have twin babies of 2 years age; one is male and the other is female. Both the twin babies were of low birth weight of 1kg. The girl child is breastfeeding but the male child is unable to breastfeed since birth. Therefore, I gave him top milk since birth. My breast milk was not sufficient for both the babies. I gave top milk to the male child with bottle.

**Moderator:** What could be the reasons for the problems of breastfeeding according to you?

**Participant 1:** Because of not breastfeeding the babies properly in the first two months, the breast milk gets stocked in the breast and causes problem.
**Participant 2:** In my case, because I had retracted nipple and a pump was used to take out milk from my breasts. Due to repeated application of breast pump, my breasts became painful. When we consulted a doctor, he told us to express milk from the breasts manually and feed it to the baby with spoon. But the baby started vomiting after feeding the expressed milk.

**Participant 3:** I do not know why I had a problem.

**Participant 4:** I feel that I had pregnancy at my early age.

**Participant 5:** I also do not know why I had this problem.

**Participant 6:** I thought I had the problem because my child had a heart problem and I had small nipples.

**Participant 7:** My child is having cleft palate and cleft lip. Because of that, I have problem in breastfeeding my baby.

**Participant 8:** I had a crack in the nipple because of which I could not breastfeed the baby. Therefore, the milk was stocked in the breast and I had a swelling.

**Participant 9:** I had insufficient breast milk secretion for both of my twin children.

**Breastfeeding attitude**

1. **Do you think breastfeeding is important for the baby?**

**Participant 3:** Mother’s milk is the best.

**Participant 6:** Because it is called as Amrita (The drink of the devas or God, which grants them immortality). Therefore, it has to be fed. And it is good for baby’s health.

**Participant 8:** It is important because the mother feeds her child with love and affection.

**Participant 9:** If the baby breastfeeds it becomes strong and healthy.

**Participant 1:** I do not know much about it.

**Participant 2:** The mother’s breast milk increases the weight of the baby and improves its health.

**Participant 4:** My first child was breastfed. It is healthy and has good body weight; the second child is not breast-fed, and it is thin.

**Participant 8:** If the baby is breastfed, all the characters of the mother and the nutrients are transferred to the baby.

**Participant 1:** Breastfeeding practice is given importance in our culture since ancient days. It is in our Indian culture that every mother should breastfeed her baby for at least 6 months exclusively. And the baby becomes strong and healthy if it is breastfed.

**Participant 7:** Mother’s milk is good for baby’s health and top milk is not good for the baby.

1. **Why? What are the benefits?**

**Participant 9:** If any mother has more breast milk and if she breastfeeds other children who are in need of breast milk it will be helpful.

**Participant 8:** The mother’s breast milk gives energy to the child and it makes the child’s mind sharp.

**Participant 7:** In my case, the child which is breastfed is healthy and the other child which is not breastfed is weak.

**Participant 8:** When my child was breastfed for the first two months it had no problem. But when the child started taking top feed, it started having problems like cough, cold and chest infection frequently.

**Participant 6:** When my child did not take breast milk, it got cough and fever. Now when she reached 7 months she is able to sit and still she is unable to stand up. If she was breastfed, she would not have had these problems.

**Participant 5:** We are giving top feed to my baby and it is having vomiting and diarrhoea. If I had breastfed the baby, it would not have had these problems.

**Participant 4:** I had no breast milk secretion and when I gave top milk to my baby, it used to have chest infection.

**Participant 3:** If the baby is breastfed, it grows well, becomes healthy and will not get any diseases.

**Participant 2:** I do not know about it.

**Participant 1:** If the baby is breastfed, it will not have sputum. My first child was breastfed and it had no problem; but my second child was given top milk and it used to get cough and chest infection frequently.

1. **If you think breastfeeding is not good, what would be the reasons?**

- **This question was not asked.**

1. **How long does the mother need to breast feed exclusively and the total duration?**

**Participant 1:** 6 months.

**Participant 2:** 6 months.

**Participant 3:** 6 months.

**Participant 4:** 1 year.

**Participant 5:** I did not have breast milk.

**Participant 6:** 9 months.

**Participant 7:** 6 months.

**Participant 8:** 6 months.

**Participant 9:** 6 months.

**Moderator:** What should be the total duration of breastfeeding?

**Participant 8:** 2 to 2 and half years.

**Participant 9:** 3 years.

**Participant 7:** 2 to 3 years.

**Participant 6:** 2 to 3 years.

**Participant 5:** 1 year.

**Participant 4:** 1 year.

**Participant 3:** 2 years.

**Participant 2:** 1 year.

**Participant 1:** 2 years.

**Breastfeeding social norm**

1. **Do all of the mothers in the community breastfeed?**

- **This question was not asked.**

1. **Who approves breastfeeding around you?**

- **This question was not asked.**

1. **Who disapproves breastfeeding around you?**

- **This question was not asked.**

1. **What do you and people around you think about prelacteal feeds?**

**Participant 2:** Sugar water is given as prelacteal feed.

**Participant 1:** Honey or Milk is given.

**Participant 3:** If the mother does not secrete breast milk honey is fed to the baby.

**Participant 4:** Honey is given. In my case, I had caesarean. I could not move for a few days after the operation. At that, time honey was fed to my baby.

**Participant 5:** Powder milk and honey was given to my baby soon after delivery.

**Participant 6:** Honey is given as prelacteal feed and the people make the baby to suck dry nipples before actually breastfeeding by its mother. Honey should not be given much. Sucking the dry nipple should be done only for two to three days till the mother starts secreting breast milk.

**Participant 7:** The baby is made to suck the dry nipple of a woman thinking that it should not forget about sucking the breasts. People give sugar water also to the babies at that time. Honey should not be given more because it obstructs the breathing of the child.

**Participant 8:** Honey is given only if the mother does not have breast milk. But it is better to breastfeed even though the breasts are dry.

**Participant 9:** At the time of my first delivery, I did not have breast milk for the first three days. Sugar water was fed to my baby and it was breastfed by another woman who had delivered in the hospital and was admitted beside me.

**Moderator:** Apart from honey what else do the people in your community give as prelacteal feeds?

**Participant 7:** Goat milk and cow milk are also given as prelacteal feeds. My child was unable to breastfeed and it was fed with cow milk.

**Participant 8:** I have seen mothers giving only breastfeeding and not seen any mother giving prelacteal feeds.

**Participant 6:** I have seen people giving powder milk to their babies as prelacteal feeds.

**Moderator:** Have you seen giving powder milk in the hospital or in the home?

**Participant 6:** I have seen it in the hospital only.

**Participant 1:** In majority of cases, only if there is no breast milk secretion, powder milk is given.

164. **What do you and people around you think about colostrum?**

**Participant 1:** The babies will be more healthy if they are fed with colostrum. If it is not fed, the babies will become weak.

**Moderator:** Do the people give it or not?

**All participants (In chorus):** Yes, people give it.

**Moderator:** But just now, you told that people give so many things to babies.

**Participant 1 & 3 (Together):** If the mother doesn’t have breast milk then only people give prelacteal feeds.

**Participant 1:** Already the mother has milk in her breasts before her delivery. Therefore, we should not give prelacteal feeds to the babies.

**Participant 2:** The colostrum should be fed to the babies because it is good for the baby’s health.

**Participant 3:** Yes, it should be given to babies.

**Participant 4:** I did not breastfeed my baby for the first two days as I was operated for caesarean and it was difficult for me to breastfeed. But my baby was breastfed by another mother. The colostrum should be fed to the babies; it is good for the baby as they become healthy and strong.

**Participant 5:** I do not have any idea about it.

**Participant 6:** Colostrum should be fed to the babies so that they will be fine.

**Participant 7:** This colostrum milk is stocked and thick and it should be fed to the babies.

**Participant** **8:** The colostrum milk should be fed to babies.

**Moderator:** Did you feed it to your baby?

**Participant** **7 & 8 (Together):** Yes, I have fed.

**Participant 9:** The colostrum contains protein and it should be fed to the babies.

165. What do you and people around you think about exclusive breastfeeding?

**All participants (In chorus):** Exclusive breastfeeding should be done for 6 months.

166. What do you and people around you think of gripewater /gutti /any medicines during the first six months?

**Participant 6:** People give gutti and gripewater to the babies in addition to breastfeeding.

**Moderator:** Out of 10 mothers how many of you give such things.

**Participant 5:** About five out of ten, give such things.

**Participant 9:** All people give gutti and gripewater to their babies. If the babies are weak and thin, such feeds are given. Even though the babies are healthy, these are fed to them.

**Participant 8:** People are giving dry dates and almond to their babies.

**Participant 7:** Cerelac and gripewater are given to the babies during the first six months.

**Participant 6:** Ragi porridge (Finger millet soup) is also fed to the babies.

**Participant 5:** Rice porridge is fed to the babies.

**Participant 4:** Dry dates and almonds rubbed and made as a paste and fed to the babies.

**Participant 3:** We fed dry dates by rubbing it and making it as a paste.

**Participant 2:** I had given dry date, almond, gripe water and gutti to my baby.

**Participant 1:** I gave dry dates and gripe water to my baby.

**Moderator:** Why these top feeds are fed to the babies during the first 6 months?

**Participant 6:** We feed such things to make the babies healthy and strong. And to make them grow well.

**Participant 1:** If there is any deficiencies in the breast milk such top feeds are given.

**Participant** **2:** These things are fed to improve the baby’s health.

**Participant 3:** To make the baby grow well.

**Participant 4:** If the breast milk is insufficient, these things are given.

**Participant 5:** We fed the baby with rice porridge but it was not digested. Then we stopped giving it.

**Participant 7:** I have given finger millet porridge and top milk to my baby.

**Participant 8:** I had sufficient breast milk but still to improve the baby’s health we gave gutti, dry dates and almond.

**Participant 9:** Even though the babies are breastfed, they become irritant. Therefore, we give these top feeds to make them sedate and sleep well.

**Moderator:** Do all people in the community give such things to the babies?

**All participants (In chorus):** Yes, all people give such things to the babies thinking that it improves the baby’s health.

167. Anything else you would like to add that you think is important?

- (Nobody answered)

**Breastfeeding perceived control**

168. What makes breastfeeding easier?

- This question was not asked.

169. What makes breastfeeding harder?

**Participant 1:** If there is sufficient breast milk secretion and if the baby does not feed all the breast milk secreted, some remaining breast milk gets stocked in the breasts and it creates problems.

**Participant 2:** There was plenty of breast milk with me but as my nipples were retracted, I had the problem.

**Participant 3:** I remember that I breast fed my baby for one month only. Afterwards as I was not well I do not remember anything about it.

**Participant 4:** 20 days after my delivery I came to know that there was a problem with my baby’s heart. Therefore, I got worried about it and I did not have breast milk secretion.

**Participant 5:** I did not have breast milk secretion after delivery. And by seeing other mothers breastfeeding their babies I felt very bad about me.

**Participant 6:** I had enough breast milk but I could not breastfeed my baby as it had a heart problem and was unable to breastfeed properly.

**Participant 7:** I also had sufficient breast milk but as my baby had cleft lip, it could not breastfeed.

**Participant 8:** I had sufficient breast milk and I fed my baby for the first two months properly. After two months, my breasts became infected and I had the problem.

**Participant 9:** I was not having sufficient breast milk for both of my twin babies. Therefore, I could breast feed only one baby and the other baby was top fed.

170. **What does your community think of roles played by maternal depression on breastfeeding?**

- This question was not asked.

171. **What does your community think of roles played by maternal smoking on breastfeeding?**

- This question was not asked.

172. **What does your community think of roles played by maternal drinking on breastfeeding?**

- This question was not asked.

173. **What does your community think of roles played by maternal drug use on breastfeeding?**

- This question was not asked.

174. **What does your community think of roles played by maternal anaemia on breastfeeding?**

- This question was not asked.

175. **What does your community think of roles played by maternal overweight or obesity on breastfeeding?**

- This question was not asked.

176. **What does your community think of roles played by intimate partner violence on breastfeeding?**

- This question was not asked.

K. ***Acceptability of breastfeeding peer counselors (10 minutes)***
85. **Where do you get information about breast-feeding?**

- This question was not asked.

86. **If you have any breastfeeding problem whom do you approach?**

**Participant 1:** I approached my grandmother in the home who used to apply oil and massage my breasts. I also approached the nurses working in the villages who used to come and extract the milk with syringe.

**Participant 2:** I approached my grandmother in the family; I also consulted a doctor who advised me to express the milk and feed it to the baby. But the baby could not digest it and it started vomiting.

**Participant 3:** I had breast milk but I could not breastfeed it. Therefore, my breast milk used to over flow.

**Participant 4:** I approached my grandmother. I consulted a doctor also, who prescribed me tablets and powder. Even then, I could not secrete breast milk.

**Participant 5:** We consulted a local doctor and he prescribed a milk powder for my baby.

**Participant 6:** I had swelling in the breast because I could not breastfeed my baby properly.

**Participant 7:** My baby has cleft palate and cleft lip and we have consulted a doctor in tertiary care hospital who has advised surgery for the baby.

**Participant 8:** I approached an old woman in our community who used to massage my breasts so that I could secrete breast milk properly.

**Participant 9:** We consulted a doctor in the government tertiary care hospital who advised us to give powder milk to my one of the twin baby.

87. Are you aware of any programmes by the government to promote breast feeding practices in the country?

- This question was not asked.

88. Is there any support of the women from the village/community?

**Participant 6:** There are ANMs in our village. When we tell them about our problem, they come and help us. Old women in the village also come to our help.

**Participant 9:** ASHA workers are in our village, who help us when we have any problem.

**Participant 3:** Our mothers and grandmothers help us.

**Moderator:** Do all of you agree that the old women in the village come to your help?

**All participants (In chorus):** Yes, the old women come to our help.

89. Is she trained in giving breast feeding support and help?

**Moderator:** You told that, the old women in the community and the grand mothers help you whenever you are in problem. Are they trained in giving breastfeeding support and help?

**Participant 3:** They help us with the traditional methods they are following from many years and with their experience.

**Moderator:** Are they trained in giving support and help in breastfeeding?

**All participants (In chorus):** They are not trained in giving breastfeeding support and help.

**Breastfeeding peer attitude**

90. Do you think that a person (Peer Counsellor) from your community/village, if trained will be helpful to solve the breastfeeding problems?

**All participants (In chorus):** Yes, it will be helpful in breastfeeding problems.

**Participant 1:** If such a person was there in our community and had come and helped me, I would not have gone for the operation.

**Participant 2:** Yes, if such a person is there in our community, it will be helpful.

**Participant 3:** Yes, it will be helpful.

**Participant 4:** Yes, it will be helpful.

**Participant 5:** Yes, it will be helpful.

**Participant 6:** Yes, it will be helpful.

**Participant 7:** Yes, it will be helpful.

**Participant 9:** Now ASHA workers are there in the community; similarly if a person is there in the community, it will be helpful.

**Participant 8:** Like ASHA worker if a person was there in the village and if she had helped me, I would not have faced a situation of undergoing surgery of my breasts.

91. Do you accept receiving Breastfeeding counselling and advice from such a person?

**Moderator:** You told that if a trained person was there in the community, it would have been helpful. We call such a person as “peer counsellor”. Do you accept receiving advice from such a person?

**All participants (In chorus):** Yes, we will accept advice from such a person.

**Participant 1:** Yes, I accept.

**Participant 2:** Yes, I accept.

**Participant 3:** Yes, I accept.

**Participant 4:** Yes, I accept.

**Participant 5:** Yes, I accept.

**Participant 6:** Yes, I accept.

**Participant 7:** Yes, I accept.

**Participant 8:** Yes, I accept. It will be helpful.

**Participant 9:** Yes, I accept. It is good if such a person is in the village.

92. What should be the qualifications of Peer Counsellor?

**Participant 1:** She should have a basic education and knowledge about breastfeeding.

**Participant 2:** (Did not answer)

**Participant 3:** She should have education and experience of breastfeeding.

**Participant 4:** She should have education and knowledge about breastfeeding.

**Participant 5:** She should be well behaving.

**Participant 7:** She should be having good characters.

**Participant 6:** She should have knowledge about breastfeeding.

**Participant 8:** She should have education, experience and knowledge about breastfeeding.

**Participant 9:** She should have patience; even if somebody comments about her she should carry out her work without bothering about the comments by others.

**Participant 3:** She should be nice in talking.

**Moderator:** Where she should be staying?

**Participant 9:** One person in each lane of the village.

**Participant 8:** One person in each lane of the village.

**Participant 7:** It will be okay if she is near our lane.

**Participant 6:** If at least one person is there in the village, it will be okay.

**Participant 5:** If she is in the village, it is sufficient.

**Participant 4:** In our lane of the village.

**Participant 3:** It is still better if she is by the side of our house (laughs and tells jokingly).

**Participant 2:** It is okay. If she is in the village.

**Participant 1:** She should be in the village.

**Breastfeeding peer perceived control**

93. What would be the benefit to have Peer Counsellor?

**Participant 1:** If a breastfeeding woman gets pain in the breasts, the peer counsellor can take her to the hospital or she may treat her in the home.

**Participant 2:** The peer counsellor will be having all the knowledge about breastfeeding and whatever she advices us, we need to listen to her to have good breastfeeding.

**Participant 3:** Many future problems can be prevented.

**Participant 6:** If a mother does not know how to breastfeed, the peer counsellor will help her in breastfeeding. She can tell the breastfeeding mother how to hold the child, how to make the child to suck the breasts and all the things which are good for her health.

**Participant 4:** My own sister in law (husband’s sister) is there who helps me in breastfeeding my baby.

**Participant 5:** The peer counsellor will help in feeding the baby.

**Participant 7:** The peer counsellor will help us in taking us to the hospital, if we have any problem in breastfeeding and if there is any problem to the baby.

**Participant 8:** We can solve our problems of breastfeeding with the help of peer counsellor. Some people who do not have money to go to hospital can get their problem solved locally by the peer counsellor.

**Participant 9:** If a peer counsellor is there in the community, she can advise us in going for higher-level care like getting operation.

94. What would be the barriers/problems to have Peer Counsellor?

**All participants (In chorus):** There will not be any barrier or problem to have peer counsellor in the community.

**Breastfeeding peer social norm**

95. Who approves the presence of Peer Counsellor?

**Moderator:** Do all the people in your community accept the presence of such peer counsellor?

**Participant 1:** Yes, all the people will accept the presence of peer counsellor.

**Participant 2:** Yes, all will approve.

**Participant 3:** Yes, all will approve.

**Participant 4:** Yes, all will approve.

**Participant 5:** Yes, all will approve.

**Participant 6, 7, 8 & 9:** Yes (All nod their head in approval).

96. Who disapproves the presence of Peer Counsellor?

**Participant 3:** Few people disapprove. They are the persons who have personal dislike towards the presence of peer counsellor.

**Participant 6:** Very rarely, there may be one or two such persons in the community.

**Participant 1:** Some local medical practitioners may not like the presence of peer counsellor, as they are afraid that the number of patients going to them may decrease. Such people may create problems. But there will not be any problem from the community.

**Participant 5:** Some patients will go to the local practitioners whatever may be the situation.

**Participant 8:** The local doctor may feel that they may get less patients.

**Participant 6:** There may be some persons in the neighbourhood, who have personal dislike towards the peer counsellor.

L. ***Program to train breastfeeding peer counselors (10 minutes)***
109. **How does the health worker help you in breast feeding activities?**

**Participant 9:** Some mothers who have delivered for the first time may not be knowing how to breastfeed. For such mothers the ANMs (health worker) help her in how to position the baby while breastfeeding and such other things.

**Participant 8:** Health workers advice in breastfeeding.

**Participant 6:** They advise the mother to breastfeed regularly and to hold the baby on the shoulder after breastfeeding. They also tell the mother to keep the baby warm by holding the baby on the chest.

**Participant 7:** The mothers who have delivered for the first time may not be knowing how to breastfeed. For them the health worker demonstrates practically how to breastfeed the baby.

**Participant 1:** The health worker advises the mothers to keep the baby warm. If the breast milk is stored in the breasts in excess, they advise her to express the stored breast milk and keep it for feeding the baby.

**Participant 2:** After the first delivery the health worker demonstrates the mother how to breastfeed, how to hold the baby on the shoulder and rub the baby on the back with hand for burping.

**Participant 3:** The health workers advise the mothers to breastfeed in sitting position and how to hold the baby on the shoulder for burping.

**Participant 4:** The health workers advise the mothers to breastfeed in sitting position and not to breastfeed in lying down position.

110. Does the health worker visit and advise you about Breast feeding before delivery?

AND

111. What does the health worker talk about breastfeeding antenatally?

**Moderator:** Does the health worker visit you when you were pregnant and did she tell anything about breastfeeding?

**Participant 7:** They came and visited us but did not tell anything about breastfeeding.

**Participant 9:** Yes, the health workers tell us about breastfeeding during antenatal period. They tell us about massaging the breasts while taking bath so that the nipples do not get retracted and it will be easy for breastfeeding after delivery.

**Participant 8:** They have not told us anything about breastfeeding during antenatal period.

**Participant 6:** They have told us about breastfeeding.

**Participant 5:** No, they have not told us about breastfeeding.

**Participant 4:** Yes, they used to tell us to massage the breasts applying oil.

**Participant 3:** They used to tell me not to wear tight dresses.

**Participant 2:** No, they have not told us anything about breastfeeding during pregnancy.

**Participant 1:** They used to tell me not to wear a very tight bras and dresses.

**Moderator:** Did they examine you about whether the nipples are normal or retracted? About when to start breastfeeding?

**Participant 1:** No.

112. What happens at birth? Is she present at birth and help you with early starting of Breast Feeding?

**Moderator:** Do the health workers advise the mother to place the baby to the breast immediately after delivery?

**All participants (In chorus):** Yes, they advise.

**Participant 1:** The ASHA worker stays with us from the time of labour pains till the delivery occurs and till we go to our home from the hospital.

**Moderator:** What happens when you go to your home after delivery?

**All participants (In chorus):** The health workers come to our house to see us.

**Participant 8:** There are our mother and grandmothers in the house who take care of us.

**Participant 6:** ASHA workers come to see us.

**Participant 5:** They come to see us.

**Moderator:** What do they do when they come to see you?

**Participant 6:** They tell us to hold the baby properly to breastfeed in sitting position and to hold the baby on the shoulder for burping.

**Participant 5:** They tell us to take good care of the baby.

**Participant 1:** They tell us to put coconut oil in the ear and on the head whenever the child gets cold. They also tell us not to bathe the child till 21 days if its weight is less.

**Moderator:** Do they tell anything about breastfeeding?

**Participant 1:** They tell us to eat green vegetables so that we get good breast milk secretion.

113. How frequently does she visit you after birth of the baby?

AND

114. What is the purpose of visit?

**Participant 8:** The health workers have not come to our house as we are residing in our farmhouse.

**Participant 9:** They come to us regularly. Twice in a month.

**Participant 6:** ASHA workers come to us regularly.

**Participant 7:** They come to our house also.

**Participant 5:** ASHA workers come to our house also.

**Participant 4:** We are staying in our farmhouse away from the village and the health workers are in the village; therefore, they do not come to our house frequently.

**Participant 3:** Yes, they come regularly to our house.

**Participant 2:** Yes, they come.

**Participant 1:** Yes, they come.

**Moderator:** When the health workers come to you, do they tell you about breastfeeding?

**Participant 3:** They advise us to breastfeed the baby.

**Participant 1:** They tell us to breastfeed but they do not tell how to breastfeed in detail.

**Participant 6:** They advise us not to stop breastfeeding.

**Participant 9:** They tell us to breastfeed frequently once in every hour.

**Moderator:** If there is any problem in breastfeeding, do they try to do something to solve it?

**Participant 3 & 6:** Nothing, they do not do anything. Only they give some tablets.

**Participant 8:** They prescribe medicines.

**Moderator:** When the mother delivers for the first time, she may not know how to position the baby to breast feed. And if there is any problem in breastfeeding, do they come and help?

**All participants (In chorus):** Yes, they come and help us.

**Moderator:** After delivery, approximately how many times the health workers come to your house?

**Participant 6:** ASHA worker is staying near my house. She comes about once in two days.

**Participant 1:** They come once in fortnight or once in a month.

**Participant 8:** Whenever there is due date of vaccination of my baby, the ASHA workers come to us to take me for vaccination.

**Moderator:** Whenever the health workers come to your house, do they enquire about your breastfeeding?

**Participant 1:** No, they do not enquire about breastfeeding. Only they do whatever they are supposed to do as per their duty.

**Participant 6:** They do not give any information about breastfeeding. Only they give vaccination injections and go.

**Participant 9:** They advise us to breastfeed the baby frequently and regularly.

115. How frequently does she assess your breast-feeding practices?

**All participants (In chorus):** No, they have not assessed our breastfeeding practices.

**Participant 8:** (Laughs) The grandmothers have a superstition that no one should see the mother breastfeeding her child.

**Participant 1:** The grand mothers have a misconception that if anybody sees a mother breastfeeding her child, they think evil eye falls upon her. Therefore, the health workers just enquire about breastfeeding without actually looking at the breastfeeding procedure and without touching the breasts.

116. How many mothers does the health worker visit per day/per month?

- This question was not asked.

117. What are the common breast feeding problems you observe in your community?

- This question was not asked.

118. Do you think that peer counsellor who is from the same community would help and support mothers?

**Moderator:** You told that some grandmothers do not allow the health workers or anybody else to see the breastfeeding of a woman or touching and examining her breasts. But if a peer counsellor who is from the same community, having good nature, well experienced, well trained, with good communication skill and good characters is appointed in the community to counsel the breastfeeding mothers, do they accept such peer counsellor and allow her to see the breastfeeding mother?

**Participant 1:** In normal circumstances, even though the grandmothers do not allow them to see breastfeeding mother, if the breastfeeding mother has any problem, they themselves will call the peer counsellor for help and they will listen to her advice (laughs). When they have any problem, they approach a person from whom they get the solution.

**Participant 8:** They call her for help so that no problem should come in future.

**Moderator:** Do you all of you agree with what participant 1 has just said?

**All participants:** Yes, we agree.

**Participant 9:** The elders in the family say, “Let her see the breastfeeding; she knows everything” but there are some non-cooperative grandmothers who say, “What they say? it is all nonsense”.

**Participant 7:** Some grandmothers who are having the old thinking do like this.

**Moderator:** Do they accept the peer counsellor?

**Participant 4:** They accept the peer counsellor because they have all the knowledge and experience.

**Participant 1:** Some grandmothers about 10 in 100; do not accept. But the present generation will accept the presence of peer counsellor.

119. What do you think would be the barriers for Peer Counsellors in the community?

- This question was not asked.

120. Will there be future opportunities for these Peer Counsellors to continue in the community?

**Participant 1:** Yes, they have future opportunities.

**Participant 4:** Yes. They will be very useful in future.

**Participant 3:** Yes.

**Participant 5:** Yes.

**Participant 6:** Yes.

**Participant 7:** Yes.

**Participant 8:** Yes.

**Participant 9:** Yes.

**Participant 2:** Yes.

121. What should be the criteria for acceptance of such counsellors by you and the community?

**Participant 1:** It can be assessed by their personality; how they speak patiently and whether they take any bribe for the home visit.

**Participant 3:** By looking at her work, how she has worked with previous mothers, we can assess her performance.

**Participant 1**: We should have her phone number. When we call her by phone or when we go to her personally and call her, we need to see her response. Whether she responds positively or not. By that, we can assess her performance.

**Participant 6:** If she stays locally and takes good care of us, then we can say that her work is good.

**Participant 7:** If she takes good care of us, we think that she is good.

**Participant 9:** Only we can tell you, how she is working.

**Participant 8:** The mothers who faced problems can tell about the work of the peer counsellor.

**Participant 3:** She should be having good characters and manners.

**Participant 1:** She should be having patience.

**Participant 9:** Even if somebody behaves rudely with her, she should tolerate it.

**Participant 8:** She should be able to solve the problems of breastfeeding mother.

**Participant 7:** She should listen to the problems of the mothers frequently.

122. How do you identify and recruit such counsellors?

**Participant 1:** Yes, some persons who have the talent.

**Participant 3:** Some persons have more knowledge. They travel more and interact with others frequently.

**Participant 8:** Some have general knowledge.

**Moderator:** Can we identify such persons?

**All participants (In chorus):** Yes, such persons can be identified.

**Moderator:** How to recruit such counsellor?

**Participant 1:** We can select a person whom majority of the local people agree.

**Participant 9:** We, the mothers can identify such a person.

**Participant 3:** The elders in the community can help us in identifying such persons.

**Participant 8:** ASHA workers can help in this and they themselves can be recruited as peer counsellor by giving some more training to them. Because they are already having some knowledge about it.

**Participant 7:** ASHA workers can also do this job as they are already having some knowledge about it.

**F. Wrap up**

15. *Ask participants*

**“How did you feel about participating in this session? What was easy? What was hard?”**

**All participants (In chorus):** We felt that the discussion was good.

16. *Summarize and thank participants by saying:*

**“We appreciate all of your great ideas. You have been a big help, and we want to thank you very much for all the information you have shared with us today. We know that your ideas will help to make this a successful program and technology. Thank you once again for your participation – we really appreciate you!”**

**Moderator: Thank you.**
